# A mobile health monitoring-and-treatment system based on integration of the SSN sensor ontology and the HL7 FHIR standard

**DOI:** 10.1186/s12911-019-0806-z

**Published:** 2019-05-10

**Authors:** Shaker El-Sappagh, Farman Ali, Abdeltawab Hendawi, Jun-Hyeog Jang, Kyung-Sup Kwak

**Affiliations:** 1 Department of Information and Communication Engineering, Inha University, Incheon, South Korea; 2 Information Systems Department, Faculty of Computer and Informatics, Benha University, Banha, Egypt; 3 Computer Science, University of Virginia, Charlottesville, USA; 4 Faculty of Computers and Information, Cairo University, Giza, Egypt; 5 Department of Biochemistry, School of Medicine, Inha University, Incheon, 400-712 South Korea

**Keywords:** Clinical decision support system, Semantic interoperability, Ontology, Mobile health, Diabetes treatment

## Abstract

**Background:**

Mobile health (MH) technologies including clinical decision support systems (CDSS) provide an efficient method for patient monitoring and treatment. A mobile CDSS is based on real-time sensor data and historical electronic health record (EHR) data. Raw sensor data have no semantics of their own; therefore, a computer system cannot interpret these data automatically. In addition, the interoperability of sensor data and EHR medical data is a challenge. EHR data collected from distributed systems have different structures, semantics, and coding mechanisms. As a result, building a transparent CDSS that can work as a portable plug-and-play component in any existing EHR ecosystem requires a careful design process. Ontology and medical standards support the construction of semantically intelligent CDSSs.

**Methods:**

This paper proposes a comprehensive MH framework with an integrated CDSS capability. This cloud-based system monitors and manages type 1 diabetes mellitus. The efficiency of any CDSS depends mainly on the quality of its knowledge and its semantic interoperability with different data sources. To this end, this paper concentrates on constructing a semantic CDSS based on proposed FASTO ontology.

**Results:**

This realistic ontology is able to collect, formalize, integrate, analyze, and manipulate all types of patient data. It provides patients with complete, personalized, and medically intuitive care plans, including insulin regimens, diets, exercises, and education sub-plans. These plans are based on the complete patient profile. In addition, the proposed CDSS provides real-time patient monitoring based on vital signs collected from patients’ wireless body area networks. These monitoring include real-time insulin adjustments, mealtime carbohydrate calculations, and exercise recommendations. FASTO integrates the well-known standards of HL7 fast healthcare interoperability resources (FHIR), semantic sensor network (SSN) ontology, basic formal ontology (BFO) 2.0, and clinical practice guidelines. The current version of FASTO includes 9577 classes, 658 object properties, 164 data properties, 460 individuals, and 140 SWRL rules. FASTO is publicly available through the National Center for Biomedical Ontology BioPortal at *https://bioportal.bioontology.org/ontologies/FASTO*.

**Conclusions:**

The resulting CDSS system can help physicians to monitor more patients efficiently and accurately. In addition, patients in rural areas can depend on the system to manage their diabetes and emergencies.

**Electronic supplementary material:**

The online version of this article (10.1186/s12911-019-0806-z) contains supplementary material, which is available to authorized users.

## Background

The number of people suffering from chronic health conditions is increasing. In 2008, non-communicable diseases like diabetes were responsible for 63% of all deaths all over the world [1]. Chronic disease management places considerable pressure on patients, healthcare systems, and communities worldwide [2]. Treatment of these diseases usually takes long time and costs a lot of money. Because of societal aging and the increased number of patients with chronic conditions, more and more people will require long-term personalized medical care. Diabetes mellitus (DM) is a chronic metabolic disease. It is a major healthcare problem even among the most developed countries. In 2015, an estimated 1.6 million deaths were directly caused by DM, and it is expected to be the seventh leading cause of death in 2030 (*http://www.who.int/news-room/fact-sheets/detail/diabetes*). If the current trend continues, one in three Americans will have diabetes by 2050 (*http://www.diabetes.org*).

The most serious type of DM is type 1 (T1D). It is an autoimmune disease where the body destroys the insulin-producing *β* cells in the pancreas. Patients with T1D do not produce any insulin, and must exogenously inject this hormone four to six times per day to keep blood glucose levels under control [3]. People with T1D need to check their glucose level several times per day, called continuous glucose monitoring (CGM) [4]. Based on these monitoring data, as well as other factors (e.g. meals and exercise), they can decide what types of insulin they need, when to inject them, and how much; what types of food to eat, and in what quantities; and what types and intensities of exercise to engage in. Insulin may be combined with other medications, such as metformin, pramlintide, blood pressure drugs, cholesterol drugs, aspirin, and other medications related to the patient’s complications. These medications have side effects, and they can conflict with each other, with diseases, or with foods. As a result, creating a customized treatment plan (TP) is a complex process, and if not done carefully will result in serious short-term and long-term complications [5]. Short-term complications include hypoglycemia and hyperglycemia; long-term complications include autoimmune diseases, dyslipidemia, retinopathy, cardiovascular diseases, nephropathy, and neuropathy. Patients cannot make these crucial decisions solely, and always need to consult healthcare professionals. The healthcare team (ophthalmologist, endocrinologist, dietitian, pharmacist, dentist, and educator) studies the entire patient profile and suggests tailored TPs for specific periods.

Handling this challenge requires a medical expert to be reachable to the patient constantly, or the patient has to be hospitalized at all times. Neither of these options is practical. With the ever-increasing world population, the conventional patient–doctor appointment has lost its effectiveness because resources are not available for such monitoring and hospitalization. To overcome the limitations of existing hospitals and doctors, technology can play a vital role. An artificial pancreas can be utilized by diabetics aged 14 or older. It is a closed-loop control system composed of a CGM device checking the patient’s glucose level in real time (e.g. every 5 min) and injecting insulin accordingly [6]. Although this device monitors some biometrics in the patient’s body, considering other features (including complications, medications, demographics, and symptoms) is critical. For correct interpretation of monitored vital signs, they must be understood in the context of the entire patient profile [7, 8]. For example, the sensed blood glucose (BG) level is sometimes high, but the patient may take drugs that are the main cause of this rise such as *steroids*, *anti-psychotics*, *corticosteroids*, *statins*, *niacin*, *antipsychotics*, and *decongestants* [9]. In addition, the patient may suffer from other diseases that increase BG levels, such as *pancreatitis*, *hypercortisolism*, *pancreatic cancer*, *gingival disease*, and *stroke* [10]. As a result, making insulin-injection decisions based only on sensed blood glucose level is not sufficient.

A new approach that demonstrates improved well-being and quality of life is mobile health (MH) [11]. MH supports continuous remote monitoring of blood glucose, which is essential for an insulin therapy regimen [12]. There are many choices when implementing MH for continuous patient monitoring [13]. Patients can be monitored 24 h a day by manually entering biomedical parameters; the collected data are sent to medical experts who provide advice regarding treatment. However, this approach is not suitable because asking patients to enter many values is not convenient and is error prone. Furthermore, this process increases the medical expert’s workload, and he or she may not reply to the patient on time. A clinical decision support system (CDSS) is a knowledge-based system that can mimic medical experts in data analysis and decision-making. It can automate the monitoring process, reduce medication errors, and improve quality of care. Mobile patient-monitoring CDSSs based on medical sensors, mobile, and wearable devices support the implementation of this solution [14, 15]. The mobile phone becomes a ubiquitous tool with nearly 100% availability in developed countries [12]. These devices have recently gained powerful computing capabilities and enable open application development. Klasnja and Pratt [16] discussed the factors that make the mobile phone a promising platform for health interventions. In addition, the recent advances in information and communications technology infrastructures, including wireless communications, cloud computing, and big data analytics provide promising techniques for developing MH systems. They transform healthcare ecosystems from hospital-centered to patient-centered, and remotely involve patients in their health monitoring process. Mobile patient monitoring was defined by Pawar et al. [17] as “*the continuous or periodic measurement and analysis of a mobile patient’s bio-signals from a distance by employing mobile computing, wireless communications, and networking technologies*.” With this major shift, MH systems detect, monitor, prevent, and control chronic diseases by providing “*anywhere and anytime*” healthcare scenarios. However, to this date, most clinical care continues to be provided without the aids of CDSSs [18] because patients and medical experts do not believe in CDSS decisions.

A comprehensive MH CDSS should be based on two main sources of data: real-time sensor data and historical electronic health record (EHR) data [15]. Current MH studies for diabetes management are based on monitored vital signs [19] solely without giving attention to the complete EHR [15]. Consequently, the decisions resulting from these studies are misleading and not medically acceptable. That is because raw vital-sign observations do not provide the context required for interpreting those observations properly. Vital-sign observations have different meanings depending on the context, i.e., the historical conditions of the patient collected from distributed EHR systems [7]. Collecting, modeling, and reasoning with sensor data in the context of the EHR play critical roles in tackling the MH CDSS challenges. However, integration of heterogeneous sensor and historical medical data is a complex task [8, 18]. In addition, integration of CDSS knowledge with the EHR ecosystem is another burdensome.

Having said that, our pursuit in this project is to devise an interoperable MH framework suitable for mobile diabetes monitoring and to provide customized, long-term, and real-time treatment plans (TPs). These plans are created according to integrated real-time patient vital-sign data with collected historical profile. *No study in the literature propose complete TPs for T1D including insulin, diet, exercise, education, and emergencies.* To guarantee the plug-and-play capability, semantic interoperability is handled based on the HL7 fast healthcare interoperability resources (FHIR) standard for data storage and communications and for knowledge representation. The framework has four different modules, namely *patient module, cloud-based CDSS module, backend EHR systems module, and mobile health services module*. *The patient module* is for mobile monitoring of the patient based on a set of sensors. Every patient has a wireless body area network (WBAN) to collect biomedical signs. These data are integrated with distributed historical EHR data stored in the cloud, based on the FHIR standard. *The cloud-based CDSS module* collects, integrates, and interprets patient data and proposes TPs. The integration of different data formats is based on semantic annotation of sensor data based on the semantic sensor network (SSN) and basic formal ontology (BFO) ontologies, standardization of medical data based on the FHIR standard, and binding with standard medical terminologies. *The backend EHR systems module* is responsible for collecting the patient’s historical data from distributed EHR systems. *The services module* provides a collection of services for patients and physicians including real time guidance, provision of TPs, and emergencies. Building a representative, accurate, and complete CDSS knowledge base is the most important step toward generating a medically acceptable CDSS. We describe in full details the development process of a unified semantic model called FHIR and SSN-based T1D Ontology (FASTO), which is a standard, modularized, interoperable, and comprehensive OWL 2 medical ontology. FASTO integrates the semantic capabilities of the SSN, BFO, FHIR, clinical practice guideline (CPG), and medical terminologies in a unified, homogeneous, and intelligent manner. All FASTO knowledge is collected from the most recent CPGs [20]. Combination of FASTO and OWL 2 reasoner such as Pellet implements the semantically intelligent CDSS. Thanks to the FHIR standard, ontology semantics, and medical terminology, we believe the proposed MH framework can enable broader adoption of and transparent integration with already implemented EHR systems.

In the rest of this paper, we review the related work in Section 2. We then briefly present the proposed MH framework in Section 3. In Section 4, we discuss the patient and services modules. Section 5 discusses the CDSS module and the FASTO construction process. Section 6 details the backend systems of the proposed CDSS. Section 7 evaluates the proposed semantic ontology, and Section 8 provides a discussion about the paper findings and limitations. Finally, Section 9 concludes the paper with a discussion of future work.

## Literature review

### Diabetes and mobile health

The majority of chronic disease CPGs recommend the inclusion of self-management programs in routine disease management [11, 20]. However, limited research has been done in this domain. Brzan et al. [21] evaluated 65 apps based on four measures: [1] monitoring blood glucose levels and medications, [2] nutrition, [3] physical exercise, and [4] body weight. They concluded that 56 of these apps did not meet even minimal requirements, or did not work properly. They concluded that only nine apps could be versatile and useful enough for successful self-management of diabetes. They asserted that a CDSS app must be connected to an EHR system, and it must support interoperability. Basilico et al. [22] evaluated 952 mobile apps for diabetes management and concluded that none of them provided complete TPs, or even insulin calculators. As a result, their adoption in the real world is limited. Rose et al. [23] asserted that existing diabetes monitoring studies have not provided DM management in a standard manner. Fatehi et al. [24] concluded that existing MH apps provide fragments of care plans for diabetes, and asserted that the roles of a CDSS and an EHR are needed to facilitate accurate care. Recently, Caballero-Ruizet al. [5] asserted that the current limitations in telemedicine systems for diabetes include usability, real-time feedback, and decision support capabilities. Cappon et al. [4] reviewed the wearable CGM sensor technologies including commercial devices and research prototypes. They discussed the role of CGM to improve CDSSs and big data analytics for personalized medicine. They asserted that the integration of CGM massive data collected by low cost sensors with EHR historical data would be essential to develop new strategies for personalized diabetes management. Quinn et al. [25] proposed a glucose-monitoring system called WellDoc. This system only collects glucose readings and physical activity data from type 2 diabetics, and uploads them to a server where a physician can give feedback by email. There are no CDSS features in WellDoc; as a result, we cannot consider it as a MH system. In the absence of a CDSS, the clinician must [1] study patients’ big data, [2] identify trends and correlate related changes in these data for all patients with failing health, and [3] contact those patients who possibly need intervention. Existing T1D MH approaches are standalone applications that provide partial capabilities that are not sufficient [24]. Some studies concentrated on the collected glucose data from sensors only to determine new insulin doses and types of insulin; other studies concentrated on lifestyle programs. In the following, we discuss some of these studies. COMMODITY12 is the most famous multi-agent CDSS for diabetes treatment [26]. The system provides treatment for type 2 diabetics, but we will concentrate on type 1 diabetics. COMMODITY12 has not handled the semantic interoperability between different system’s components including backend database and sensor data. In addition, the quality of its proposed TPs is not acceptable in medical domain because the system has not considered the whole patient’s medical history [27]. Keith-Hynes et al. [28] proposed DiAs, a smartphone-based system for T1D monitoring. However, this research is very abstract and only discusses the structure of the proposed framework. Kan et al. [11] proposed the ubiquitous health management system for diet (UHMS-HDC), which includes a diet diary and nutritional guidance. This system is based on a relational database (RDB), and there are no semantic inferences. In addition, this system only works as a standalone application because it does not handle interoperability. Su et al. [29] proposed a CDSS to generate personalized exercise plans based on an ontology and HL7 v3. However, they ignored the related issues of diet and medicine. Schmidt and Norgaard [30] proposed a bolus calculator app that determines a bolus dose based on an equation. These types of system are not medically acceptable because bolus dose must be part of a chronic and continuous plan. All of the discussed studies proposed partial solutions to the MH challenge, and all have critical limitations. Some studies have not handled interoperability such as COMMODITY12, UHMS-HDC, and DiAs. Others have handled only parts of the problem such as Su et al. for exercise plan management. In addition, most of these studies proposed systems for type 2 diabetes, which is very different from type 1. As a result, Greenes et al. [31] concluded that wide adoption and broad use of a CDSS in clinical practice has not been achieved.

The more suitable solution is to automate the treatment process based on a CDSS, which reduces face-to-face visits, and keeps patients from unnecessary displacements. This way, medical experts optimize their time, and can concurrently manage hundreds of patients in a more efficient way [5]. There are two options for implementing this solution. *In the first option*, the patient continuously collects WBAN biometrics on a mobile device and uses a local CDSS for direct monitoring and suggesting of TPs. However, smart phones do not have enough storage, processing, memory, and battery resources to process the data generated from sensors, and to give real-time decisions. In addition, a real CDSS needs other patient data from an EHR, where interoperability is a major problem. *In the second option*, all patient data are collected in the cloud from heterogeneous sources and are integrated with distributed hospital EHR ecosystems. Cappon et al. [4] asserted the role of data integration to implement accurate CDSSs. The resulting model can provide timely assistance, supports scalability of data storage and processing power, and supports global accessibility by any number of patients and physicians at any time and from any place.

A WBAN is a special-purpose wireless sensor network that incorporates different networks and wireless devices to enable remote monitoring in various environments. Internet of Things (IoT) based systems have been used in different fields in the medical domain. Szydło and Konieczny [14] proposed a system for cardiovascular diseases; however, this system takes decisions based on the sensed data only, and does not take the full patient history into account. The WANDA. B monitoring system [32] provides an integrated architecture to monitor heart failure patients in real time. Unfortunately, it does not provide support for medication dosages and individual health plans. The MyHeart Project [33] is a mobile system to remotely monitor heart failure patients based on wireless sensor networks. However, the system does not support medicine intake management and sophisticated treatment plans. Regarding diabetes, a review of smartphone, IoT, cloud, and WBAN applications designed to help in diabetes management was presented in [34]. The IoT was proposed as a good environment for diabetes management in [35]. Cloud computing systems for diabetes control were discussed in [36]. However, we can see that, so far, applications are limited, and they focus on some specific part of management (tracking physical activity, glycemic control, etc.), but there is no complete perspective on the problem [27].

### The ontology and mobile health

Ontology plays an important role in building intelligent, distributed, and interoperable CDSSs because it provides explicitly formal and uniform semantic models [2, 29, 31, 37]. The ontology is a knowledge representation formalism, where the resulting knowledge is sharable, manageable, accessible, understandable, and processable by machine [8]. It is based on formal description logic such as SROIQ (D), an ontology language such as OWL 2, a rule language such as SWRL, and a query language such as SPARQL. Its semantic reasoning process is based on semantic reasoners such as Pellet, Fact++, and Hermit [38]. A standard ontology supports personalized reasoning, knowledge sharing, automatic reasoning, and semantic interoperability between heterogeneous sources [39]. Personalized service is the provision of the “*right*” information for the “*right*” user at the “*right*” time and in the “*right*” way. It provides evolving and tailored assistance to a user based on her/his unique medical profile. Kan et al. [11] proposed a ubiquitous health management system for healthy diets without using ontology, so the proposed system suffered from interoperability issues as asserted by the authors in their study. There are limited diabetes ontologies in the literature. Our DDO [9] and DMTO [10] are most complete and medically intuitive diabetes ontologies in the literature. They are designed with the interoperability in mind. As a result, we extend the knowledge of these two ontologies in our current study. Esposito et al. [2] proposed a four-tier smart mobile and context-aware architecture to support the rapid prototyping of MH applications for different scenarios; the system is mainly based on the processing capabilities of the mobile phone. For interoperability, Esposito et al. depended on a local data model based on an ontology. Although the ontology can support semantic interoperability, careful design is critical where the ontology must be based on standards [40]. There are considerable challenges facing the useful implementation of a successful ontology-based CDSS for mobile patient monitoring [41]. These challenges include how to extract medical knowledge from CPGs, how to formalize this knowledge as OWL2 axioms and rules, how to integrate sensor data standards with EHR data modeling standards, how to collect patient profile from distributed hospitals in a standard form, and how to build complete TPs that can provide real-time and long-term assistance. Lanzola et al. summarized the relevant approaches in this field [42]. Esposito et al. [2] asserted that existing mobile health proposals do not handle the real MH challenges, and they listed some of them as semantic interoperability and integration challenges. The integration challenges of heterogeneity in EHR systems and IoT data in a cloud environment were explained in [43]. None of the current studies provides a complete platform for T1D management [27].

### Interoperability and mobile health

To monitor patients more accurately, sensor-based vital signs must be interpreted in the context of the entire patient profile. Patient data are always distributed, encoded with different medical terminologies, and structured with different “standard” data models [39]. Interoperability techniques can help to integrate and share these heterogeneous data sources. Please notice that interoperability is not the main focus of this paper; however, we believe it is a main requirement to develop an acceptable CDSS. Most mobile app studies propose standalone frameworks, and this is one of the main reasons for their limitations and medical rejection [7]. Standards have been developed to define how EHR data should be structured, semantically described, and communicated. These standards include openEHR, HL7 (v2, v3, and FHIR), ASTM E1384, CEN’s TC 251, and ISO TC 215 [39]. They are often relay on medical terminologies such as SNOMED CT (SCT), LOINC, ICD, RxNorm, and UMLS. HL7 (*www.hl7.org*) is a standardization organization that provides about 90% of healthcare services [39, 44]. Recently, HL7 proposed the FHIR standard based on HTTP and RESTful services. It is a global standard, which combines the best characteristics of HL7’s v2, v3, and clinical document architecture (CDA). It provides a rich and extensible information model based on the concept of a modular *resource*. FHIR defines around 116 generic types (i.e. form templates) of interconnecting resources for all types of clinical information. It defines four paradigms for interfacing between systems, including RESTful API, documents, messages, and services [45]. FHIR is expected to achieve interoperability faster, easier, and cheaper than other standards. Leroux et al. [46] asserted that the adoption of a single format for data storage and exchange decreases the development and data exchange time, and the FHIR model has the potential to manage clinical data in its own right. FHIR received increased attention from the Harvard SMART project (*https://smarthealthit.org/*) and other public initiatives such as *Opencimi.org*. Gøeg et al. [47] asserted the priority of FHIR because it is based on web technologies, which ease implementation; in addition, FHIR is more suitable for mobile applications because it is based on a RESTfull service-oriented architecture. Using this HTTP-based paradigm, mobile problems such as short battery life are less likely to occur. Although this standard supports interoperability, an ontology can enhance semantic interoperability between different systems, especially between WBAN and EHR data [48]. “*A solid ontology-based analysis with a rigorous formal mapping for correctness*” is one of the 10 reasons why FHIR is better than other standards [49]. As a result, integrating FHIR-based EHR data with a CDSS knowledge base ontology can improve the seamless integration and interoperability of decision support features in an EHR ecosystem. No studies in the literature have discussed this issue. In addition, FHIR was modelled as an OWL 2 ontology (*http://wiki.hl7.org/index.php?title=* *RDF_for_Semantic_Interoperability*). It has not been connected to any formal top-level ontologies like BFO, and it has not been utilized in real applications yet, especially in the medical domain.

### Study objectives

In light of the above, we propose an ontology-based mobile health CDSS for type 1 diabetes monitoring and treatment. This cloud-based and comprehensive architecture allows patients to be connected with different service providers as well as different sources of medical data. The system is based on a set of standards to handle interoperability challenges. Integration of these standards is based on ontology representation and reasoning. To support transparent integration and semantic interoperability between the CDSS and distributed EHRs, this proposal is based on the most recent HL7 interoperability standard of FHIR. The SSN is utilized to integrate sensor data with historical EHR data. To unify the semantic meaning of all used terminologies and knowledge, all terms used are understood and embedded under BFO universals. We collected medical knowledge from the most recent T1D CPGs, scientific research, and official web sites [20]. CPGs are documents that collect all the available medical evidence with regard to a particular disease. They support the evidence based medicine paradigm. Knowledge of CPGs is implemented as OWL 2 axioms and SWRL rules to build and infer tailored TPs and to provide real-time monitoring for diabetics. Security and privacy issues, however, are outside the scope of this paper. Specifically, this proposal makes the following major contributions, compared with previous methods.

- We propose an interoperable, expandable, and cloud-based mobile CDSS framework for T1D management. This CDSS can remotely monitor diabetics according to their real-time WBAN metrics, and suggests adjustments in insulin dosages, exercise plans, and diet plans. The CDSS can discover critical situations, including hypoglycemia and hyperglycemia, and can suggest emergency procedures. In addition, it is able to propose actionable, evidence-based, standard, accurate, and medically complete TPs based on patient conditions and preferences collected from real-time data and historical EHR profiles.
- Effective CDSS depends mainly on the quality of its knowledge base. As a result, we propose a real, holistic, global, and extensible T1D-treatment OWL 2 ontology (FASTO) based on SHOIQ (D) description logic. This ontology is the core knowledge base of the proposed CDSS. It supports temporal reasoning about patient observations and TPs. FASTO is built using the Protégé 5.1 ontology editor.
- This CDSS suggests plans that include critical treatment components of insulin monitoring and management, lifestyle (i.e. diet and exercise), and education. To support evidence-based medicine, the TP-formulated treatment rules are extracted from the most recent standard diabetes CPGs. We employ SWRL rules to represent CPG knowledge, and we use ontology reasoners to implement the CDSS inference engine.
- We propose a method to collect and integrate all patient data from heterogeneous sources in a centralized cloud-based EHR database based on the most recent HL7 standards (i.e. FHIR). This database is used to instantiate FASTO. In addition, this database can be utilized by machine learning techniques to enrich CDSS knowledge.
- The majority of the system processes are executed in the cloud. FASTO and an ontology reasoner provide real-time *knowledge-as-a-service* to patients and physicians. As a result, the resources (i.e. memory, battery, and processor) of a patient’s mobile device will be preserved for monitoring.
- The FASTO novel knowledge model reuses several standard ontologies, including the BFO 2.0 top-level ontology, vital-sign ontology, medical terminologies, and SSN sensor ontology. To support effective and efficient data exchanges between distributed and heterogeneous system modules (i.e. CDSSs, WBANs, and EHR distributed systems), we created our proposed system based on the most recent and publicly acceptable interoperability standard of HL7 FHIR. All FASTO concepts are unified with FHIR resources. The utilized SSN concepts are implemented according to the semantics and structures of FHIR resources, and all ontology classes are modeled as subclasses of BFO universals. The data are exchanged between modules based on FHIR servers and in JSON format.
- The resulting fully-fledged FASTO ontology is transparent and independent from EHR systems’ different data formats and different sensor data standards, thanks to the FHIR standard. As a result, our CDSS is portable, offering a plug-and-play capability with any EHR ecosystem after little configurations.

The quality of the proposed CDSS framework is based totally on the design quality of FASTO. As a result and due to space restrictions, we provide an overview of the whole CDSS framework, and then focus more on the development and testing of the CDSS ontology.

## Methods

**Fig. 1:**
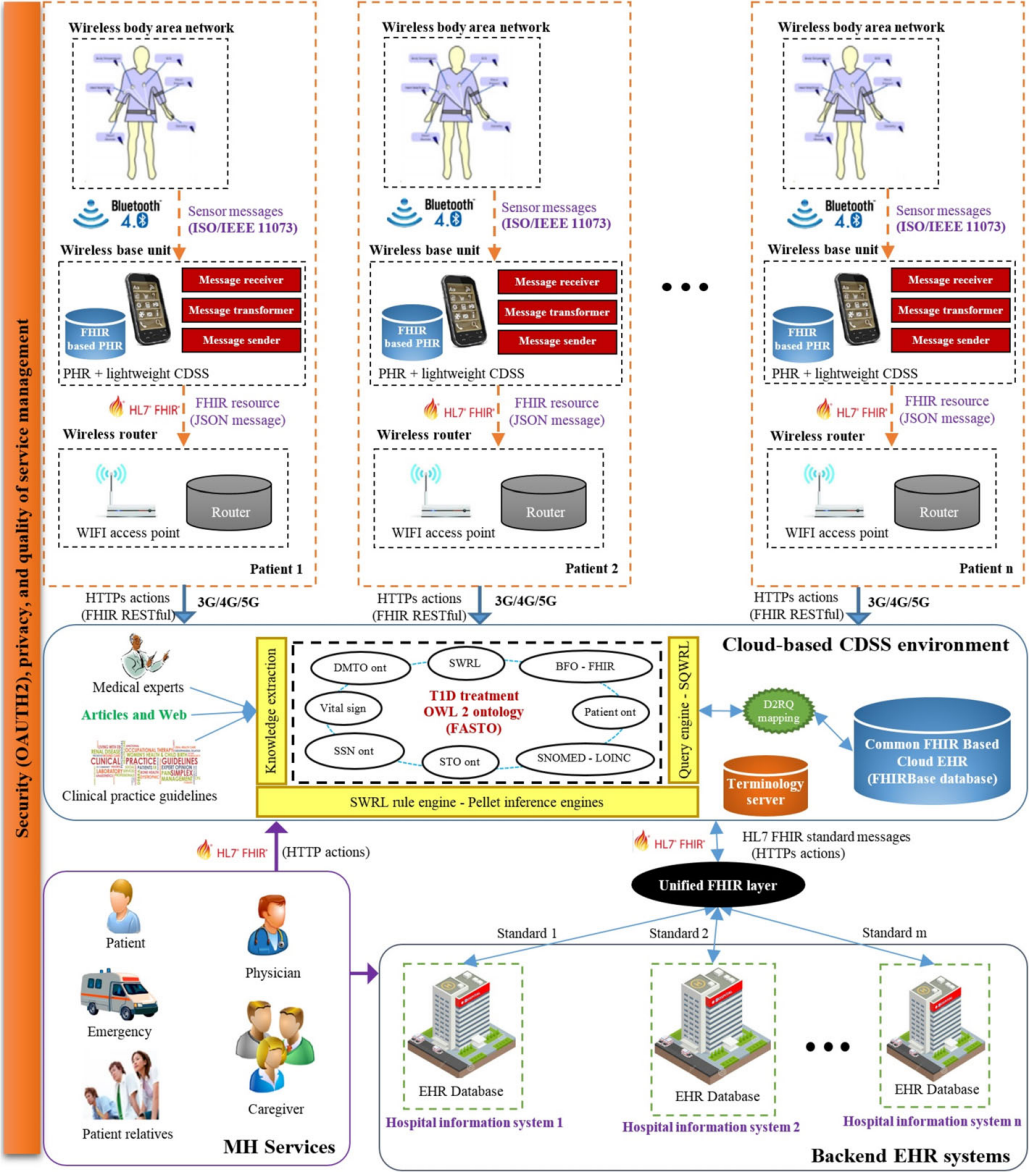
The architecture of the proposed CDSS framework at high-level of description. This is the proposed framework modules including patient module, the mobile health services module, the cloud-based CDSS module, and the backend EHR systems module

This section discusses the proposed mobile patient monitoring framework (see Fig. 1). This framework supports the continuous and mobile monitoring of T1D patients based on cloud computing solutions, which provides accessibility, extensibility, flexibility, cost savings, and deployment speed. Our framework has four main modules: the patient module, the services module, the cloud-based CDSS module, and the backend EHR systems module. Each module provides a particular set of functionalities. These modules are integrated in a standard way based on ontology and FHIR. HL7 FHIR servers are responsible for collecting data from distributed hospital information systems to be stored in a cloud-based EHR. As a result, these modules are loosely coupled. Therefore, change in one module does not alter the whole architecture. The system mainly depends on ontology semantics, standard terminologies, and HL7 FHIR to solve the major challenges of syntax and semantic interoperability.

Different from the state-of-the-art systems, we integrate low-level sensor data and EHR data with high-level ontology knowledge in a standard way to make accurate and medically acceptable decisions. Our main goal is to produce a global data model and a standard knowledge base, which decreases system development time and data transformation errors. To achieve this goal, the logical data models of all designed databases and FASTO semantics are based on the FHIR resource information model. We reviewed the emerging FHIR model definitions to identify resources appropriate for modeling of basic clinical contents (e.g. medications, care plans, observations). In a parallel process, some common data models, such as Open mHealth (*http://www.openmhealth.org*) standard schemas and clinical element models (*http://www.opencem.org*), are reviewed to build a medically complete system. Standard medical terminologies are used for encoding the used terms. Numerical values are encoded with standard units of measurement. The resulting system supports seamless and transparent interoperability between a CDSS and an EHR. In the following sections, we will discuss each module in detail.

## Patient and Mobile health services modules

The patient module is responsible for collecting a patient’s WBAN sensed data and dispatching them for further processing. It is based on a set of heterogeneous off-the-shelf biosensors that monitor and communicate physiological parameters of the individual, including physical activity, blood glucose level, and vital signs. These sensing devices have interface (APIs) that allow access to the collected data. The time-stamped, streamed data are automatically transmitted to a wireless base unit (WBU) (i.e. a mobile phone) via Bluetooth for further preprocessing and formatting. To achieve end-to-end semantic interoperability, the ISO/IEEE 11073 family of standards is used as an open standard for message formatting and as communication protocol between the WBAN and the WBU. The messages are built by applying ISO/IEEE 11073-104zz device specifications to the observed data according to sensor type (e.g. blood pressure, weighting scale, glucose level, heart rate). Furthermore, real-time data can be manually entered by the patient, like the intent to eat *x* grams of carbohydrates (carbs) for every meal, the height, the intent to play exercises, etc. These data are sent to the cloud-based EHR database based on specific criteria (e.g. during a specific event, at a specific time, or manually).

The mobile phone acts as an aggregation manager, where data are collected, preprocessed, standardized, and stored in a personal health record (PHR). The PHR is implemented as a SQLite RDB (*http://www.sqlite.org*). Raw, real time sensor data have no semantics, which cannot be used collaboratively with hospital EHR data. As a result, the received sensor data based on ISO/IEEE 11073 are mapped or converted into FHIR resource formats and collected in the PHR. Suitable resources for a PHR include *observation* (for sensor data, amount of carbs, height, BMI, and exercises), *patient* (for age, name, address, gender, contacts, etc.), *device* (for sensor devices), and *carePlan* (for current care plan). As a result, the WBU has three functions executed sequentially. The first is a message receiver function that is responsible for collecting data from the WBAN. The second is a message transformer function, which converts ISO/IEEE 11073 messages into FHIR resources using JSON format and stores them in PHR. Interoperability between FHIR and IEEE 11073 is well established. The third function is a message sender, which sends the patient’s sensed and non-sensed data from the WBU to the cloud as inter-linked JSON documents. To easily map PHR data to cloud EHR data, all system databases are designed based on FHIR resources.

Selected resources are formatted as JSON RESTful messages because they are widely used and have a relatively small overall data size. Resources can be posted individually, or a *Bundle* resource could be used as a container for a collection of inter-linked resources and transmit them at one time. These messages are sent via WIFI wireless connection to the nearest access point, and then via 3G/4G/5G to be integrated into a centralized cloud-based EHR. The collected cloud data are utilized as the ABOX of FASTO. The list of the system’s services is implemented in the services module.

## Cloud-based CDSS module

This module is the core of the proposed architecture. It provides *knowledge as a service* approach to deal with the heterogeneity, distribution, and scalability of medical data. It is responsible to gather patient data from different sources (sensors and EHRs) and standardize, process, analyze, and visualize them in accordance with knowledge extracted from CPGs [20]. This module has two main components, namely CDSS engine and FHIR-based EHR database.

### The CDSS engine

The CDSS engine is based on ontology and its reasoner capabilities. The ontology provides a formal, sharable, reusable, machine readable, interpretable, structured, extensible, and semantically intelligent representation of knowledge. The input to the reasoner is the complete patient profile of real-time continuously sensed data plus historical EHR data. The output is the continuous monitoring of the patient by providing real-time blood glucose monitoring and complete T1D TPs. The ontology reasoning process personalizes the available medical knowledge according to the patient’s individual conditions. Accordingly, it provides a customized action plan suitable for the specific patient. Note that the ontology contains only the data required to make a decision at one concrete moment, but the complete patient medical record remains in the cloud-based EHR.

**Fig. 2:**
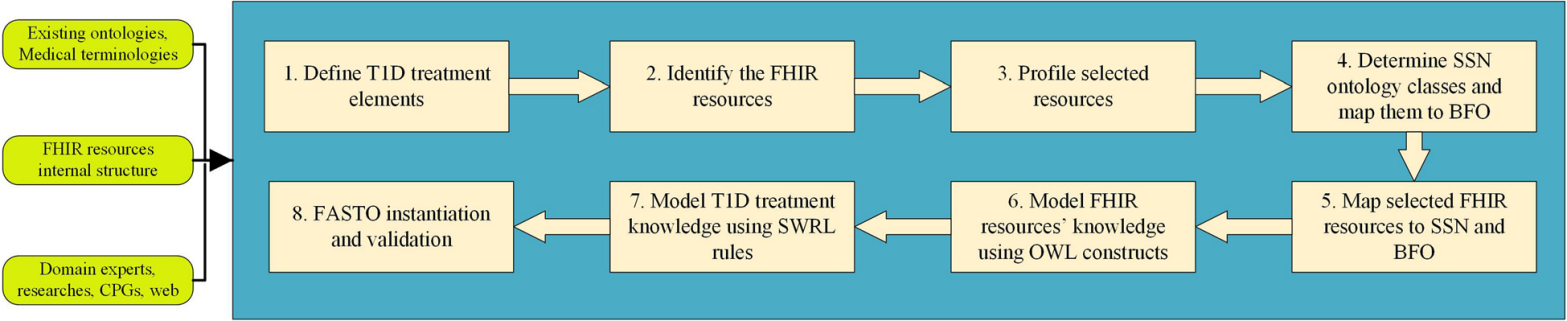
The FASTO construction steps. These are eight sequential steps for building the proposed FASTO ontology from defining diabetes treatment elements up to instantiation and validation

In this section, we describe the detailed process for creating the FHIR and SSN-based mobile ontology for T1D treatment (i.e. FASTO). The main steps are depicted in Fig. 2. We depend on many sources to create a medically accurate and complete ontology. These sources include existing ontologies and medical terminologies, domain expert knowledge, the most recent research, and official web sites. In addition, we study the most recent CPGs to extract treatment knowledge and convert it into SWRL rules and ontology axioms. We pay a close attention to interoperability in the construction process to support the creation of a sharable, reusable, and publically acceptable knowledge base. The collected data are aggregated from heterogeneous sources encoded with heterogeneous medical terminologies and designed by heterogeneous data models. As a result, all ontology knowledge is standardized according to the HL7 FHIR standard. In addition, all used terms are based on standard terminologies, which deeply support the enrichment of the ontology as well.

FASTO is designed in modules to support extensibility and reusability. Each module handles a specific dimension of the modeling process. Some modules are imported from standard ontologies, and other modules are built from scratch to add T1D treatment knowledge. We employed a top-down strategy to define the proposed ontology, which is based on BFO 2.0 as the upper-level foundational ontology to unify the meanings of used terminology. BFO is a domain-independent and comprehensive ontology; it has rigorous conceptualization, and hence, supports reusability, modularity, extensibility, and interoperability. First, we defined the top-level classes in our ontology, and then, we semantically aligned them with BFO universals. Ontology alignment can be defined as a set of correspondences or relations (e.g. equivalence ≡, subsumption ⊑, and disjointness ⊥) between two ontologies [50]. Next, we deeply modeled the semantics for each of these classes as a sub-ontology designed for a specific purpose. We based this mainly on reusing existing standard ontologies when possible.

We collected these ontologies from BioPortal (e.g. diabetes mellitus diagnosis ontology [DDO], DMTO, RxNorm, SCT, LOINC, and BFO) and from the W3C (e.g. the SSN and the FHIR ontology: *http://w3c.github.io/hcls-fhir-rdf/spec/ontology.html*). The treatment decision is based on two main sources of data, namely WBANs and EHR systems. Some classes and properties were added to integrate the sensed data with data extracted from hospital EHR systems. In addition, treatment knowledge extracted from CPGs (*www.nice.org.uk*, *www.guideline.gov**,* the American diabetes association: *www.diabetes.org*, and Canadian diabetes: *www.diabetes.ca*), professional web sites (e.g. *www.medscape.com*, *www.drugs.com*, *www.medicinenet.com*, *www.uptodate.com*, *www.fda.gov**,* and *http://sideeffects.embl.de/*), and scientific research [11, 51–56] is manually modeled as axioms and rules.

To automate the ontology population process and patient data aggregation, it is urgent to maintain bi-directional and one-to-one mapping between FHIR resource messages, cloud database constructs, and FASTO constructs. In other words, resources are “*losslessly round-trippable*” between different formats. Our cloud database is designed based on an FHIR resource schema to easily transform resource instances to RBD instances. Every FASTO class is manually translated into a specific FHIR RDF resource. Please note that FHIR resources are modeled with different formats, such as JSON, XML, and RDF. Constraints and data types of FHIR resources are mapped to OWL 2 constructs, axioms, data types, and SWRL rules by using the Protégé 5.1 ontology editor (*https://protege.stanford.edu*). Extensions to and customizations of selected resources are implemented as needed for the T1D domain.

*First*, we formally defined the data elements that are required to represent T1D treatment. This step was informed by our previous work for type 2 diabetes diagnosis (i.e. DDO) and treatment (i.e. DMTO), and SCT modeling (SCTO). *Second*, to standardize this knowledge based on the FHIR resource format, we manually browsed and analyzed these resources to identify needed resources. Note that we depend on the FHIR standard for trial use (STU 3) specification. *Third*, we profiled selected resources to customize them according to our CDSS requirements. *Fourth*, we determined the SSN ontology classes required to represent our domain and mapped these classes to BFO universals. *Fifth*, we mapped some of the selected resources to the SSN ontology classes and modeled the rest of the resources as subclasses of BFO universals. These mappings depend on a deep understanding of the used ontologies and lengthy discussions with experts, such as BFO authors. *Sixth*, elements and constraints of the resources were modeled as ontology classes, properties, axioms, and SWRL rules. *Seventh*, we added T1D treatment knowledge in the form of relations, properties, axioms, and SWRL rules. FASTO follows the principles of ontology development established by the OBO Foundry (*http://www.obofoundry.org*).

#### Define T1D treatment elements

We tried to minimize manual data input from patients. As a result, critical patient data are collected automatically from three main sources, thanks to the FHIR standard interface. The first source is the patient WBAN, which includes sensors’ real-time vital signs, glucose levels, and weights. The second source is the patient profile collected from distributed EHR systems. These data include the patient’s demographics (weight, age, gender, smoking status, and height), BMI, preferences, symptoms, lab tests (e.g. HbA1c, LDL, etc.), allergies, complications, previous plans, family history, and medications. The third source is the data manually sent by the patient as real-time non-sensed data, such as an intent to eat carbohydrates, an intent to play exercises, and other emergency consultation data. Another type of knowledge modeled in the ontology is the TP components, which include the care team, the care plan, treatment goals, food and dietary meals, exercises, insulin, and education. The ontology includes additional semantic knowledge regarding units of measurement, interactions (drug-drug, drug-disease, and drug-food), allergies, drug side effects, etc.

#### Identify FHIR resources and profiling

Table 1 describes medical data elements representing T1D treatment and their mappings to a set of interlinked FHIR resources. A custom TP suggests actions to handle specific conditions for a specific patient. Most features required to build custom treatment plans are imported from an FHIR model, including *who* (e.g. patient, physician, care team, or relative), *why* (e.g. goals and risks), *what* (e.g. medications, medication allergies, vital signs, lab tests, diet, and exercise), and *where* (e.g. location). We utilize about 23 resources to build complete plans. Each resource has a unique ID, connected based on patient identifiers. Profiling is a required step because FHIR is a generic model. We added and/or removed some fields in some resources; in addition, we changed some field constraints. For example, the *category* field from *CarePlan* is removed because we only consider CarePlan.category = 698,360,004|Diabetes self management plan. We depend on the FHIR *vital sign*, *BMI*, and *blood glucose* profiling. To preserve the monotonicity of FASTO, we considered the final state for all resources (e.g. Observation.status = “final” and Condition.verificationStatus = “confirmed”). Background knowledge such as *foods* and *interactions* (e.g. *food-drug*, *food-disease*, *drug-drug*, and *drug-disease*), and drug side effects are modeled away from FHIR but are used in a standard way with resources such as *NutritionOrder* and *Detectedissue*, respectively*.* Remote-monitoring resources (e.g. observation) are mapped to SSN classes, and all classes are mapped to BFO universals. All references are modeled as object properties.

**Table 1:** T1D treatment essential data elements

TP data element	HL7 FHIR resource	Description	SSN class	SSN Mapping	Cloud database table	BFO universal
Patient + demographic	Patient	Person who plays the patient role (e.g. age, address, gender)	–	–	Patient	⊑BFO: BFO_0000023
Physician	Practitioner	Person who plays the role of physician, nutritionist, etc.	–	–	Practitioner	⊑BFO: BFO_0000023
Relative	Related person	Person who plays the patient’s family member role	–	–	Related person	⊑BFO: BFO_0000023
Vital sign	Observation	Vital signs such as blood pressure, temperature	observationValue	Exact	Observation	⊑BFO: OBI_0000027
Blood glucose level	Observation	Patient blood glucose level from sensor	observationValue	Exact	Observation	⊑BFO: OBI_0000027
Lab test result	Observation	Lab test result (e.g. HbA1c, LDL, and RPG)	–		Observation	⊑BFO: OBI_0000027
Physical examination	Observation	Such as height, weight, BMI, and level of activity	observationValue	Exact	Observation	⊑BFO: OBI_0000027
Social history	Observation	Patient medical history (e.g. smoking history, alcohol intake)	–	–	Observation	⊑BFO: OBI_0000027
Patient symptom	Condition	Persistent patient symptoms that need long term management	–	–	Condition	⊑BFO: BFO_0000019
Complication	Condition	Pregnancy and current and previous diseases or diagnoses	–	–	Condition	⊑BFO: BFO_0000019
Body site of sensor	BodySite	Describe sensor place	platform	Partial match	BodySite	⊑BFO: BFO_0000006
Adverse even	AdverseEvent	Events occur during the course of patient medical care	–	–	AdverseEvent	⊑BFO: BFO_0000016
Patient allergy	AllergyIntolerance	Description of patient allergies	–	–	AllergyIntolerance	⊑BFO: BFO_0000016
Patient location	Location	Location of the patient	–	Exact	Location	⊑BFO: BFO_0000006
Family history	FamilyMemberHistory	Medical history of patient’s family members	–	–	FamilyMemberHistory	⊑BFO: BFO_0000182
Treatment plan	CarePlan	Define patient’s future, current, or past custom care plan	–	–	CarePlan	⊑BFO: OBI_0000011
Goal	Goal	Define the medical goal of a care plan	–	–	Goal	⊑BFO: BFO_0000019
Diet	NutritionOrder	Describe ordered diet	–	–	NutritionOrder	⊑BFO: BFO_0000019
Drug	Medication	Define a drug such as insulin	–	–	Medication	⊑BFO: BFO_0000040
Plan medication	MedicationRequest	Medication prescription for patient	–	–	MedicationRequest	⊑BFO: IAO_0000030
Medication dosage	Dosage	Dosage instruction information	–	–	Dosage	⊑BFO: BFO_0000019
Taken medications	MedicationStatement	Patient’s current medications list	–	–	MedicationStatement	⊑BFO: IAO_0000030
WBAN sensors	Device	Describes WBAN components (e.g. sensor)	sensing device	Exact	Device	⊑BFO: BFO_0000040
Patient-physician	Encounter	Interaction between a patient and healthcare provider	–	–	Encounter	⊑BFO: OBI_0000011
Patient-physician	EpisodeOfCare	Track provider for ongoing care of the patient	–	–	EpisodeOfCare	⊑BFO: OBI_0000011
Care team	CareTeam	Group of practitioners responsible for patient monitoring	–	–	CareTeam	⊑BFO: BFO_0000023
Exercise	Procedure	A procedure done by patient as a part of treatment plan	–	–	Procedure	⊑BFO: BFO_0000019
UoM	Element	Units of measurement	–	–	–	⊑BFO: IAO_0000030
Education	Procedure	Patient education as a part of the treatment plan	–	–	Procedure	⊑BFO: OBI_0000011

For extensibility reasons, all FHIR primitive and complex data types are implemented as OWL 2 classes with appropriate cardinality restrictions. All primitive data types (e.G. *integer*, data, URI, etc.) are defined as subclasses of the fhir:primitiveDatatype class, which is defined as: fhir:primitiveDatatype ⊑ {(fhir:element ⊑ ‘BFO:information content entity’)⊓(fhir:hasValue max 1 rdfs:literal)}. We implemented 16 primitive types, and each one of them is mapped to one or more XSD types by putting constraints to literal values. Complex data types are modeled with a specific name for each property. We implemented 14 complex types. For example, the fhir:timing class is defined as:

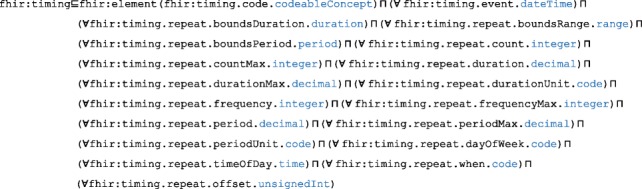



Units of measurement (UoMs) are implemented in SCT under the (282372007) concept, and UO OWL 2 ontology (*https://bioportal.bioontology.org/ontologies/UO*) provides another design method. However, we depend on the standard selected by HL7, i.e. the unified code for units of measurement (UCUM: *http://unitsofmeasure.org/ucum.html*). The unitOfMeasure class is defined as ⊑(∃hasUoMCode.xsd:string)⊓ ∃hasUoMDisplay.xsd:string), where both display and code are imported from UCUM.

#### Determine SSN classes and map them to BFO

**Fig. 3:**
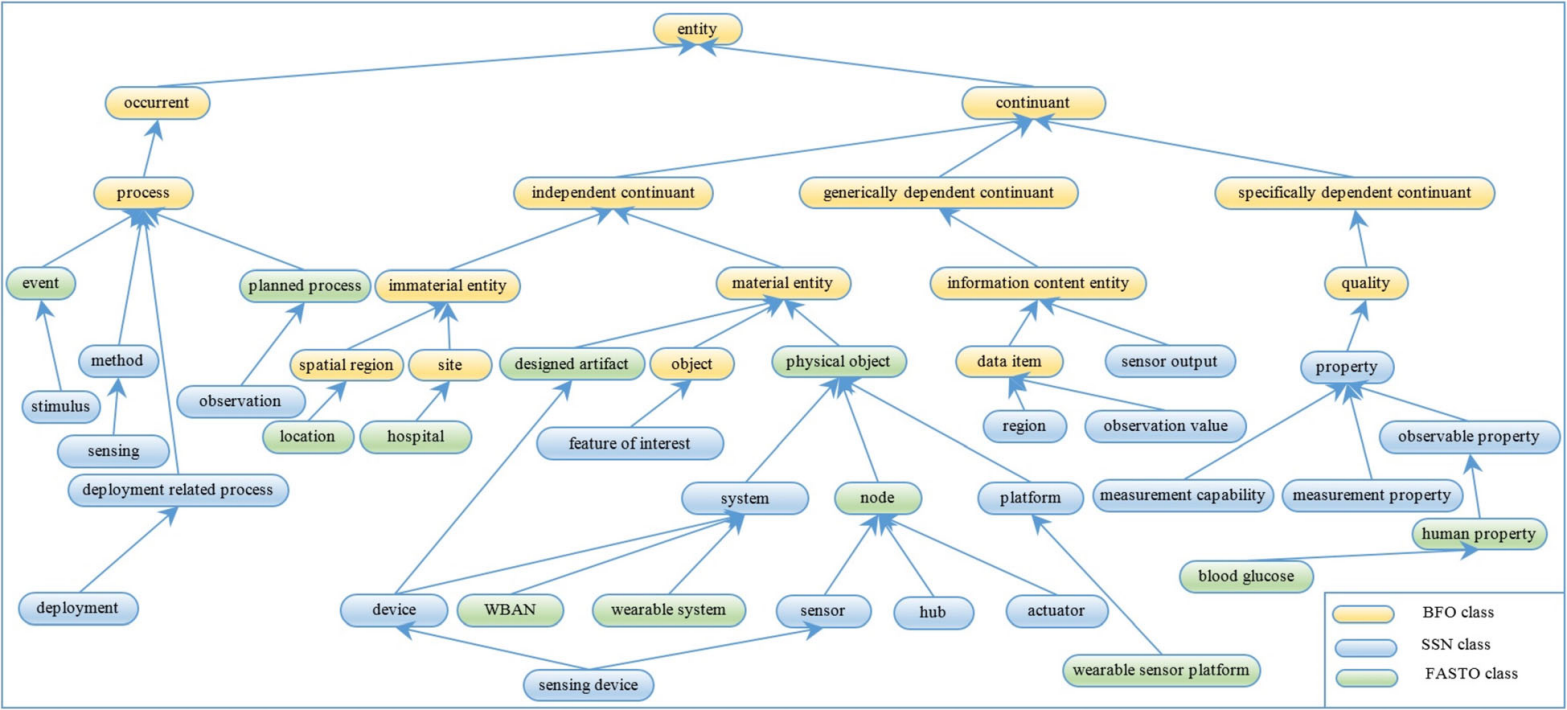
The basic SSN concepts and BFO universals associations. These are the main mapping classes between SSN ontology and the upper level ontology of BFO

This knowledge is related to modeling WBAN components and the biomedical parameters they measure. Sensors in a patient’s WBAN have heterogeneous types and data formats. Recently, there has been rising interest in ontologies to improve integration of and communication with sensor networks. The main idea is to annotate sensor data with spatial, temporal, and thematic semantic metadata to achieve interoperability and provide contextual information. The ontology supports the connection of sensor data and other sources of data. In addition, it provides semantic reasoning capabilities. The SSN (*http://purl.oclc.org/NET/ssnx/ssn*) is an OWL 2 ontology created by W3C [57]. It is a general sensor ontology based on the DOLCE (*http://www.loa.istc.cnr.it/old/DOLCE.html*) upper-level ontology to support reuse and semantic interoperability. The SSN leverages the Sensor Web Enablement (SWE) standard. In this paper, we extend the SSN to be used for T1D treatment. On the other hand, FASTO is based on BFO as the top-level ontology in order to support semantic interoperability between distributed systems. BFO (*http://basic-formal-ontology.org*) is a realistic ontology, and many medical ontologies are based on it [9, 10]. In addition, the ontology for general medical science (*https://bioportal.bioontology.org/ontologies/OGMS*), which is the most high-level ontology in the medical domain, is based on BFO. The semantic alignment of SSN top-level concepts with BFO universals is based on detailed discussions with the authors of BFO and on existing research [58], see Fig. 3. This alignment is described in the following description logic axioms, where *ssn*, *bfo*, *fasto*, and *sban* namespaces are used.

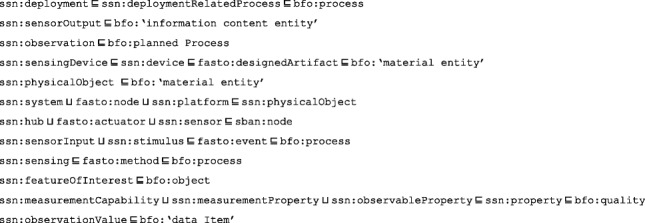



We used prefixes to indicate the sources of knowledge (e.g. *fasto* for FASTO). The previous subsumption axioms are extended by other anonymous classes for each class. For example, ssn:sensingDevice is defined as follows:

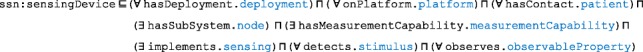



The SSN ontology has 10 main modules. We will not import all of them into the current version of FASTO. We concentrate on the sensors, their sensed objects, and resulting observations. For example, no classes related to the physical characteristics of the sensors and the WBAN are imported, such as batteryLifetime, Latency, Manufacture, MaintenanceSchedule, Security, Processor, OperatingPowerRange, ResponseTime, SystemLiveTime, Frequency, Resolution, DetectionLimit, and Sensitivity. Most of these classes are in the MeasuringCapability and OperatingRestriction modules. The other classes and their related properties are imported with the same semantics. A total of 10 classes, 26 object properties, and 5 data properties are imported from the SSN ontology. The data are collected in the ontology based on the HL7 FHIR standard format and vital sign ontology (*http://purl.bioontology.org/ontology/VSO*) terminology. All patient vital sign classes are subclasses of OGMS_0000029 or the vital sign class; in addition, these classes and bloodGlucoseLevel are subsumed by featureOfInterest. The process of transforming raw sensor data into JSON format is done on the WBU (i.e. the mobile phone). Collected data from WBANs has temporal and location dimensions. The temporal dimension is modeled by the SWRL TO ontology (*http://swrl.stanford.edu/ontologies/built-ins/3.3/temporal.owl*). The SWRL TO ontology is lighter than the W3C Time ontology (*https://**www.w3.org/TR/owl-time*) and, at the same time, is sufficient. It has four main classes (STO:validTime, STO:granularity, STO:validInstant, and STO:validPeriod), two object properties (STO:hasGranularity and STO:hasValidTime), and three data properties (STO:hasFinishTime, STO:hasStartTime, and STO:hasTime). In addition, it provides some temporal capabilities using SWRL buildins. The SSN is extended by some knowledge from the SmartBAN ontology (*https://www.etsi.org/technologies-clusters/technologies/smart-body-area-networks*) including fasto: Node⊑ssn:physicalObject and fasto: WBAN⊑ssn:system⊑ssn:physicalObject classes, and SBAN:hasContact object property. We defined two new classes of fasto:wearableSystem and fasto:wearableSensorPlatform to extend the definition of ssn:system and ssn:platform, respectively, for sensors located on the patient’s body, respectively.

In addition, a new object property, fasto:placedOn, is defined for the axiom of (wearableSensorPlatform⊑∀ placedOn.humanBodyPart), and body parts can be defined according to the foundational model of anatomy (*https://bioportal.bioontology.org/ontologies/FMA*). According to the recent CPGs, blood glucose, not HbA1c, must be used to monitor T1D patients [20]. In addition, to build an accurate and continuing TP, a complete medical evaluation should be conducted based on a complete patient profile. Raw data collected from different sensors cannot easily work together owing to the lack of semantic interoperability. SSN converts these data to semantic data, but integrating sensors data with EHR data is another challenge. To handle this challenge, collected knowledge needs to be standardized to achieve semantic interoperability among its sources. As a result, we will extend the previously modeled knowledge by FHIR resources.

#### Map resources to SSN and BFO

According to Table 1, all selected resources are modeled as subclasses or equivalent classes to either SSN classes or BFO universals. This mapping is based on a deep study of these ontologies and long discussions with BFO authors. The resulting mapping helps to unify the meaning of ontology knowledge, which improves the portability, shareability, reusability, and customizability of the resulting knowledge.

#### Model resources knowledge with OWL constructs

In this step, we extend the semantics of SSN in a standard way based on FHIR semantics. Medical, location, and temporal concepts are linked to SSN knowledge. In addition, we model the FHIR semantics for the other non-sensor EHR data and relate this knowledge to SSN concepts. The implementation of resource knowledge is achieved according to the official resource schemas. HL7 FHIR provides the basic forms for each resource. To be applicable in our domain, all resources are profiled according to our domain. All classes in Table 1 were modeled by first profiling the FHIR resources and then extending these resources with some knowledge required for our problem domain. Resources are modeled as FASTO classes, and each resource element is modeled as an object property using the pattern [*ResourceName.ElementName*]. The following are the semantics for some classes based on FHIR resources described in description logic syntax. We extended the medication resource as follows:

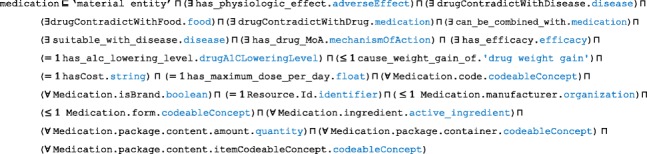



In addition, the insulin class is subsumed by the medication class as follows:

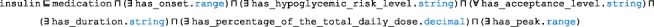



The class person≡{patient⊔practitioner⊔relative} is defined as follows:

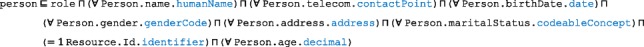



The patient class is extended to capture the sensor and WBAN knowledge as follows.

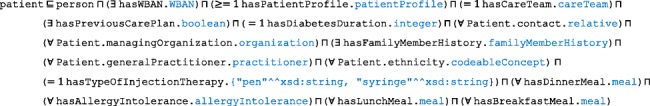



The observation resource is used to model all types of observations including *sensor observations* (e.g. vital signs and blood glucose level) and *non-sensor observations* (e.g. lab tests and physical examinations results). All temporary patient characteristics are collected in the patientProfile class including observations (i.e. observationValue), complications (i.e. condition), symptoms (i.e. condition), adverse events (i.e. adverseEvent), medications (i.e. medicationStatement), encounters (i.e. encounter), food (i.e. food), care plans (i.e. carePlan), etc.

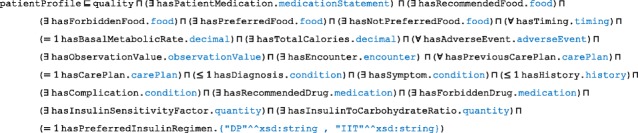



We preserve the flexibility of FASTO, where the same piece of knowledge can be accessed in different ways. For example, patient complications can be collected according to encounters or from the profile. To achieve interoperability and completeness, the SSN’s observationValue class was extended according to the Observation FHIR resource, which defines its observed quantity, coding, and UoM as follows:

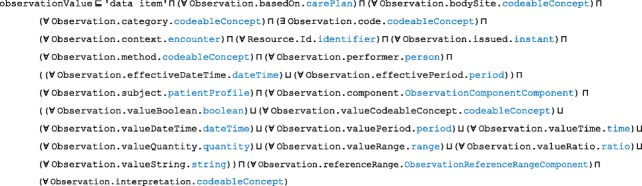



**Fig. 4:**
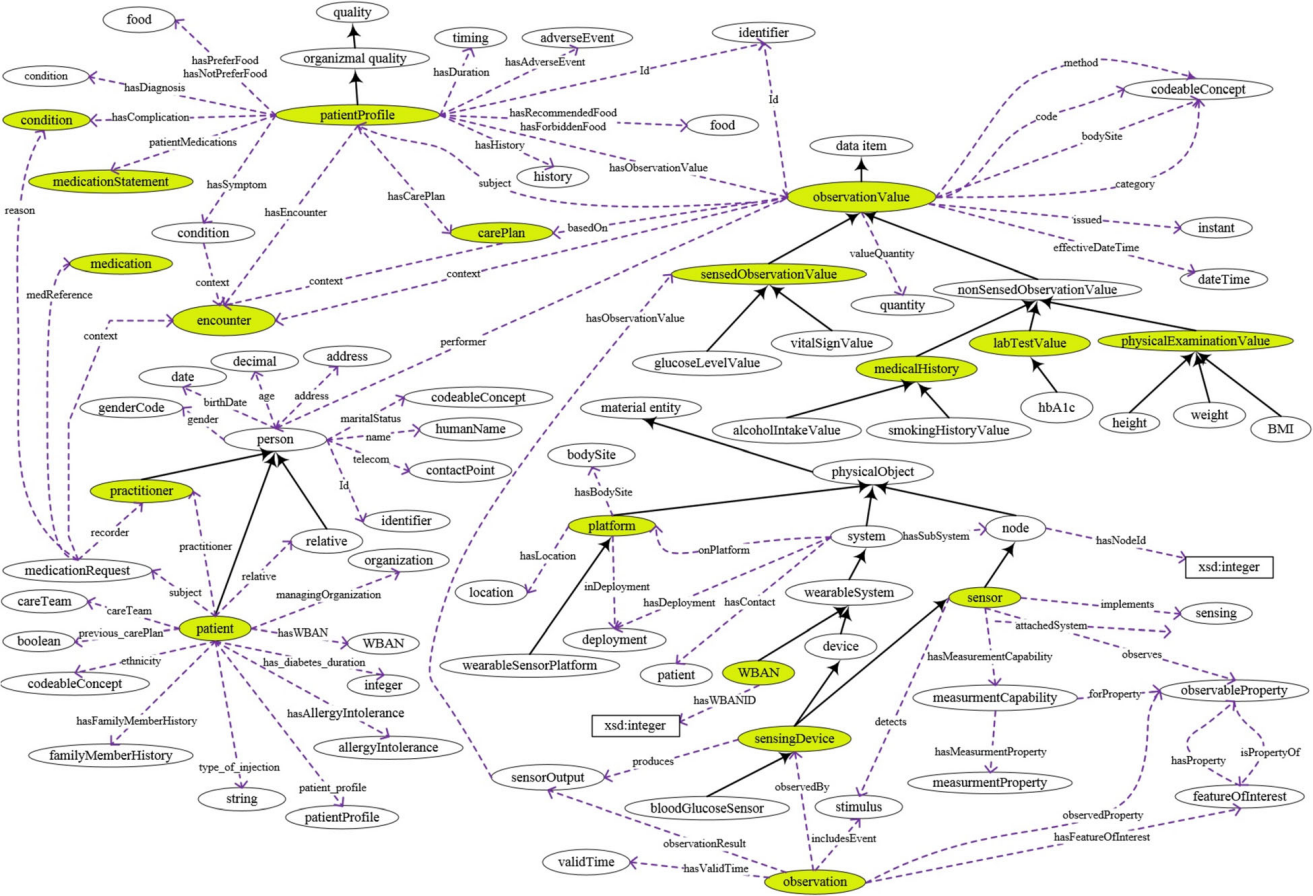
The observationValue class in its context. These are the main classes related to the observationValue class including the patient, sensor, and patientProfile classes

Figure 4 depicts the observationValue class in its context with sensor, WBAN, patient profile, and carePlan classes. Many classes, and many class properties, were removed to keep the figure simple. The Condition class is used to model patient’s pregnancy, current or historical symptoms, diagnoses, and complications based on the Condition.category object property. We imported the possible diabetes symptoms from our DDO, and possible diagnosis and complications from our DMTO. The condition class is used to define specific conditions for specific patient to be considered in TP instantiation.

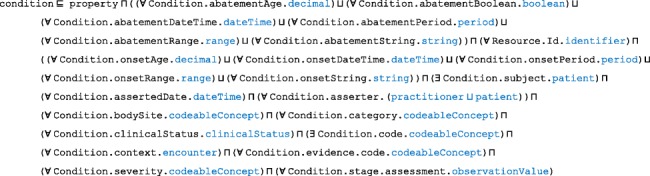



FHIR has two classes to define medications in general: medication class and the patient’s specific medications (medicationStatement). However, it has no equivalent logic for diseases. It has the condition class for patient-specific conditions, but there is no resource for modeling diseases in general. For each disease, this class is critical in order to define its code and contradicted drugs, foods, and exercises. We introduced a disease class as follows:

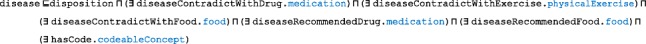



Standard medication adverse effects are imported from the ontology of adverse events (OAE: *http://purl.bioontology.org/ontology/OAE*) ontology, and every medication has its list of adverse effects. The binding of a patient with specific adverse events, (i.e. taking an incorrect drug or an incorrect dose of a drug) is defined by the adverseEvent class. Patient context, including current conditions or treatments, is essential in establishing a cause-and-effect relationship for an adverse event. As a result, we relate adverseEvent and patient profile classes with the hasAdverseEvent object property. In addition, the patient’s allergy and intolerance to foods and other substances is modeled in the allergyIntolerance class. Because allergies may not depend on the context as adverse events, this class is directly related to the patient class. The MedicationRequest class is used to prescribe insulin for the patient, and medicationStatement is used to collect the medication history of the patient.

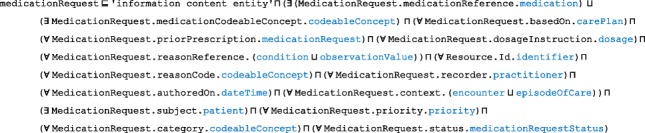



Treatment plan modeling is one of the main targets of our ontology. The carePlan class collects the semantics of the TP. Every care plan should have medication, diet, exercise, and education activities. In addition, real-time insulin dosage consultations according to changes in carbohydrates consumption and exercises are modeled.

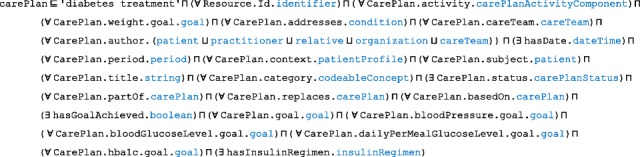



We extended carePlan to define the type of insulin regimen by using the *hasInsulinRegimen* object property. The insulinRegimen ≡{FixedRegimen⊔intensiveInsulinTherapy} class is defined as follows:

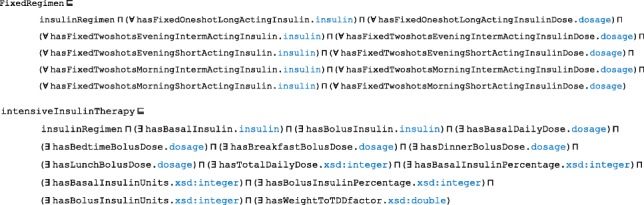



In addition, carePlan has six specific, measurable, achievable, realistic, time-oriented (SMART) goals of blood glucose goal, daily per-meal glucose goal, blood pressure goal, HbA1c goal, weight goal, and other customizable goal.

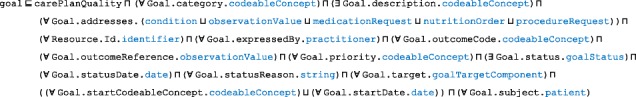



**Fig. 5:**
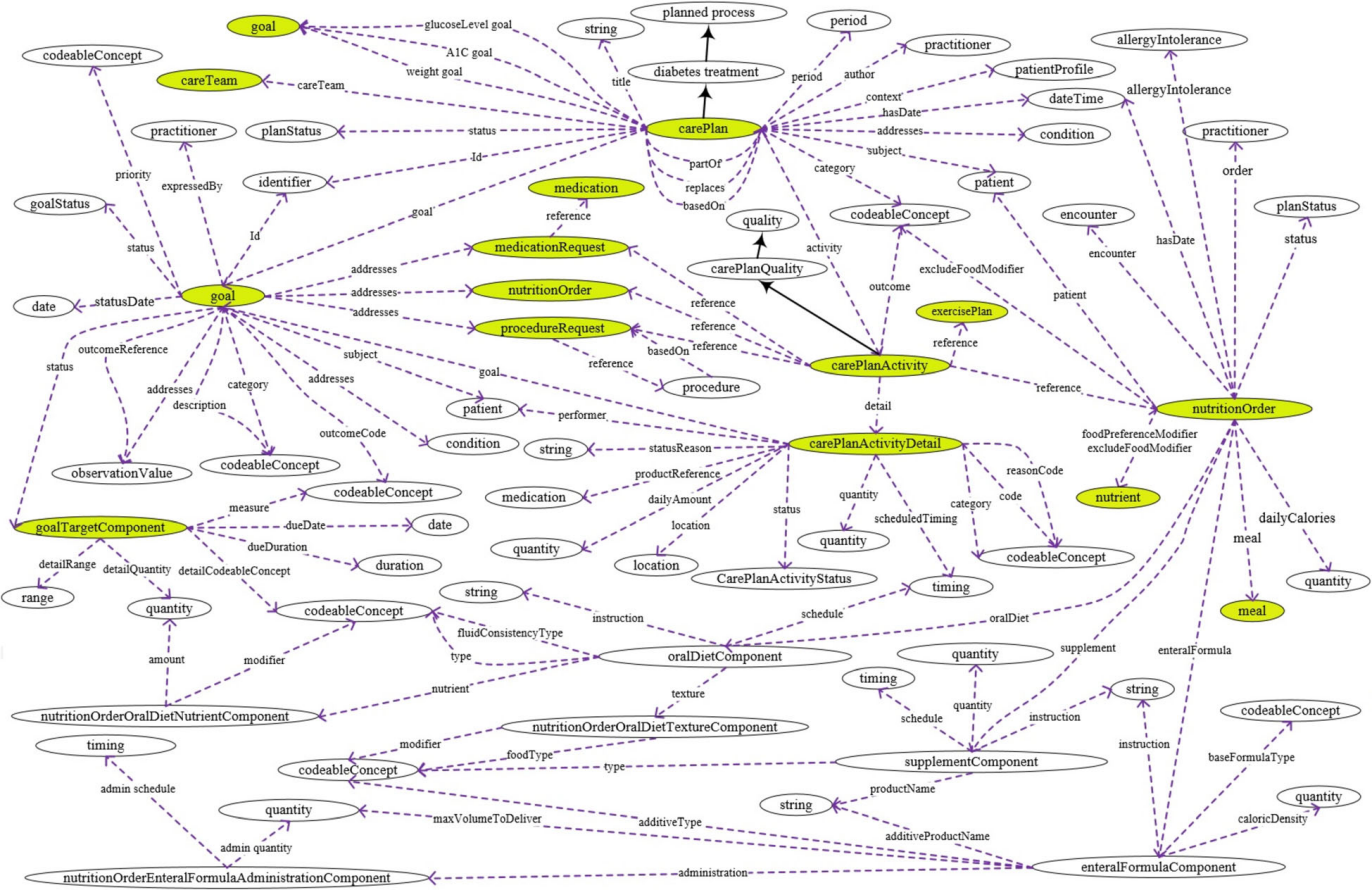
The carePlan class in its context. These are the main classes related to the carePlan class including the goal, and carePlanActivity classes

The carePlanActivityComponent class defines the long-term parts of the plan in the form of activities by using medicationRequest for insulin, nutritionOrder for diet, and procedureRequest for education, as illustrated in Fig. 5. For real-time adjustment of insulin dosage and carb grams, the patient class has three properties linked with the breakfast, lunch, and dinner classes. These classes are subclasses of the meal class, which is defined as (∃hasTotalCarbsInGrams.xsd:integer)⊓(∃hasFood.food) ⊓(∃hasValidTime.validInstant)⊓(∃hasCorrectionInsulinUnits.xsd:integer)⊓(∃ hasCarbsInsulinUnits.xsd:integer)⊓(∃hasTotalInsulin.xsd:integer). The patient monitoring process depends on data collected from hospitals. As a result, the *Organization* resource is required. The proposed system suggests TPs for physicians to approve, and the final decision is from physicians. There must be a specific party responsible for the monitoring process. As a result, the *Practitioner*, *CareTeam*, and *Relative* resources are used. To organize the monitoring process, *Encounter* and *EpisodeOfCare* are utilized. Each of these resources was designed as an OWL 2 class with suitable properties. For space restrictions, readers can find their DL definitions in the ontology.

#### Model T1D treatment knowledge with FHIR resources

**Fig. 6:**
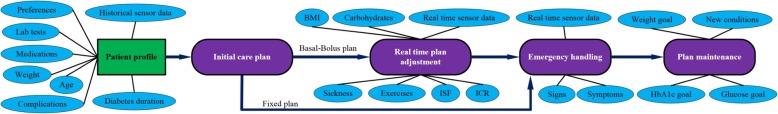
Diabetes treatment main tasks. These are the main components of diabetes carePlan including initial care plan, real time monitoring, emergency handling, and plan maintenance

The resulting FASTO ontology is rich enough to define any type of TP for diabetics. In this section, we extensively define T1D treatment knowledge according to available CPGs, expert knowledge, and online resources [11, 51–56]. Creating a TP for T1D patients is a complex process because it requires the customization of many parameters. A TP has three main sub-plans: *medication* for insulin prescription, *lifestyle* for diet and exercise definitions, and *education* for defining custom learning topics. We simulate the operation of *β* cells. These cells sense the BG level, analyze the collected data, and deliver insulin accordingly. The WBAN senses the patient’s vital signs; the ontology collects these data, combines them with the EHR profile, and makes a semantic analysis; finally, a customized plan is given to the patient with details on insulin management, lifestyle, and education. This knowledge is implemented as SWRL rules and OWL 2 axioms. FASTO manages three main situations, as shown in Fig. 6.

*The first situation* is to create long-term TPs according to the entire patient profile, previous TPs, and temporal abstraction of sensor data (if any). All of the cloud-based EHR data are instantiated in the ontology as standard FHIR resources. The *second situation* is real-time patient guidance to adjust insulin doses, carb amounts, and exercises according to dynamic patient needs and sensor readings. This step is based on real-time observations collected from the WBAN plus some calculated factors, such as insulin sensitivity factor (ISF) and insulin-to-carbs ratio (ICR) [59]. *The third situation* is handling emergencies, where the patient in danger of hypoglycemia (blood glucose <3.6 mmol/L) or hyperglycemia (blood glucose >9.0 mmol/L). These situations are kept away from long-term TPs because they require special procedures for diagnoses and handling. In this version of FASTO, we will implement the first two situations.

##### Initial care plan construction

This plan tries to manage the balance between things that increase BG (e.g. food, illness, emotion, etc.) and things that decrease BG (e.g. insulin, exercise, diet, etc.)

***Insulin prescription*** This step determines the types of insulin, the number of units, and the frequency needed to control patient glucose. Insulin is a hormone. If administered by mouth, it is digested like other proteins. Therefore, injection is the primary method of administration. It mainly depends on patient weight in order to determine the starting dose or total daily dose (TDD). Age, diabetes duration, other complications, and other medications are also considered. There are only two main regimens, i.e. intensive insulin therapy (IIT) and daily fixed (DF). IIT (basal-bolus, prandial, or multiple daily injections) is the most popular and flexible regimen. However, it is sometimes not suitable, especially for children, because it is more complex. IIT is based on selecting one basal insulin (e.g. 50% of TDD) that works as a background long-acting or intermediate-acting insulin such as *Detemir* or *Lantus*. Based on the patient’s choice, the basal dose can be taken as one shot at bedtime or divided into two (one in the morning and one in the evening). In addition, IIT selects one rapid-acting insulin (bolus or pre-meal), such as *Aspart* or *Novolog*, to be used to cover food. The other 50% of the TDD is divided into three injections per day (e.g. 15 min before each of the three meals). Measuring blood glucose before meals is critical. If it is lower or greater than the target BG, then the meal’s predefined dosage must change accordingly. If the patient skips a meal then he/she must skip the bolus dose. On the other hand, the DF regimen is easier to administer, but less flexible. It is created based on the usual carbs taken per meal and the usual exercises per day. DF does not take into consideration any changed amounts of carbs and exercise during the day. As a result, the patient must stick with a specific predefined diet and exercise pattern to avoid hypoglycemia. There are two main types of DF regimen. The once-daily regimen is when the patient takes one shot in the morning or in the evening via long-acting insulin (e.g. *detemir*). The twice-daily regimen is when the patient takes two shots (i.e. 2/3 of TDD as the morning dose and 1/3 of TDD as the evening dose). The morning dose is divided again into 2/3 in an intermediate-acting dose (e.g. *NPH insulin*), and 1/3 in a short-acting dose (e.g. *regular insulin*). The evening dose is also divided, but in half, with 1/2 in an intermediate-acting dose, and 1/2 in a short-acting dose. The patient mixes the two types of insulin in one syringe for morning and evening doses. There are premixed insulins such as the 70/30 preparation, which is suitable for morning shots, and a 50/50 preparation, which is suitable for evening shots. They are based on premixed insulins (e.g. *Novolin 70/30*, *Humalog Mix 50/50*) administered in one or two shots per day. For this version of the ontology, we assume the patient will not take any snacks between meals just to simplify the calculations.

To create a customized plan, the selected insulin regimen must first be approved by the patient, and second be checked for compatibility with patient conditions. To personalize the treatment plans, we must check the drug-drug, drug-disease, and drug-food interactions. A patient currently taken drugs, including insulin, can conflict with other drugs, diseases, or foods. For example, *Novolog (insulin aspart)* is contradicting with more than 125 drugs (e.g. *gatifloxacin, macimorelin*) and some diseases (e.g. *hypokalemia*). In addition, many drugs affect the blood glucose level or the body’s sensitivity to insulin. Drugs such as *corticosteroid*, *octreotide*, *beta-blockers*, *epinephrine*, *thiazide diuretics*, *statins*, *niacin*, *pentamidine*, *protease inhibitors*, *L-asparaginase*, *antipsychotics*, *cortisone*, *Seroquel*, *niacin*, *beta 2 agonists*, and *diuretics* cause hyperglycemia, but drugs such as *quinine* cause hypoglycemia. Diseases such as *metabolic syndrome* and *acromegaly* cause hyperglycemia, but pregnancy and disorders that affect the liver, heart, or kidney can cause hypoglycemia. All of these factors must be taken into consideration to create a real customized plan. To support interoperability, accuracy, and medical acceptance, we employed many sources to build the TP knowledge including standard CPGs, medical experts, and official websites. We use SWRL to represent monitoring and treatment knowledge in the form of rules.

**Fig. 7:**
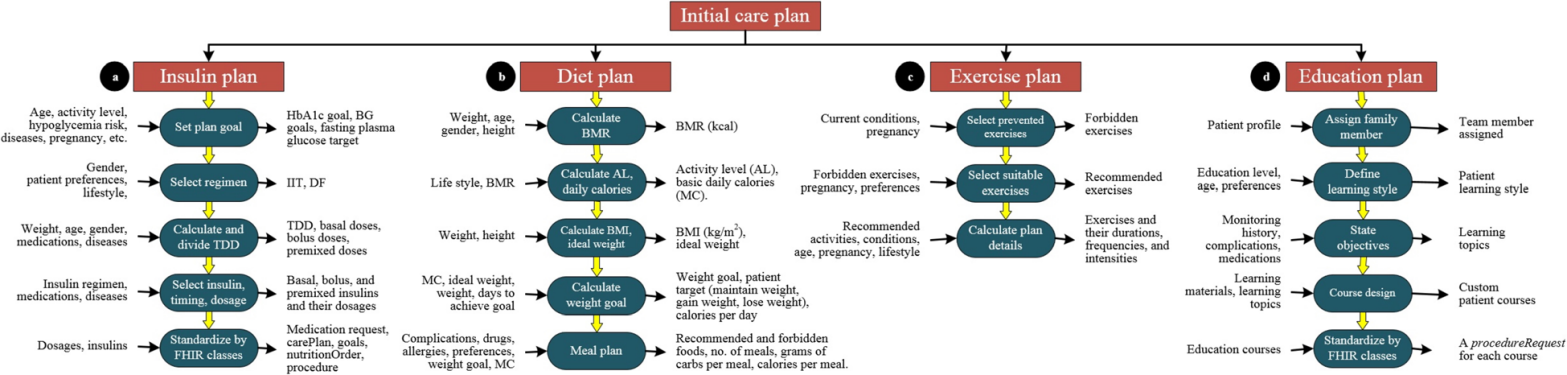
Initial care plan components. These are the detailed modules of the initial care plan including insulin, diet, exercise, and education modules


As shown in Fig. 7 (a), the creation of an insulin plan has a set of steps starting with setting goals for HbA1c, weight, and BG levels and ending in a standardization process. We faced many challenges to prepare the medical knowledge implemented in this ontology. As a result, the TPs implemented in this version of the ontology do not show the full representation and reasoning capabilities of our proposal. Based on the patient’s age, we determine whether the patient is a *child*, *adolescent*, *adult*, or *oldAdult*. FASTO has 140 SWRL rules. The list of SWRL rules is disclosed in the Additional file 1. We will give some examples to illustrate the idea. For example, Rule 1 identifies adult patients.

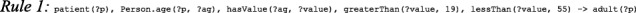


Results of these rules with the patient’s pregnancy status are used to define plan goals, as shown in the Rule 2 example. In addition, if the patient has some *cardiovascular diseases* or experienced *hypoglycemia*, they will also affect the goals definition.
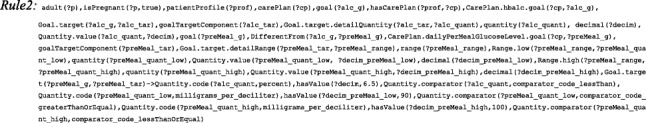


Rule 2 determines the TP goals as “*IF patient is adult AND pregnant THEN goals are HbA1c<6.5% AND pre-meal BG >=90mg/dL and <=100mg/dL*”. These values can easily be changed based on new evidence. This knowledge is standardized according to FHIR, and its UoMs are based on UCUM.

Selection of a regimen is based on patient preferences if the patient is an adult, but for pregnant and child, IIT is medically the best choice. For example, Rule 3 decides the regimen for an adult pregnant patient.
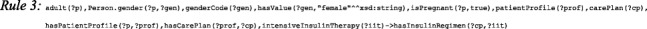


According to the selected regimen, we calculate the TDD and divide it into shots for the entire day. Patient weight is an SSN ontology observation, and its value is represented as the FHIR ObservationValue instance. As a result, the ontology will accept sensor observations and standardize them according to FHIR. In this version of our ontology, we depend mainly on patient weight (W) to determine TDD, as shown in Eq. 1. For DF regimen, the resulting TDD is divided into the morning dose (MD) and evening dose (ED), as shown in Eq. 2. The morning dose is divided into long or intermediate-acting insulin (MD_L_) and short-acting insulin (MD_s_), as shown in Eq. 3. The evening dose is divided into long or intermediate-acting insulin (ED_L_) and short-acting insulin (ED_S_), as shown in Eq. 4.

1 $$ \mathrm{TDD}=0.6\ast \mathrm{W}\ \mathrm{unit}/\mathrm{day} $$

2 $$ \mathrm{MD}=\frac{2}{3}\ast \mathrm{TDD},\mathrm{ED}=\frac{1}{3}\ast \mathrm{TDD} $$

3 $$ \mathrm{M}{\mathrm{D}}_{\mathrm{L}}=\frac{2}{3}\ast \mathrm{M}\mathrm{D}=\frac{2.4}{9}\ast W\  units,\mathrm{M}{\mathrm{D}}_{\mathrm{S}}=\frac{1}{3}\ast \mathrm{M}\mathrm{D}=\frac{1.2}{9}\ast \mathrm{W}\ \mathrm{units} $$

4 $$ \mathrm{E}{\mathrm{D}}_{\mathrm{L}}=\frac{1}{2}\ast \mathrm{E}\mathrm{D}=0.1\ast W\  units,\mathrm{E}{\mathrm{D}}_{\mathrm{S}}=\frac{1}{2}\ast \mathrm{E}\mathrm{D}=0.1\ast \mathrm{W}\ \mathrm{units} $$

Rule 4 calculates the TDD of any insulin regimen based on patient weight.
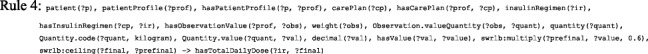


Rule 5 divides TDD of a fixed regimen into four parts according to the previous equations.
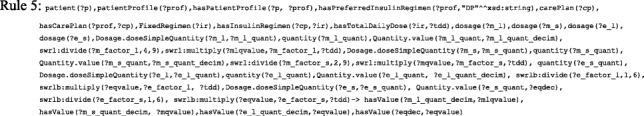


After calculating dosages, we select the most appropriate insulin depending on the patient’s current complications and currently taken drugs. This check prevents contradictions with the patient’s current state. For example, Rule 6 checks if the patient is currently taking a drug that is contradicted with insulin *detemir* (e.g. *testosterone*, *beta-blockers*, *decongestants*, and *hydrochlorothiazide*)


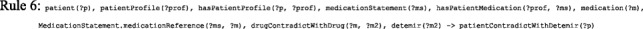


These contradictions are used to select the suitable insulins. For example, Rule 7 select *glargine* long acting insulin if patient contradicts with *detemir* insulin.
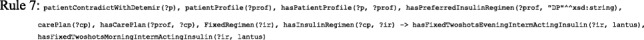


Each DF regimen is assigned two insulins (i.e. long acting and rapid acting), and each one has two dosages. For example, if W = 30 kg, then MD_L_ = 8 *units*,MD_s_ = 4 *units*, ED_L_ = 3 *units*, and ED_s_ = 3 *units*. The patient mixes the morning dose in one syringe and the evening dose in another. Premixed insulins are not considered in this version of the ontology. This plan will be maintained every 3 months based on HbA1c and weight goals. The initial IIT insulin regimen divides TDD into basal dose (BA) and bolus dose BO, as shown in Eq. 5, Eq. 6, and Eq. 7:

5 $$ \mathrm{BA}=\mathrm{f}\ast \mathrm{TDD} $$

6 $$ \mathrm{BO}=\left(1-\mathrm{f}\right)\ast \mathrm{TDD} $$

7 $$ Premeal\ dose=\frac{BO}{3} $$

where f is a factor with values 0.3, 0.4, 0.5, or 0.6, based on patient conditions. Current medical literature does not clearly define when to use each factor, but most CPGs use 0.5. The BA dose is given as one shot taken in the morning or evening, or divided into two shots. Rule 8 determines the basal dose using UCUM, its route of administration according to SCT, and its timing according to SCT.
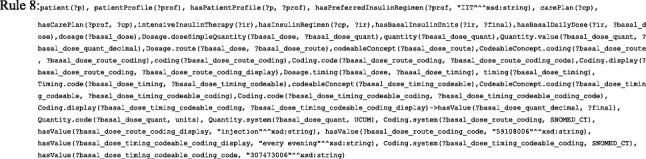


In the same way, the BO dose is divided into three shots to be taken before the three meals. These shots will change according to the patient’s real-time BG, meal carbs, and exercise. As modeled in the DP regimen, names of basal and bolus insulins are determined according to patient’s current medications and complications.

The last step is the standardization of the resulting knowledge. This straightforward step is achieved by mapping the modeled knowledge to a medicationRequest object. This class is connected to the carePlan’s carePlanActivityComponent by *reference* object property, as show in Fig. 5.

***Diet plan definition*** Diet is critically related to T1D TPs because patients on insulin therapy always gain weight [60]. Obesity is associated with insulin resistance and cardiovascular diseases, where patients are considered to have “double diabetes.” As a result, diet and insulin sub-plans are tightly linked. Sometimes, we adjust insulin regimens to facilitate weight management; other times, we adjust insulin dosages to cover changing meal carbs. Diet is the meal planning process. The diet plan defines what to eat, in what quantities, and when [54], in order to maintain a near normal BG level, blood pressure, lipid level, and body weight. This is known as medical nutrition therapy. Total meal carbs has the greatest effect on glycemic control (more than proteins and fats), and this fact has been confirmed in all CPGs. As a result, we depend on the amount of carbs to create the diet plan. Mainly, the plan has three meals, and each meal has a specific amount of carbs. The plan does not determine specific foods for each meal, but it provides basic food groups according to the patient’s preferences, activity level, food-drug and food-disease contradictions, age, and weight. In addition, the diet plan determines the weight goal of carePlan. The diet plan is defined in five steps, as shown in Fig. 7 (b).

*First*, we calculate the patient basal metabolic rate (BMR) based on the Harris-Benedict Equation [61]. It depends on the patient’s weight (in pounds or kilograms), height (in inches or centimeters), and age (in years). BMR is calculated for men and women using the metric system (cm/kg) by using Eq. 8 and Eq. 9, respectively. Using imperial measurements (inches/pounds), it is calculated for men and women by using Eq. 10 and Eq. 11, respectively.

8 $$ \mathrm{BMR}=\left(13.75\ast \mathrm{weight}\ \mathrm{in}\ \mathrm{kg}\right)+\left(\mathrm{5,003}\ast \mathrm{height}\ \mathrm{in}\ \mathrm{cm}\right)-\left(6.755\ast \mathrm{age}\ \mathrm{in}\ \mathrm{years}\right)+66.5 $$

9 $$ \mathrm{BMR}=\left(9.563\ast \mathrm{weight}\ \mathrm{in}\ \mathrm{kg}\right)+\left(1.85\ast \mathrm{height}\ \mathrm{in}\ \mathrm{cm}\right)-\left(4.676\ast \mathrm{age}\ \mathrm{in}\ \mathrm{year}\right)+655.1 $$

10 $$ \mathrm{BMR}=\left(6.2\ast \mathrm{weight}\ \mathrm{in}\ \mathrm{pounds}\right)+\left(12.7\ast \mathrm{height}\ \mathrm{in}\ \mathrm{in}\mathrm{ches}\right)-\left(6.76\ast \mathrm{age}\ \mathrm{in}\ \mathrm{years}\right)+66 $$

11 $$ \mathrm{BMR}=\left(4.35\ast \mathrm{weight}\ \mathrm{in}\ \mathrm{pounds}\right)+\left(4.7\ast \mathrm{height}\ \mathrm{in}\ \mathrm{in}\mathrm{ches}\right)-\left(4.7\ast \mathrm{age}\ \mathrm{in}\ \mathrm{years}\right)+655.1 $$

A diet sub-plan is implemented with the FHIR nutritionOrder class, and BMR is added to the plan as supplementary knowledge using the nutritionOrderSupplementComponent class (see Fig. 5). We created SWRL rules to calculate BMR for male and female patients according to the used UoM. The FHIR patient’s age and SSN weight and height observations are applied based on Eqs. 8, 9, 10 and 11. Rule 9 gives an example for calculating the BMR of a female by using the metric system (cm/kg). The resulting BMR has a UoM from the UCUM, and the value has the SCT code “*165,109,007*.”
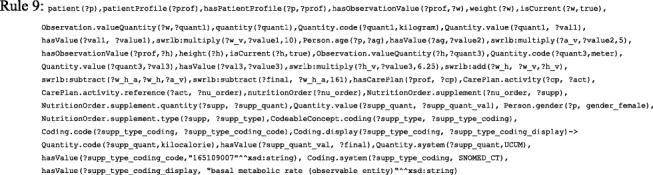


*Second*, we determine the patient’s activity level (AL) based on lifestyle (Table 2); then, we multiply AL with the BMR to get the number of calories to maintain the current patient weight (MC), as shown in Eq. 12.

12 $$ \mathrm{MC}=\mathrm{AL}\ast \mathrm{BMR} $$

**Table 2:** Activity levels of patients

Life style	Activity level value
Sedentary (little or no exercise)	1.2
Lightly active (light exercise/sports 1–3 days/week)	1.375
Moderately active (moderate exercise/sports 3–5 days/week)	1.55
Very active (hard exercise/sports 6–7 days a week)	1.725
Extra active (very hard exercise/sports & physical job or 2x training)	1.9

For example, Rule 10 determines the AL of the patient, and Rule 11 calculates his/her MC.
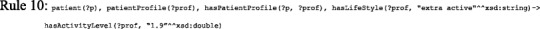

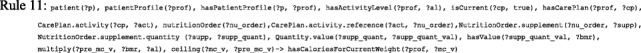


*Third*, we calculate the patient ideal weight (IW) because the current weight may not be the healthier weight. We follow the WHO, where the healthy BMI range is: 18.5 to 25 for both men and women. BMI can be calculated according to Eq. 13. As a result, the IW is in the range calculated by Eq. 14 and Eq.15.

13 $$ \mathrm{BMI}\left(\mathrm{kg}/{\mathrm{m}}^2\right)=\mathrm{Weight}\ \mathrm{in}\ \mathrm{kg}/{\left(\mathrm{Height}\ \mathrm{in}\ \mathrm{m}\right)}^2 $$

14 $$ \mathrm{Lowest}\ \mathrm{IW}\ \mathrm{in}\ \mathrm{kg}\ \left(\mathrm{LIW}\right)=18.5\ast {\left(\mathrm{Height}\ \mathrm{in}\ \mathrm{m}\right)}^2 $$

15 $$ \mathrm{Highest}\ \mathrm{IW}\ \mathrm{in}\ \mathrm{kg}\ \left(\mathrm{HIW}\right)=25\ast {\left(\mathrm{Height}\ \mathrm{in}\ \mathrm{m}\right)}^2 $$

SWRL rules are tailored to calculate previous equations. For example, Rule 12 calculates LIW and HIW weight values.
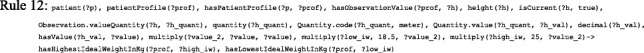


*Fourth*, by comparing the IW range with the current weight (W), we determine how many calories the patient needs in order to maintain, gain, or lose. In addition, we determine the carePlan’s weight goal (WG). If W ∈ [LIW, HIW], patient has normal weight. In this case, the patient has to maintain his/her weight, and the WG should equal W. If W < LIW, the patient is underweight; he/she needs to gain weight of at least LIW − W kg. To gain this weight, the patient needs extra (LIW − W) ∗ 7700 calories. The WG should be at least W + (LIW − W) kg. If W > HIW, the patient is overweight or obese; he/she needs to lose weight of *W* − HIW kg. To lose this weight, patient needs to reduce calories by (W − HIW) ∗ 7700, or he/she will have to depend on the exercise plan. In this case, WG is at most W − (HIW − W) kg. We must define the period in days (d) required to lose or gain weight of (OW) and then determine the number of calories to reduce or add per day with (OW ∗ 7700)/d. The number of calories per day (CpD) is calculated as follows. CpD = MC, and if W ∈ [LIW, HIW]; CpD = MC + (OW ∗ 7700)/d, if W < LIW; and CpD = MC − (OW ∗ 7700)/d, if W > HIW. The grams of carbs are equal to CpD/4, which are distributed in meals at 30% for breakfast, 35% for lunch, and 35% for dinner. For example, Rule 13 determines the weight goal and the total daily calories for a patient who is of overweight. In addition, it codes the results using SCT and UCUM.
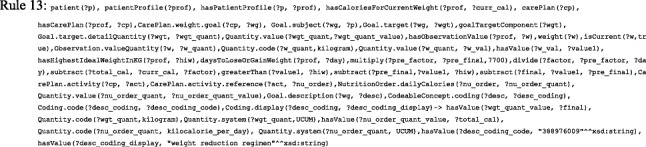


*Fifth*, we determine the number of meals (i.e. three meals for this version of FASTO), the number of calories for each meal, and the types of food that are allowed and forbidden. This step depends mainly on the patient’s preferences, on WG, and on current medications and complications. According to the selected regimen, the patient may or may not be able to change the meal carbs, as will be shown next.

The nutritionOrder class supports general knowledge about diet objects, including the diet’s *patient*, *dateTime*, *encounter*, *allergies*, *preferred and excluded foods*, *nutrients*, etc. (see Fig. 5). However, we have to specifically determine each meal’s carbs and calories to be able to perform real-time monitoring. As a result, we extend the nutritionOrder resource to incorporate meal knowledge.
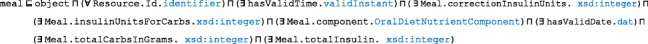


We add to nutritionOrder the object properties of *NutritionOrder.meal* and *NutritionOrder.dailyCalories* to define the order’s meal and the total daily calories, respectively. Every order is linked to a single meal. As a result, we connect a meal class to the nutritionOrder class, which is critical for insulin, diet, and exercise management. In this version of FASTO, we concentrate on whole calories and carbs per meals. The ontology defines the recommended foods, but it does not define specific foods and specific quantities. Please note, the required knowledge to implement specific foods is defined in FASTO, but the required SWRL was not added. For example, Rule 14 determines forbidden foods based on the patient’s current medications.
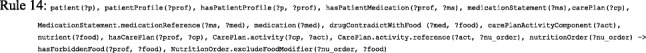


***Exercise plan definition*** The exercise plan is a vital component of T1D management. Exercises improve insulin sensation. As a result, the combination of regular exercise with diet is critical for a successful care plan in order to prevent cardiovascular diseases and maintain normal BG levels. This plan defines the type of activities suitable for the patient, their timing and intensity, and their periods to avoid hypoglycemia. Patients on the DF regimen are not allowed to change their ALs as defined according to their lifestyles. On the other hand, patients on IIT can change exercises, and in real-time monitoring, FASTO adjusts the carb grams and insulin dosages accordingly. In this step, we define only the basic exercise sub-plan for both IIT and DF regimens, as shown in Fig. 7 (c). We extend FHIR resources by adding the exercisePlan class based on the Schema.org (*https://schema.org*) standard. To define the patient’s exercise sub-plan, we connect the exercisePlan class to the carePlanActivity class by using its *reference* object property.
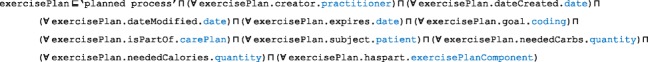


This design supports the assignment of many exercise components to the same exercise plan, and each component has its own *frequency*, *intensity*, *repetition*, *exercises*, and *duration*.
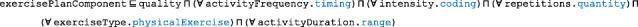


In addition, each exercise has its own properties, including *contradictions*, *code*, *metabolic equivalent of task (MET) value*, *total weekly duration*, etc.


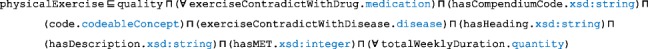


Please note, unplanned exercises and their associated changing procedures in insulin dosages and carb grams are handled in the real-time monitoring phase. *First*, we determine the patients who are forbidden from doing exercises according to their current conditions. For example, a pregnant woman is forbidden from exercises if she is [1] extremely underweight (BMI < 12 kg/m^2^), [2] has *hypertension (*e.g. *preeclampsia)*, *morbid obesity*, *placenta Previa*, *fetal anemia*, or *chronic bronchitis*. Rule 15 identifies patients with *hypertension*, *dyslipidemia*, *preproliferative retinopathy*, *nephropathy*, *cigarette smoking*, and *age > 30* as forbidden from doing exercises.
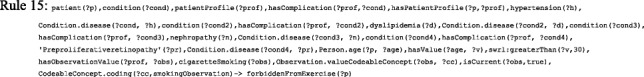


*Second*, according to the previous step, we determine the forbidden and recommended exercises according to the patient conditions (e.g. complications and pregnancy) and preferences. For example, Rule 16 collects the exercises not allowed for a patient according to his/her diseases. Rule 17 determines the list of recommended exercises for patients if they are preferred and not forbidden. For example, if a patient has *foot ulcers*, then he/she must avoid *jogging*; a patient with *cataracts* should avoid *cycling*; a patient has *severe nonproliferative retinopathy* should avoid *jumping*, *jarring*, and *breath-holding* exercises. *Finally*, we define the regular exercise plans based on the selected exercises and patient’s age and conditions.
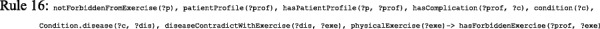

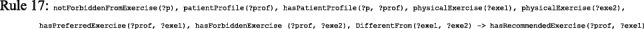


For interoperability, each final planned exercise in the exercisePlan class can be mapped to the procedureRequest instance in a straightforward way.

***Education plans definition*** Education is a crucial ongoing process to improve the patient’s decision-making ability, self-monitoring behaviors, problem solving ability, and active collaboration with the MH system. The main steps are shown in Fig. 7 (d). The patient could be a child or an elder adult, so a specific family member (i.e. a relative if any) must be assigned as the coordinator for the delivery of training courses. *First*, according to the patient’s age, language, and education level, a suitable learning style (i.e. reading, visual, auditory, games, or case studies) is selected. For example, Rule 18 states that a visual learning style (e.g. video, images, etc.), and reading are suitable for highly educated adults.
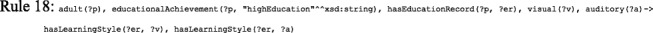


*Second*, the learning topics for the patient depend on his/her current conditions, including currently taken medications, complications, and insulin-monitoring history. The most common learning topics include *insulin*, *medications*, *diet*, *monitoring*, *emergency*, *exercise*, and *complications*. For example, Rule 19 asserts that if the patient is on IIT then he/she must take courses in glucose monitoring, insulin management, and diet management.
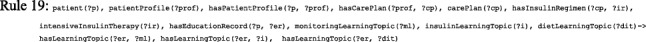


*Third*, each learning topic has many associated courses. For example, the insulin topic needs many courses, including type 1 diabetes mellitus, what insulin is and its types, insulin regimens, ways to inject, dosing, storage, adverse effects, allergies, and contradictions. The medication topic needs courses in the administration, dosages, adverse effects, and contradictions. The diet topic requires courses in weight loss, gain, and maintain; nutrition and their carbs; and carb counting. The monitoring topic has courses in BG pattern management, blood pressure monitoring, weight monitoring, lipid monitoring, and for calculating ISF and ICR. The emergency topic has courses in hypo/hyperglycemia symptoms and ways for their management. The complications topic has many courses, depending on the current complications of the patient. For each complication, a course is required to describe what it is, ways to manage its medications, and its contradictions. Finally, the exercise topic has courses for selecting sports and calculating the needed calories.

FASTO defines a set of courses for each topic and selects a customized format for the patient’s courses according to his/her defined learning style and learning topics. For example, Rule 20 assigns a set of courses for patients having insulin topic and who prefer reading style. In this version of FASTO, we manually tailored a set of courses for the proposed styles and topics. In the future, we will link machine-learning techniques to select appropriate courses and customize these courses automatically.
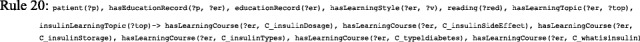


*Finally*, each course is mapped to a procedureRequest instance and sent to the patient’s mobile device (Rule 21).
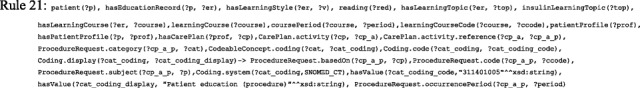


##### Real time plan adjustment

Now, we concentrate on patients following the IIT regimen in order to adjust their insulin dosages in real time. Many situations necessitate adjustment in basal and bolus insulin dosages, including carb intake per meal, pre-meal glucose level, anticipated physical activities, weight changes, newly taken drugs, fasting blood glucose, and new complications (including surgeries and infections). Patients on IIT measure BG at least four times daily (e.g. before meals, at bedtime, prior to exercise, when suspecting low blood glucose, after hypoglycemia, and prior to driving). These sensor values are used to adjust the bolus insulin dosages. This adjustment is based on the two evaluation factors of ISF (i.e. correction factors) measured in millimoles per liter per unit (mmol/L/U) or milligrams per deciliter per unit (mg/dl/U) and ICR measured in carbs/U [55]. The pre-meal and bedtime goals are used to manage BG in real time. On the other hand, temporal abstraction of collected sensor data is used to study the behavior of these observations and determine patterns of glucose management (e.g., weight increases, high glucose after every lunch, hypoglycemia every night, etc.). These patterns are used to adjust basal insulin dosages as follows.

*First*, we calculate the patient’s ISF. The ISF is the number of BG points that are reduced by one unit of bolus insulin. It depends on the UoM for BG. If BG is measured in *mg/dl* then we use the *1800 rule* (i.e. f = 1800 in Eq. 16), and if BG is measured in *mmol/L* then we use the *100 rule* (i.e. f = 100 in Eq. 16).

16 $$ \mathrm{ISF}=\frac{\mathrm{f}}{\mathrm{TDD}} $$

The ISF is used to adjust the bolus regimen based on the planned range for the target per-meal BG. For example, if the patient has a pre-meal BG goal of [100–150] mg/dl and ISF = 50, then based on his/her current BG level, the corrections to bolus doses can be described as shown in Table 3.

**Table 3:** The role of ISF in the adjustment of meals’ bolus insulin dosage

Pre-meal blood glucose	Before breakfast	Before lunch	Before dinner
< 70	−1	-1	-1
100–150	Planned dose / carbs counting	Planned dose / carbs counting	Planned dose / carbs counting
150–200	+ 1	+ 1	+ 1
200–250	+ 2	+ 2	+ 2
250–300	+ 3	+ 3	+ 3
> 300	+ 4	+ 4	+ 4

Table 3 assumes that a patient consumes the same amount of carbs for every meal, but this is not realistic. As a result, ICR is used to manage the dynamic number of grams for every meal. Using this approach, we replace the planned dose in Table 3 by the dosage calculated in real time using ICR and the meal’s carb grams. *Second*, according to the TDD, a patient’s the ICR is calculated using the *500 rule*, as shown in Eq. 17.

17 $$ \mathrm{ICR}=\frac{500}{\mathrm{TDD}} $$

ICR is the number of carb grams that will be covered by one unit of bolus insulin. As a special case for children, if TDD < 10 U, then we use the *300 rule* (i.e. ICR = 300/TDD). For example, Rule 22 determines the ICR for a child with TDD < 10 U.
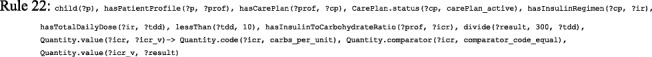


*Third*, we put them all together to provide patients with real-time advice. Real-time advice can be “*increase or decrease this meal bolus insulin dose by n units*,” “*eat m extra grams of carbs*,” etc. FASTO tries to balance the basal insulin dosage with meal carbs and exercises. FASTO has all the needed semantics to implement this knowledge. We build a set of robust SWRL rules to make the most suitable decisions. Real-time monitoring has two branches of mealtime bolus insulin correction and pattern management of basal and bolus insulin dose adjustments.

For a specific meal, if FASTO only received the current BG sensor observation of (CBG) in mg/dl, and another observation for the needed-to-eat meal carbs (MC) in grams, then FASTO uses these two values and calculates the meal’s bolus insulin dosage as follows. Please note that the patient is not planning to do any exercises. For simplicity, we discuss the required calculations with an example. If patient X has TDD = 50 U, and is planning to take a meal with MC = 60 g of carbs, CBG = 210 mg/dl, and pre-meal BG (PBG) goal is 120 mg/dl. According to Eq. 16 and Eq. 17, ICR = 500/50 = 10 carbs/U, and ISF = 1800/50 = 36 mg/dl/U.

1. Calculate the difference in BG as DBG = CBG – PBG. If the current BG is the same as the target BG, there will be no effect from this BG observation. Regarding patient X, DBG = 210 − 120 = 90 mg/dl.
2. Based on ISF, calculate the insulin units needed to correct BG level by using N1 = DBG/ISF. Regarding patient X, N1 = 90/36 = 2.5 U.
3. Based on ICR, calculate the insulin units needed to cover MC carbs by using N2 = MC/ICR. Regarding patient X, N2 = 60/10 = 6 U.
4. Calculate the meal bolus insulin dose using BD = N1 + N2. Regarding patient X, BD = 2.5 + 6.0 = 8.5 U.
5. If BD is positive, then the patient needs to take BD units of insulin. If BD is zero, then this bolus dosage must be skipped. If BD is negative, then the patient must take some more carbs to increase the BG level. Regarding patient X, the bolus dosage needed to balance BG for this meal is 8.5 U.

All of the above calculations are done by Rule 23.
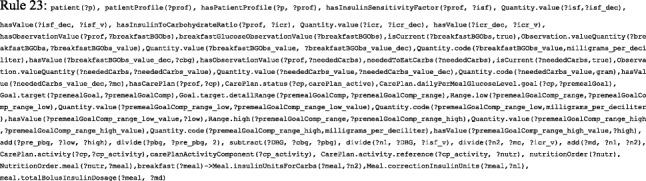


FASTO could receive three observations for CBG, meal carbs for MC, and exercise. In this case, the patient is expected to exercise within 3 hours after taking the meal. If CBG > 250 mg/dl, CBG < 80 mg/dl, or the patient has *diabetic ketoacidosis*, then the patient is forbidden from exercising. Exercise is represented by its standard compcode [62] and its duration in hours (h). The compcode associates an activity with its intensity and MET. We combine MET, patient weight (kg), and duration (h) to get the burned calories (BC) for this activity (see Eq. 18).

18 $$ \mathrm{BC}=\mathrm{MET}\ast \mathrm{weight}\ \left(\mathrm{kg}\right)\ast \mathrm{duration}\ \left(\mathrm{h}\right) $$

The BC value is used to calculate the carb grams to be increased (BC/4). As the patient is already at the mealtime, the BC value is used to determine the need-to-reduce insulin units (BC/4)/ICR. As a result, the bolus BD is calculated as shown in Eq. 19. For space restrictions, we will not give examples of SWRL rules.

19 $$ \mathrm{BD}=\mathrm{N}1+\mathrm{N}2-\left(\left(\mathrm{BC}/4\right)/\mathrm{ICR}\right) $$

If exercising is not within 3 hours of a meal, FASTO will receive the CBG and exercise only. The decision depends on the patient’s current condition, i.e. underweight, overweight, or obese. If the patient is underweight or at normal weight, then equivalent carb grams (BC/4) is needed, and its insulin units, (BC/4)/ICR, is added to N1. If he/she is overweight or obese, then the equivalent insulin units are only subtracted from N1.

Basal insulin changes according to the pattern management process. It is more suitable to apply some machine-learning algorithms to the cloud-based EHR data to discover these patterns. However, we can do it using FASTO semantics. To discover patterns in the BG or weight measurements, we must use data from at least three consecutive days, and the compared values must be for the same time of day. The dosage changes must be between 10% to 20% of the planned dosage. Table 4 illustrates 3 days of measurements for the BG of a patient with a goal BG of 70–140 mg/dl. These measurements show that all values are within the goal range except the before-breakfast values. As a result, bedtime basal insulin dose must be increased by at least 10%. Please note that the before dinner BG on May 6 was outside of the goal’s range, but we cannot make any decision based on this single value.

**Table 4:** Three consecutive days of BG measurements

BG	Before breakfast	Before lunch	Before dinner	Before bedtime
Date				
May 5	320	104	96	86
May 6	296	123	300	136
May 7	341	197	92	111

The algorithm used to manage the discovered patterns is as follows. High/low before-lunch BG means increase/decrease the before-breakfast bolus dose. High/low before-dinner BG means increase/decrease the before-lunch bolus dose. High/low before-bedtime BG means increase/decrease the before-dinner bolus dose. High/low before-breakfast BG means increase/decrease the bedtime-basal dose.

### Cloud-based EHR database

**Fig. 8:**
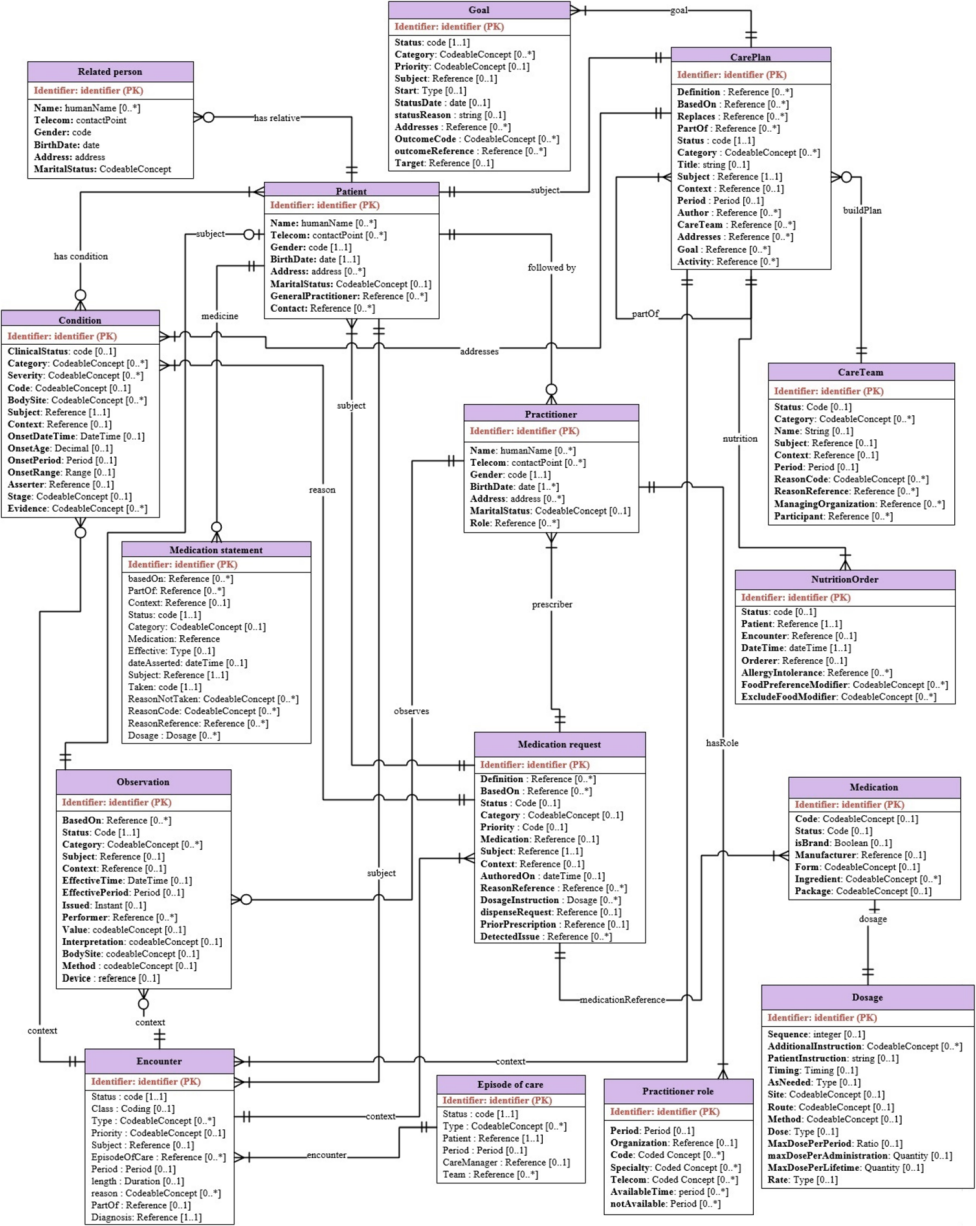
Cloud EHR database based on FHIR resources. This is a part of the created database from FHIR standard resource. This database is stored on the cloud to collect all of the patient profile data in a standard way

The patient historical data from distributed EHRs and real-time observations from a WBAN are collected, integrated into, managed, and queried from a cloud-based EHR database in standardized form based on HL7 FHIR. It is convenient to use a NoSQL database like the MongoDB document database because a JSON document is equal to a database document, and less mapping is required. However, RDBs are more popular and more stable, and most of the current EHR databases are in RDB format. In addition, HL7 provides a standard RDB implementation (i.e. FHIRBase: *http://fhirbase.github.io**)* for FHIR resources. Therefore, it is inevitable to use a relational database to store data objects that are required, in order for all system modules to interoperate. We implemented and customized an RDB based on FHIR schema. Different from FHIRBase, this database is designed based on mapping FHIR resource to RDB table and resource elements to attributes, relations, or other tables. An FHIR resource can be mapped to multiple tables to generate a normalized RDB. The database was designed according to the previously selected resources and their designed profiles, as shown in Table 1. Many FHIR elements (e.g. imaging elements) have been ignored from the selected resources to concentrate on our main target. Figure 8 shows a fragment of the designed relational data model. Attribute data types are modeled in a high-level way to preserve the simplicity of the diagram. To populate this RDB, a cloud-based FHIR server sends RESTful requests to backend systems and to mobile devices to collect patient data, both of which reply by the required FHIR JSON resources. These JSON documents are mapped to their equivalent RDB elements in a straightforward way. Next, the database records are used to create FASTO’s ABOX individuals and assertions. In addition, this database stores the patient management history from the FASTO ontology, including patients’ previous TPs. The conversion among sensor raw data, EHR database records, and ontology instances is managed by the standard HL7 FHIR data model and standard terminologies (e.g. SCT, LOINC, etc.).

## Backend EHR systems module

**Fig. 9:**
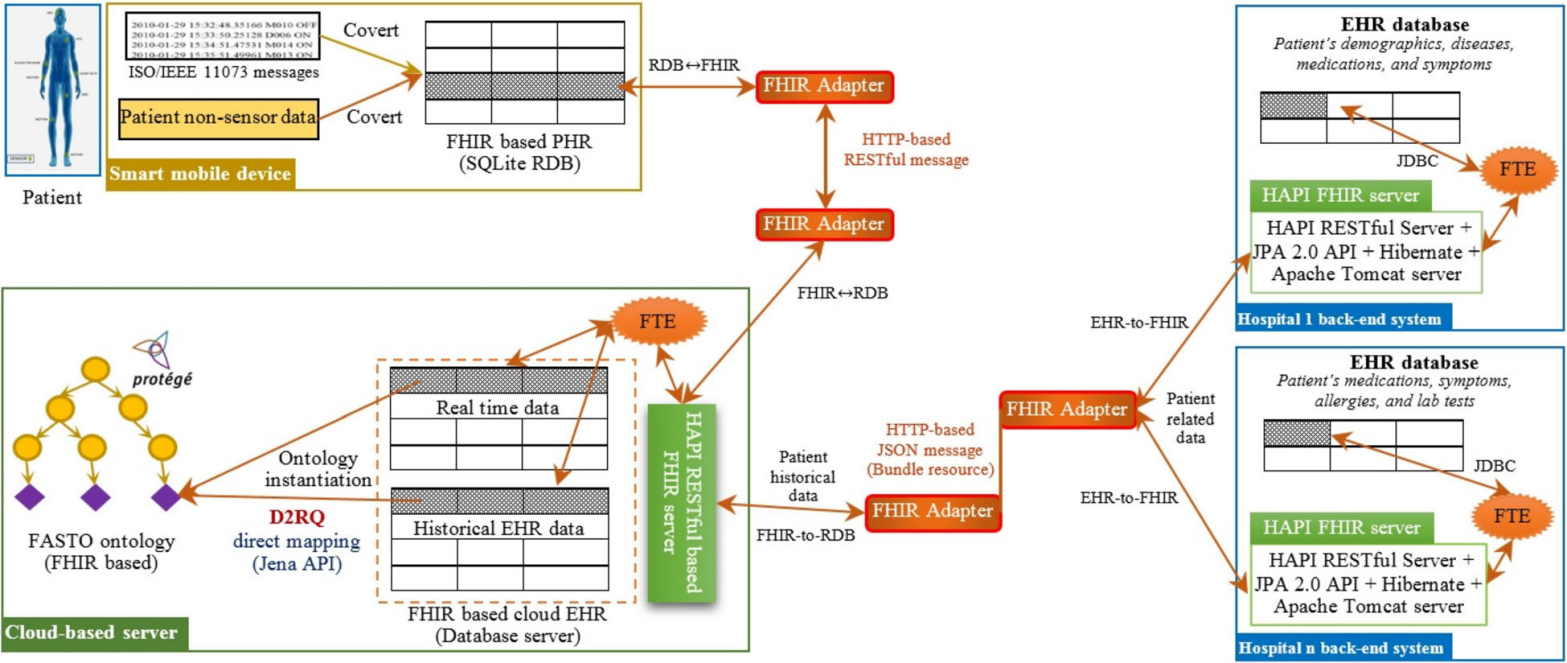
Data transformation process in the proposed framework. These are the whole connections in the system among patient, backend hospitals, and the cloud based CDSS. The connections are based on FHIR standard adapters

To support interoperability and the seamless integration of collected data from sensors and EHR backend databases, we provide a common interface between sensors, aggregators (mobile phones), CDSSs, cloud-based storage environments, and backend hospital EHR systems. This interface is based on FHIR adapters, which transform among FHIR resources and internal data structures of all system modules. As shown in Fig. 9, RESTful FHIR servers need to be implemented in the cloud module and EHR systems. These servers are based on the DSTU3. The servers are responsible for mapping between local databases and RESTful queries. In addition, they transform sensor and EHR data to FHIR resources, which can be transmitted as HL7 JSON messages between system components. The collected messages are mapped to cloud-based EHR database records, which are used to instantiate the FASTO ontology. All transformation processes are implemented via FHIR transform engine (*http://www.openmapsw.com/products/FTE.htm*). This engine does not make a hard structure-to-structure mapping, but maps both database structure and FHIR resources into one common logical model, i.e. the FHIR resource class model. The FHIR transform engine and FHIR servers are integrated based on the standard HL7 application-programming interface (HAPI: *http://hapifhir.io*) v 3.4.0. All Java implementations that support the proposed FHIR-based framework can be found in the FHIR official site (*http://hapifhir.io/index.html*).

To collect a patient’s historical profile, the cloud-based FHIR server sends HTTP-based RESTful search requests to the distributed hospital EHR systems to collect the patient history based on the patient’s medical ID. Each backend system has an implemented FHIR server, which translates the search string of the request into its internal search command (e.g. a SQL SELECT query), and runs this query. Query results are converted to FHIR’s JSON resources and are sent as HTTP response messages to the cloud. The cloud system translates the message into an INSERT SQL statement to manage the patient’s historical data. The structural and semantic mapping between FHIR resources and RDBs is handled by an object relational mapping (ORM) API. Oracle’s Java persistence API (JPA) standard with Hibernate is more suitable for implementing these mappings. This implementation is expected to handle interoperability challenges in an efficient and sufficient way. The data models of PHRs and backend EHRs are transparent to the CDSS, thanks to the FHIR servers implemented in these modules. This design supports extensibility of an EHR ecosystem without affecting the currently running modules. In our proposed system, we map FHIR resource instance elements to RDB tables and attributes, and RDB tables and attributes to FASTO instances and properties.

## Results

FASTO reuses the conceptual model provided by the BFO foundational ontology. As a result, it inherits all the modeling properties and expressivity characteristics of the upper-level model. The expressivity of FASTO falls under the SHOIN (D) description logic. The FASTO ontology was designed with extensibility in mind. Each phase of the development process is evaluated separately to measure its accuracy and completeness. The ontology can be adapted to other domains, and it can be extended by adding new knowledge for T1D management. The ontology evaluation comprises two stages: evaluation of intrinsic properties (i.e. a technical evaluation) and evaluation of its actual use (i.e. an application evaluation). In this section, we evaluate the FASTO semantic model by these stages.

### Ontology verification and metrics

The ontology is implemented using Protégé 5.1 (*https://protege.stanford.edu/*) and rule-based reasoners (e.g. Pellet). The technical evaluation is verification and validation of the ontology, which assesses the consistency, correctness, and completeness of the ontology knowledge. A review of ontology metrics reveals a variety of metrics aiming to assess and qualify an ontology [63]. An ontology evaluation has many different qualitative and quantitative criteria, which help to uncover errors in implementation and inefficiencies in the modeling. However, no evaluation techniques, alone or in combination, can guarantee high-quality ontologies. Every evaluation methodology partially addresses specific issues.

An ontology-level evaluation by Pellet and HermiT reasoners reported valid ontology consistency and ontology taxonomy. Every rule in the list of SWRL rules was also validated, and the list as a whole is homogeneous and has no conflicts or redundancies. As a result, we assert that the proposed FASTO ontology functions in a proper way. To verify the FASTO ontology, we selected three evaluation methods: (a) an automated ontology evaluation tool named OntOlogy Pitfall Scanner (OOPS!) [64], (b) Protégé metrics, and (c) a manual evaluation. OOPS! is a web application that helps to detect some of the most common ontology development pitfalls such as cycles between classes in the hierarchy. The results of this evaluation suggested how the ontology could be manually modified to improve its quality. We evaluated FASTO by submitting it to OOPS!, which asserted that the ontology is free of any pitfalls. We used Protégé to collect the following fundamental metrics based on FASTO general structure:

- *generic ontology metrics* including the number of classes, properties, annotations, and instances;
- *concept or class axioms*, including subclass, equivalent, and disjoint axioms;
- *object property axioms*, including domain and range of properties. In addition, complex axioms regarding the equivalence, inverse, disjointness, functional, transitivity, symmetry, and reflexivity of properties have been collected;
- *data property axioms*, including domains and ranges properties. Further, similar to object properties, many complex axioms have been collected;
- *instance axioms*, including class assertions, same individual axioms, and different individual axioms; and
- *annotation axioms*, including domain and range annotations and annotation assertions.

Table 5 lists the FASTO ontology non-zero metrics, as provided by the “ontology metrics” view in Protégé. Our ontology is quite rich in classes, properties, axioms, and SWRL rules. This version of FASTO incorporates 9577 classes, 658 object properties, 164 data properties, and 460 individuals. In addition, 140 SWRL rules are added to implement the semantic logic of real-time monitoring and TPs. FASTO is publicly available, and can be freely downloaded from BioPortal (*https://bioportal.bioontology.org/ontologies/FASTO*).

**Table 5:** FASTO ontology metrics by Protégé

Metric	Count
Classes	9577
Axioms	60,045
Logical axioms	13,637
Equivalent classes	7
Functional object properties	9
Inverse object properties	21
Data properties	164
Data property assertions	228
Object property assertions	341
Class assertions	457
Annotation properties	127
Number of SWRL rules	140
Individuals	460
Annotation assertion	35,335
SubClassOf	10,024
Object properties	658
Object property domain	627
Object property range	628
SubObjectPropertyOf	649
Data property domains	130
Data property range	155
SubDataPropertyOf	159
DisjointClasses axiom	49
Description logic expressivity	SHOIN (D)

Manual evaluation by domain experts in medical practice and ontology engineering revealed a rational domain knowledge representation of FASTO. The major results are as follows. *Correctness*: our medical expert and ontology engineers asserted that the usage of classes, properties, axioms, and rules captures and accurately represents essential knowledge of real T1D treatment CDSS. This CPG based knowledge complies with the expertise of physicians. *Completeness*: FASTO is 100% complete regarding the coverage of medical knowledge. It is capable of representing all concepts, relationships, and rules constituting the patient profile, TPs, and real-time monitoring knowledge. In addition, it generates complete and medically acceptable TPs.

*Extensibility*: Based on the conceptual foundation of FHIR and the SSN, the FASTO generic ontology can be instantiated to represent complete diabetes cases. Furthermore, ontology modularization based on BFO offers monotonic extensibility to modify FASTO without violating the validity of the original ontology. *Conciseness*: The review process confirmed that FASTO does not include irrelevant or redundant knowledge. *Organizational fitness*: The ontology considered standards in every covered topic. It depends on the SSN to represent sensor data; it is based on FHIR to represent medical data and data types; it encodes medical data by standard terminologies; it utilizes standard CPGs to extract medical knowledge; and it is based on the BFO top-level ontology. Therefore, it supports the seamless integration of CDSS engines as transparent components in existing EHR ecosystems. It enables knowledge sharing and reuse without considerable reconfiguration of existing EHR systems.

### Comparison with existing ontologies

FASTO was developed to serve as a knowledge base for MH CDSSs. It is the most complete ontology for T1D management. To the best of our knowledge, there is no publicly available medical ontology for a mobile health CDSS that covers the medical domain and handles interoperability. The resulting medical knowledge is medically intuitive, and semantic interoperability is handled from all dimensions using standards (i.e. data models, terminologies, sensor data, upper-level ontologies, and communications). This ontology is more flexible and open, supporting extensions with new semantics. Table 6 provides a comparison between FASTO and six diabetes treatment ontologies based on 23 interrelated metrics. We checked if the ontology author has handled every metric or not. We used Yes/No to encode handled/not-handled metric, respectively.

**Table 6:** A comparison between DMTO and some existing diabetes treatment ontologies

Dimension	FASTO	DMTO [10]	DKOs [67]	Chen et al. [68]	Chalortham et al. [69]	Zhang et al. [70]	OntoDiabetic [71]
Purpose	Treatment	Treatment	Treatment	Treatment	Treatment	Treatment	Treatment
Type of diabetes	T1D	T2D	NA	T2D	T2D	T2D	NA
Available for reuse	Yes	Yes	No	No	No	No	No
Based on a unified top-level ontology	Yes	Yes	No	No	No	No	No
Encoded using standardized terminology	Yes	Yes	No	No	No	Yes	No
Based on OWL 2 and SWRL	Yes	Yes	Yes	Yes	Yes	Yes	Yes
Can be utilized in MH environments	Yes	No	No	No	No	No	No
Based on an interoperability standard	Yes	No	No	No	No	No	No
Decisions based on the whole patient profile	Yes	Yes	Yes	Only 6 tests entered by user	No	Yes	Yes
Supports interoperability with EHR distributed systems	Yes	No	No	No	No	No	No
Supports real-time patient monitoring based on WBANs	Yes	No	No	No	No	No	No
Supports data communication by standards as RESTful API	Yes	No	No	No	No	No	No
Handles interoperability with sensor data	Yes	No	No	No	No	No	No
Based on standard knowledge (i.e. collected from CPGs)	Yes	Yes	No	Yes	No	Yes	Yes
Uses a systematic method for creation	Yes	Yes	No	No	Yes	No	No
Delivers complete TPs with drugs, lifestyle, and education	Yes	Yes	No	No	No	Yes	No
Models diabetes drugs	Yes	Yes	No	Yes	No	No	Yes
Models drugs affecting glucose level	Yes	Yes	No	No	No	No	No
Models drug properties	Yes	Yes	No	Yes	No	No	No
Models T1D comorbidities	Yes	Yes	Yes	No	No	No	Yes
Reuses existing ontologies	Yes	Yes	No	No	Yes	No	Yes
Ontology coverage	Table 5	[10]	NA	18 drugs +6 rules	NA	NA	NA
Models temporal semantics	Yes	Yes	No	Yes	No	No	No

As shown in the table, all of the compared ontologies have limited coverage and handle the problem from only narrow viewpoints. FASTO is the most complete of the seven compared ontologies. All other ontologies have serious limitations regarding MH applicability, and interoperability with EHR distributed systems and sensor data. Regarding the ontology coverage metric, FASTO is the most complete ontology in the literature for T1D mobile monitoring. The proposed ontology covers all of these limitations and provides a mature solution that can be applied in an accurate way in existing medical environments.

### Coverage level evaluation

In this section, we present several SPARQL queries to demonstrate the usefulness and richness of FASTO. We evaluated its coverage by using a set of competency questions represented as SPARQL queries. These queries were evaluated by Protégé. FASTO is the richest ontology for T1D. It can represent any patient condition and is able to collect all types of data from either sensors or hospital databases. In addition, all knowledge related to interoperability between CDSS, WBANs, and EHR systems is modeled in a complete and standard way. Due to space restrictions, Table 7 shows a very short list of 17 competency questions and their corresponding SPARQL semantic queries. No publicly available ontologies discuss chronic-disease TPs, mobile and real time patient monitoring, and semantic interoperability the way FASTO does. In addition, because it is based on the modularization concept, FASTO is more rich, flexible, and open in order to handle new semantics.

**Table 7:** Ontology evaluation using competency questions

Competency question	SPARQL query	Competency question	SPARQL query
Q1. Find all patients who achieved their TP goals.	*SELECT DISTINCT*? *p WHERE {* *?p rdf:type fasto:patient;* *fasto:hasPatientProfile*? *prof.* *?prof fasto:hasCarePlan*? *plan.* *?plan fasto:isCurrent “true”^^xsd:Boolean;* *fasto:hasGoalAchieved “true”^^xsd:Boolean.* *}*	Q2. Who are the patients suffering from a specific disease with SNOMED CT code of X.	*SELECT DISTINCT*? *p WHERE {* *?p rdf:type fasto:patient;* *fasto:hasPatientProfile*? *prof.* *?prof fasto:hasComplication*? *cond.* *?cond fhir:Condition.code*? *code.* *?code fhir:Coding*? *coding.* *?coding fhir:Coding.code X;* *fhir:Coding.system “SNOMEDCT”.* *}*
Q3. What is the current pre-meal BG target for a patient with identifier X.	*SELECT*? *code,*? *system,*? *value WHERE {* *?p rdf:type fasto:patient;* *fhir:Resource.ID*? *id.* *?id fhir:Identifier.value X.* *?p fasto:hasPatientProfile*? *prof.* *?prof fasto:hasCarePlan*? *plan.* *?plan fasto:isCurrent “true”^^xsd:Boolean;* *fhir:DailyPerMealGlucoseLevel*? *goal.* *?goal fhir:Goal.target*? *target.* *?target fhir:Goal.target.detailQuantity*? *quant.* *?quant fhir:Quantity.code*? *code;* *fhir: Quantity.value*? *value;* *fhir: Quantity.system*? *system.* *}*	Q4. What is the current exercise plan for patient with identifier X.	*SELECT DISTINCT*? *exe WHERE {* *?p rdf:type fasto:patient;* *fhir:Resource.ID*? *id.* *?id fhir:Identifier.value X.* *?p fasto:hasPatientProfile*? *prof.* *?prof fasto:hasCarePlan*? *plan.* *?plan fasto:isCurrent “true”^^xsd:Boolean;* *fhir:CarePlan.activity*? *act.* *?act fhir:CarePlan.activity.reference*? *exe.* *?exe rdf:type fasto:exercisePlan.* *}*
Q5. Find all patients that are prevented from doing exercise with the compendium code of X.	*SELECT DISTINCT*? *p WHERE {* *?p rdf:type fasto:patient;* *fasto:hasPatientProfile*? *prof.* *?prof fasto:hasComplication*? *cond.* *?cond fhir:Condition.disease*? *d.* *?d fasto:diseaseContradictWithExercise*? *exe.* *?exe fasto:hasCompendiumCode X.* *}*	Q6. What is the insulin regimen defined for patient with identifier X.	*SELECT*? *ir WHERE {* *?p rdf:type fasto:patient;* *fhir:Resource.ID*? *id.* *?id fhir:Identifier.value X.* *?p fasto:hasPatientProfile*? *prof.* *?prof fasto:hasCarePlan*? *plan.* *?plan fasto:isCurrent “true”^^xsd:Boolean;* *fasto:hasInsulinRegimen*? *ir.* *}*
Q7. What are the current education materials assigned to the patient X.	*SELECT*? *rec,*? *topic,*? *course WHERE {* *?p rdf:type fasto:patient;* *fhir:Resource.ID*? *id.* *?id fhir:Identifier.value X.* *?p fasto:hasEducationRecord*? *rec.* *?rec fasto:hasLearningCourse*? *course;* *fasto:hasLearningTopic*? *topic.* *}*	Q8. Find intensive insulin regimens, which are based on the long acting insulin with SNOMED CT code of X. FASTO supports any standard coding terminology such as SNOMED CT, LOINC, RxNorm, ICD, etc. These standards support interoperability and improve the semantic meaning of medical terms.	*SELECT*? *ir WHERE {* *?ir rdf:type fasto:insulinRegimen;* *fasto:hasBasalInsulin*? *ba.* *?ba fhir:Medication.code*? *code;* *?code fhir:Coding*? *coding.* *?coding fhir:Coding.code X.* *}*
Q9. Find patients that are contradict with *414,518,007|insulin detemir*. A patient is prevented from taking insulin detemir in two cases: first, if he/she is currently taking a drug that contradicts with detemir, and second, if he/she has some diseases that is contradicting with detemir.	*SELECT DISTINCT*? *p WHERE {* *?p rdf:type fasto:patient;* *fasto:hasPatientProfile*? *prof.* *?prof fasto:hasPatientMedication*? *medstat.* *?medstat fhir:MedicationReference*? *med.* *?med fasto:drugContradictWithDrug*? *med2.* *?med2 fhir:Medication.code*? *s;* *fhir:Coding*? *coding.* *?coding fhir:Coding.code “126,212,009”^^xsd:string;* *fhir:Coding.system “SNOMEDCT”.* *}* *UNION* *{* *?p rdf:type fasto:patient;* *fasto:hasPatientProfile*? *prof.* *?prof fasto:hasComplication*? *cond.* *?cond fhir:Condition.disease*? *d.* *?d fasto:diseaseContradictWithDrug*? *med2.* *?med2 fhir:Medication.code*? *s;* *fhir:Coding*? *coding.* *?coding fhir:Coding.code “126,212,009”^^xsd:string;* *fhir:Coding.system “SNOMEDCT”.* *}*	Q10. List all patient taking rapid acting insulin with SNOMED CT code of X.	*SELECT DISTINCT*? *p WHERE {* *?p rdf:type fasto:patient;* *fasto:hasPatientProfile*? *prof.* *?prof fasto:hasCarePlan*? *plan.* *?plan fasto:isCurrent “true”^^xsd:Boolean;* *fasto:hasInsulinRegimen*? *ir.* *?ir fasto:hasBolusInsulin*? *ins.* *?ins fasto:Medication.code*? *code;* *?code fhir:Coding*? *coding;* *?coding fhir:Coding.code X.* *Fhir:Coding.system “SNOMEDCT”.* *}*
		Q11. Find child patients who have gotten in hypoglycemia before.	*SELECT DISTINCT*? *p WHERE {* *?p rdf:type fasto:patient;* *fhir:Person.age*? *a.* *FILTER (?a < 10).* *?p fasto:hasHistoryOfHypoglycemia*? *h.* *FILTER (?h > 0).* *}*
Q12. List all patient currently on fixed insulin regimen and suffered from hyperglycemia condition before.	*SELECT DISTINCT*? *p WHERE {* *?p rdf:type fasto:patient;* *fasto:hasPatientProfile*? *prof.* *fasto:hasHistoryOfHyperglycemia*? *i.* *FILTER (?i > 0)* *?prof fasto:hasCarePlan*? *plan.* *?plan fasto:isCurrent “true”^^xsd:Boolean;* *fasto:hasInsulinRegimen*? *ir.* *?ir rdf:type fasto:fixedRegimen;* *}*	Q13. List all patient who have a history of “increasing before bedtime insulin.” This pattern is inferred using the temporal abstraction of previous sensor data of before bedtime insulin. This abstraction is based on at least the previous 3 consecutive days.	*SELECT DISTINCT*? *p WHERE {* *?p rdf:type fasto:patient;* *fasto:hasPatientProfile*? *prof.* *?prof fasto:GlucoseBefBT “increasing”^^xsd:string* *}*
Q14. List all patient having the symptom of *shortness of breath*. Symptoms are encoded in SNOMED CT standard terminology.	*SELECT DISTINCT*? *p WHERE {* *?p rdf:type fasto:patient;* *fasto:hasPatientProfile*? *prof.* *?prof fasto:hasSymptom*? *sym.* *?sym fhir:Condition.code*? *cd.* *?cd fhir:Coding*? *cding;* *?cding fhir:Coding.code “267,036,007”^^xsd:string.* *Fhir:Coding.display “dyspnea”^^xsd:string.* *}*	Q15. What are the characteristics of WBAN of patient with identifier X.	*SELECT*? *wban,*? *sub,*? *lot,*? *date,*? *man,*? *mod WHERE {* *?p rdf:type fasto:patient;* *fhir:Resource.ID*? *id.* *?id fhir:Identifier.value X.* *?p fasto:hasWBAN*? *wban;* *?wban ssn:hasSubSystem*? *sub;* *?sub fhir:Device.lotNumber*? *lot;* *fhir:Device.manufactureDate*? *date;* *fhir:Device.manufacturer*? *man;* *fhir:Device.model*? *mod.* *}*
Q16. List the current sensor observations for patient X and their values.	*SELECT*? *obs*? *code*? *quan WHERE {* *?p rdf:type fasto:patient;* *fhir:Resource.ID*? *id.* *?id fhir:Identifier.value X.* *?p fasto:hasPatientProfile*? *prof.* *?prof fasto:hasObservationValue*? *obs.* *?obs rdf:type sensedObservationValue;* *fhir:Observation.code*? *code;* *fhir:Observation.valueQuantity*? *quan;* *}*	Q17. Find adult patients that are currently on *126,212,009|insulin glargine*.	*SELECT DISTINCT*? *p WHERE {* *?p rdf:type fasto:patient;* *fhir:Person.age*? *a;* *fasto:hasPatientProfile*? *prof.* *?prof fasto:hasPatientMedication*? *med.* *?med fhir:MedicationCodeableConcept*? *s.* *?s fhir:Coding*? *coding.* *?coding fhir:Coding.code “126,212,009”^^xsd:string;* *fhir:Coding.system “SNOMEDCT”;* *Fhir:Coding.display “insulin glargine”^^xsd:string.* *FILTER (?a > 19 &&*? *a < 55)* *}*

### Complete scenario

**Fig. 10:**
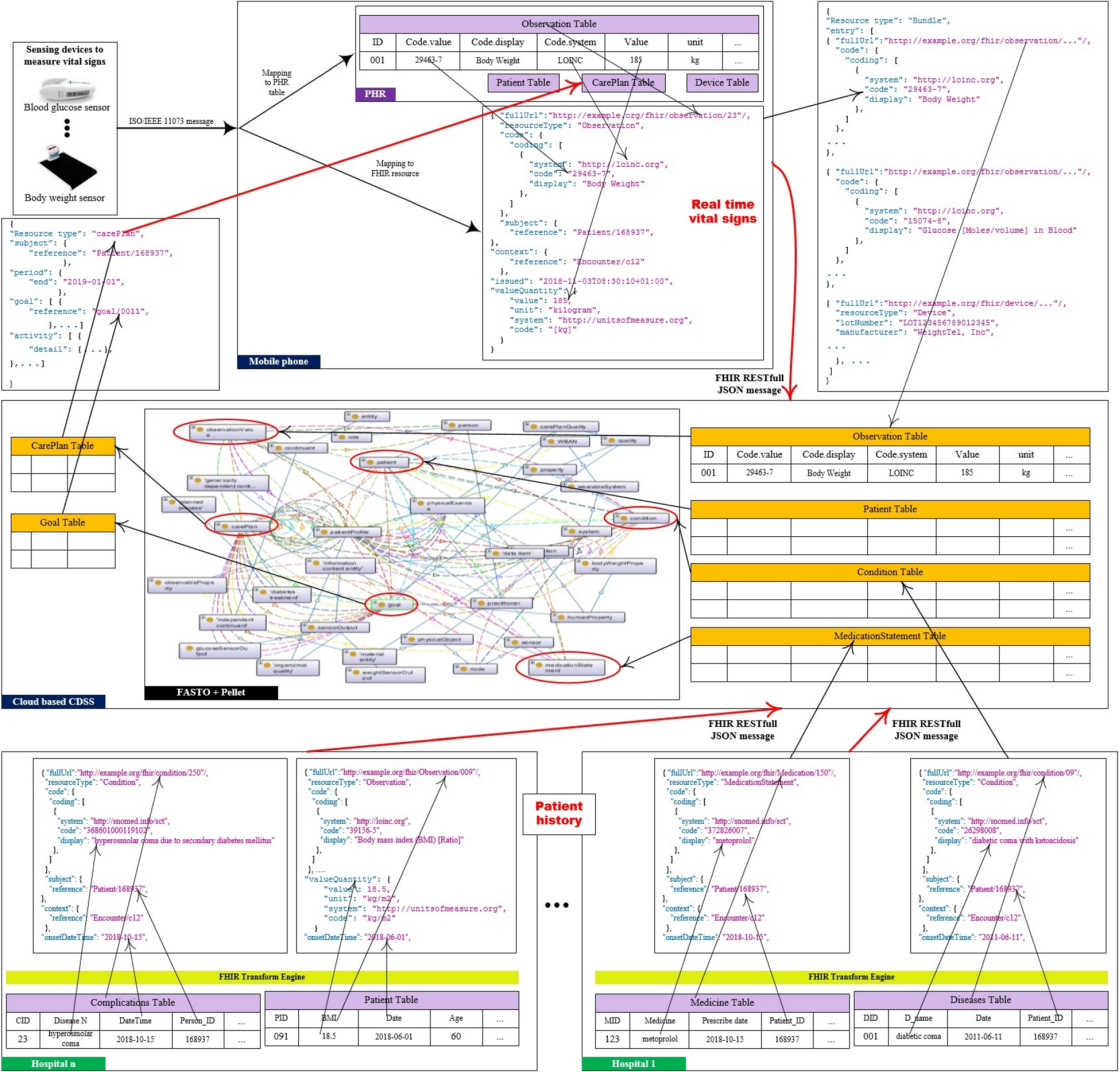
Complete scenario for the inference process of patient TPs and real time monitoring. This scenario starts and ends at the patient. According to the patient real time monitoring, these data are sent to the cloud based CDSS, which check the whole profile of the patient collected in a standard way from distributed EHR systems. After that, the CDSS proposes a personalized treatment plan

This section discusses one scenario inferring a patient’s TP and providing real-time monitoring, as shown in Fig. 10. In addition, this evaluation measures whether the ontology blends well with the rest of the system components, and if it interoperates with them seamlessly. A specific patient case is created by class instantiation, property assertions, and SWRL inferences based on the patient’s history received from EHRs and his/her current status received from sensors data.

#### Sensor data collection

Each sensor in the patient’s WBAN has its own reading frequency. For example, readings from the BG sensor are taken at least four times (before bedtime and three meals) per day, and the weight sensor takes one reading per day. The general format of the senor messages is< …  ∣ *message ID* ∣ *sensor ID* ∣ *time stamp* ∣ *value* ∣  … >. These messages are mapped to FHIR resources and collected in the PHR database as resource instances. For example, a new reading from the BG sensor is mapped to an observation resource format. These instances are periodically combined as FHIR bundle resource and sent in JSON format to the cloud. Please note, sensor data are converted to standard FHIR resources; in addition, the contents of the resources are coded using standard terminology, e.g. LOINC, and standard UoM, e.g. kg.

#### Data collection from EHR systems

The FHIR server in the cloud sends HTTP GET requests to the distributed EHR systems to collect the patient history as JSON-based resources. For example, the request “*GET* *http://fhirtest.com/Condition?patient=168937*” collects conditions of the patient with ID = 168,937 from the *fhirtest.com* server. These requests are received by FHIR servers in every hospital, which are responsible for preparing these resources from EHR systems. As a result, hospital systems are transparent to the CDSS.

EHR-based FHIR servers use the FHIR transform engine to map persistent EHR data to FHIR resources. All needed data for a CDSS (current drugs, diseases, allergies, symptoms, etc.) are requested from heterogeneous EHR systems. In Fig. 10, patient complications are collected as standard FHIR condition resources from two hospitals. For example, *patient p* has “*diabetic coma*” in *Hospital 1* and “*hyperosmolar coma*” in *Hospital n*. The collected resources are coded with standard terminologies, e.g. *26,298,008|diabetic coma with ketoacidosis* in SNOMED CT.

#### Cloud-based CDSS

**Fig. 11:**
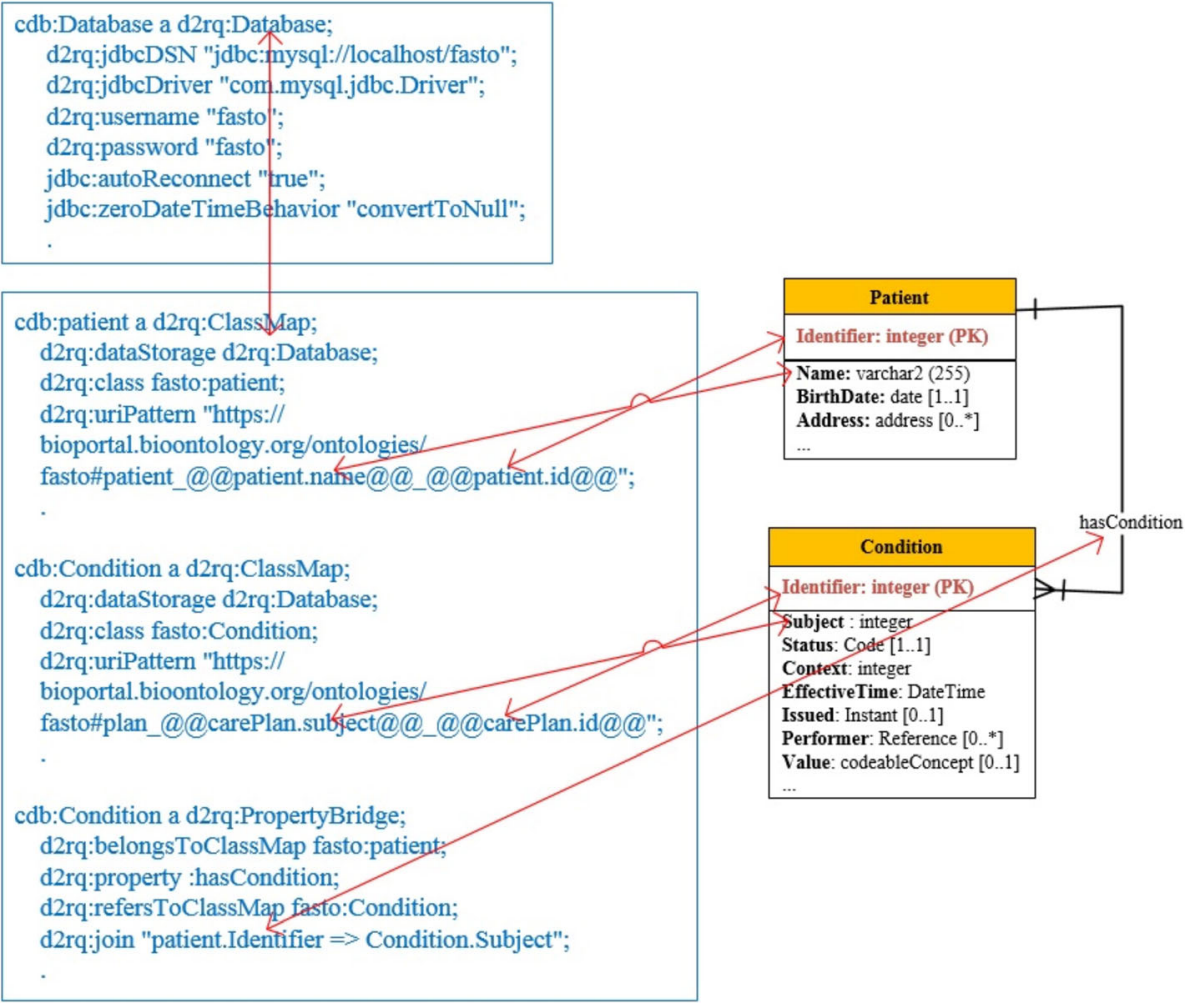
D2RQ examples for connecting to RDB and mapping a table, class, and property. This is a set of mappings among RDB tables, FASTO concepts, and FASTO properties. This mapping is based on D2RQ APIs

The patient profile is collected in the cloud and stored in the standard RDB (see Fig. 8). Mapping of collected FHIR resources to the RDB is a straightforward process because we used the same resource formats to design the database. In addition, RDB data are used to instantiate FASTO. To automate this process, we depend on the D2RQ Platform (*http://d2rq.org*). D2RQ and its D2RQ mapping language, a declarative language to map RDB schema to an OWL ontology, are used to export the patient profiles from RDB to RDF format. The RDB tables, relations, records, and constraints are mapped to FASTO classes, object properties, data properties, and axioms, respectively. Figure 11 shows an example of two class-mapping rules for the *patient* table to the *patient* class and the *condition* table to the *condition* class, and one property mapping of the *hasCondition* relationship to the *hasCondition* object property. Every mapping creates a new triple with a unique URL. These triples are asserted in FASTO ontology as instances or properties. For example, every observation is mapped to an SSN *observationValue* class, and every device is mapped to the *sensingDevice* class, etc.

FASTO represents all sensor data based on SSN semantics, standard terminologies as LOINC, and standard UoM. Furthermore, we represent all conditions, adverse events, symptoms, demographics, and drugs based on FHIR resources and standard terminologies, e.g. SNOMED CT. We added many other axioms to infer additional knowledge. For example, some axioms are used to infer contradictions between drugs, food, and diseases.

OWL 2 semantics enhance the inference capabilities of FASTO. For example, it can easily infer that the collected diseases in Fig. 10 from hospitals 1 and 2 can be interpreted as one complication. Now, the 140 SWRL rules are used to instantiate TPs for patients according to their profiles (see Fig. 7). These plans are instances of the carePlan class, which was designed based on the carePlan resource. The resulting carePlan objects and their associated *goal* objects are mapped to FHIR resources and sent to the patient mobile device, as shown in Fig. 10.

## Discussion

We propose an ontology-based mobile health CDSS for type 1 diabetes monitoring and treatment. The study provides a patient-centric comprehensive architecture based on a set of standards to handle interoperability challenges. There is a critical need for standard-: [1] data models for patient data representation, [2] approaches for CDSS knowledge formalization, [3] methods for data and knowledge sharing between distributed systems, [4] sources of medical knowledge, and [5] formats for sensor data representation. Ontology semantics and medical standards provide intelligent solutions to these needs.

Our previous studies demonstrated the benefits of using an ontology to build CDSSs [9, 10, 65, 66]. The formal and explicit semantics facilitate knowledge representation, sharing, and reuse. The instantiated ontology model together with a set of semantic web rule language (SWRL) rules constitute the CDSS knowledge base, which can be interpreted by inference engines such as Pellet. However, without consistent and globally accepted standard data models, the generated ontologies are incompatible with each other in *structure* and *semantics*, making it difficult for their integration, reuse, and maintenance. To handle the structure-consistency challenge, standard data models such as openEHR, HL7 v2 messages, and HL7 V3 reference information model (RIM), can be utilized to build standard ontologies [18]. Recently, HL7 proposed FHIR as an open standard, which concentrates on semantic interoperability [45–47, 49]. *To the best of our knowledge, no studies used FHIR to build standard ontologies, especially for diabetes* [15]. In addition, building ontologies based on a unified upper-level ontology (e.g. BFO, general formal ontology [GFO], and descriptive ontology for linguistic and cognitive engineering [DOLCE]) improves the interoperability and understandability of the resulting ontologies [9]. We employ BFO 2.0 to build our type 2 diabetes treatment ontology (DMTO) [10], but we did not use any standard data models. *No studies in the literature integrate BFO and FHIR to build a MH CDSS, especially for diabetes* [15]*. To handle the semantic consistency challenge*, the FHIR data model should be mapped to an OWL 2 ontology, and all of the ontology terminologies need to be bound with standard terminologies (e.g. systematized nomenclature of medicine – clinical terms [SNOMED CT], logical observation identifiers names and codes [LOINC], RxNorm, or the international classification of diseases [ICD]). Some of these terminologies have semantic problems, which can be solved by using more accurate description logic ontologies. We used OWL 2 ontology formalization to enhance SNOMED CT semantics [65]; *however, this type of integration has not been discussed in the literature*. CDSS medical knowledge can be collected from the results of machine learning algorithms, medical experts, and CPGs. Efficiency of machine learning algorithms is based on the quality of the input medical data, which is always low. In addition, it is difficult to collect heuristic knowledge from domain experts. Moreover, the significant gap between evidence-based medicine and clinical practice can result in lower quality and increased costs for medical care. As a result, building CDSS knowledge based on the most recent and standard CPGs is the best choice. *Finally*, ontologies should be used to improve the semantic representation of sensor data. The semantic enrichment of sensor data is called the semantic sensor web. The resulting ontology enhances the smooth integration of sensor data with historical EHR data. Furthermore, utilizing a standard sensor ontology such as the W3C’s SSN extends the interoperability between CDSSs and EHR ecosystems [57]. *To the best of our knowledge, utilizing SSN with formalized EHR to build MH CDSS systems has not been discussed in the literature* [15]*.* All of the previous challenges have been handled in the proposed study. We have concentrated mainly on the development of the core of component of CDSS system, namely its knowledge base. The resulting knowledge base is the FASTO ontology, which can be easily integrated with inference engine as Pellet reasoner. The most interesting part of the proposed system is the compatibility and interoperability of its modules, which facilitate the development of a transparent and pluggable CDSS system. At the same time, the proposed ontology can suggest a medically acceptable and complete care plans for diabetes patients.

To the best of our knowledge, this is the first complete MH infrastructure that handles the interoperability issue based on the available standards of SSN, BFO, SNOMED CT, FHIR, CPGs, etc. In addition, FASTO is the first public repository systematically documenting type 1 diabetes management. It creates individualized and customized treatment plans. These plans have many parts including insulin, lifestyle, and education that are created based on real time vital signs and historical EHR data (i.e., lab tests, complications, currently or previously taken drugs, symptoms, family history, etc.)

The proposed MH CDSS discussed in details the knowledge base development process and proposed comprehensive solutions for most of the implementation decisions. However, it still has some limitations. *First*, although FASTO is the most comprehensive type 1 DM treatment ontology, it did not handle some important treatment situations including emergencies. The limited availability of detailed medical knowledge in the literature is the main reason of this limitation. We studied most of the existing treatment CPGs and pathways; however, they did not provide a clear, comprehensive, and implementable knowledge about diabetes emergencies. FASTO has been implemented in a modular form. It is easy to extend and maintain its knowledge. As a result, it will stay open for any new or altered knowledge about diabetes medications. *Second*, FASTO models diet plans based on the grams of carbohydrates. This is according to the most recent CPGs; however, proteins and fats must have a clear role in diet plans. There is less knowledge about how to formulate the role of proteins and fats in meal planning. In addition, future enhancements are needed to tailor diet plans with familiar and preferred foods and with acceptable measurement units, such as cup, piece, etc. *Third*, FASTO provides treatment plans for type 1 diabetes only; however, a major step in managing diabetes is to manage its complications. *Fourth*, in the future, we will build the complete FASTO-based MH CDSS as an embedded component in an EHR system. This step will help us to put FASTO in a real environment; as a result, it will be easy to evaluate the performance of the proposed system and the quality of the proposed TPs.

## Conclusion

In this paper, we proposed a distributed, semantically intelligent, cloud-based, and interoperable MH CDSS framework. It can be used to provide monitoring of T1D patients. In addition, it can provide customized TPs according to the patient’s complete history and current vital signs. The proposed CDSS is based on the novel FASTO, which is a comprehensive OWL 2 ontology created by using Protégé 5.1 for T1D patients. The current version of FASTO includes 9577 classes, 658 object properties, 164 data properties, 460 individuals, and 140 SWRL rules. This is the first ontology that can provide complete and medically acceptable TPs based on historical EHRs and real time sensor readings. FASTO can be used to monitor BG in real time based on vital signs collected from WBANs. According to these real-time readings, FASTO suggests accurate adjustments in insulin dosages, eating patterns, and exercise plans. In addition, FASTO provides patients with tailored and long-term TPs with four main parts: insulin regimen, diet plan, exercise plan, and educational courses. The ontology has been tested, and it is publicly available through the BioPortal at *https://bioportal.bioontology.org/ontologies/FASTO*. We discussed the detailed process for creating this ontology, which provides semantic interoperability among CDSS knowledge, WBAN platforms, and distributed EHR systems. FASTO integrates a collection of standards to build a complete patient profile before making treatment decisions. FASTO is based on the BFO 2.0 top-level ontology, SSN ontology, HL7 FHIR standard, medical terminology, and T1D treatment CPGs. FASTO was designed in a modular manner, which makes it extensible and reusable in other domains.

One of the most important evaluation techniques of an ontology is by using applications. In the future, we will build a complete mobile health application for T1D monitoring. FASTO and an ontology reasoner will play the role of a CDSS. To handle the uncertain nature of medical data, we will extend our classic ontology into fuzzy ontology. We expect that fuzzy ontology will make the resulting system more acceptable and accurate. Finally, we will employ recent deep learning techniques, such as recurrent neural network, to help in pattern detection and management of patient sensor data. Pattern management helps to adjust a meal’s insulin, exercise’s insulin and carbs, and bedtime insulin. Finally, we will extend FASTO to deal with emergencies, such as hypoglycemia and hyperglycemia situations.

## Additional file

Additional file 1: The complete list of SWRL rules for type 1 diabetes mellitus treatment. This is a list of 140 SWRL rules that implement the semantics of the proposed CDSS. (DOCX 26 kb)

## Abbreviations

BFO: Basic Formal Ontology
BG: Blood glucose
CDA: Clinical document architecture
CDSS: Clinical Decision Support System
CGM: Continuous glucose monitoring
CPG: Clinical Practice Guideline
DDO: Diabetes Diagnosis Ontology
DMTO: Diabetes Mellitus Treatment Ontology
DOLCE: Descriptive ontology for linguistic and cognitive engineering
EHR: Electronic Health Record
FASTO: FHIR and SSN-based T1D Ontology
FHIR: Fast healthcare interoperability resources
GFO: General formal ontology
HL7: Health level seven
ICD: International classification of disease
ICR: Insulin-to-carbs ratio
IoT: Internet of Things
ISF: Insulin sensitivity factor
ISO TC: International organization for standardization technical committees
LOINC: Logical observation identifiers names and codes
MH: Mobile health
OAE: Ontology of adverse events
OOPS: OntOlogy pitfall scanner
OWL: Web Ontology Language
PHR: Personal health record
RDB: Relational database
SCT: SNOMED CT
SCTO: SNOMED CT standard ontology based on the ontology for general medical science
SNOMEDCT: Systematized Nomenclature of Medicine—Clinical Terms
SSN: Semantic sensor network
SWE: Sensor web enablement
SWRL: Semantic Web Rule Language
T1D: Type 1 diabetes mellitus
TP: Treatment plan
UMLS: Unified medical language system
UoM: Units of measurement
WBAN: Wireless body area network
WBU: Wireless base unit
