# Supplementary material for: A mobile health monitoring-and-treatment system based on integration of the SSN sensor ontology and the HL7 FHIR standard
Source: BMC Med Inform Decis Mak. 2019 May 10;19:97. doi: 10.1186/s12911-019-0806-z (PMC6511155; doi:10.1186/s12911-019-0806-z)
Supplement: Supplementary file 1 — The complete list of SWRL rules for type 1 diabetes mellitus treatment. This is a list of 140 SWRL rules that implement the semantics of the proposed CDSS. (DOCX 26 kb) [file 12911_2019_806_MOESM1_ESM.docx]

**Appendix 1: The complete list of SWRL rules for type 1 diabetes mellitus treatment**

1. patientContradictWithGlulisine(?p), patientContradictWithAspart(?p), patientProfile(?prof), hasPatientProfile(?p, ?prof), hasPreferedInsulinRegimen(?prof, "DP"^^xsd:string), carePlan(?cp), hasCarePlan(?prof, ?cp), FixedRegimen(?ir), hasInsulinRegimen(?cp, ?ir) -> hasFixedTwoshotsMorningShortActingInsulin(?ir, lispro), hasFixedTwoshotsEveningShortActingInsulin(?ir, lispro)
2. diseaseContradictWithDrug(?dis, ?m), hasPatientProfile(?p, ?prof), hasComplication(?prof, ?cond), patientProfile(?prof), disease(?dis), medication(?m), condition(?cond), patient(?p), Condition.disease(?cond, ?dis), glargine(?m) -> patientContradictWithGlargine(?p)
3. patient(?p), Person.age(?p, ?ag), hasValue(?ag, ?value), swrlb:greaterThan(?value, 19), swrlb:lessThan(?value, 55) -> adult(?p)
4. adolescent(?p), patientProfile(?prof), carePlan(?cp), goal(?bedTime_g), hasCarePlan(?prof, ?cp), CarePlan.goal(?cp, ?bedTime_g), Goal.target(?bedTime_g, ?bedTime_tar), goalTargetComponent(?bedTime_tar), Goal.target.detailRange(?bedTime_tar, ?bedTime_range), range(?bedTime_range), Range.low(?bedTime_range, ?bedTime_quant_low), quantity(?bedTime_quant_low), Quantity.value(?bedTime_quant_low, ?decim_bedTime_low), decimal(?decim_bedTime_low), Range.high(?bedTime_range, ?bedTime_quant_high), quantity(?bedTime_quant_high), Quantity.value(?bedTime_quant_high, ?decim_bedTime_high), decimal(?decim_bedTime_high) -> Quantity.code(?bedTime_quant_low, milligram_per_deciliter), Quantity.comparator(?bedTime_quant_low, comparator_code_greaterThanOrEqual), hasValue(?decim_bedTime_low, 90), Quantity.code(?bedTime_quant_high, milligram_per_deciliter), Quantity.comparator(?bedTime_quant_high, comparator_code_lessThanOrEqual), hasValue(?decim_bedTime_high, 150)
5. code(?cd), codeableConcept(?cc), coding(?code), string(?disp), string(?st), uri(?u), patient(?p), condition(?cond), patientProfile(?prof), CodeableConcept.coding(?cc, ?code), CodeableConcept.text(?cc, ?st), Coding.code(?code, ?cd), Coding.display(?code, ?disp), Coding.system(?code, ?uri), hasComplication(?prof, ?cond), hasPatientProfile(?p, ?prof), hasValue(?cd, "28960008"^^xsd:string), hasValue(?disp, "arteriosclerosis"), hasValue(?st, "arteriosclerosis"^^xsd:string), hasValue(?u, "http://snomed.info/sct"^^xsd:string), Condition.code(?cond, ?cc), Condition.disease(?cond, ?arteriosclerosis), arteriosclerosis(?arteriosclerosis) -> isInsulinResistant(?p, true)
6. patient(?p), patientProfile(?prof), hasPatientProfile(?p, ?prof), hasLifeStyle(?prof, "very active"^^xsd:string) -> hasActivityLevel(?prof, "1.725"^^xsd:double)
7. patient(?p), notForbiddenFromExercise(?p), isPragnant(?p, true), patientProfile(?prof), hasPatientProfile(?p, ?prof), hasRecommendedExercise(?prof, ?exe), 'aerobic exercise'(?exe), carePlan(?cp), CarePlan.status(?cp, carePlan_active), hasCarePlan(?prof, ?cp), CarePlan.activity(?cp, ?cp_a), carePlanActivityComponent(?cp_a), CarePlan.activity.reference(?cp_a, ?exe_p), exercisePlan(?exe_p), exercisePlan.haspart(?exe_p, ?exe_p_p), exercisePlanComponent(?exe_p_p), exercisePlan.component.activityDuration(?exe_p_p, ?exe_p_p_duration), range(?exe_p_p_duration), Range.low(?exe_p_p_duration, ?exe_p_p_duration_q_low), quantity(?exe_p_p_duration_q_low), Quantity.value(?exe_p_p_duration_q_low, ?exe_p_p_duration_q_low_v), decimal(?exe_p_p_duration_q_low_v), exercisePlan.component.totalWeeklyDuration(?exe_p_p, ?total_weekly), quantity(?total_weekly), Quantity.value(?total_weekly, ?total_weekly_value), decimal(?total_weekly_value), exercisePlan.component.activityFrequency(?exe_p_p, ?frequency), timing(?frequency), Timing.repeat.boundsRange(?frequency, ?frequency_range), range(?frequency_range), Range.low(?frequency_range, ?frequency_range_low_q), quantity(?frequency_range_low_q), Quantity.value(?frequency_range_low_q, ?frequency_range_low_q_value) -> exercisePlan.subject(?exe_p, ?p), exercisePlan.isPartOf(?exe_p, ?cp), Quantity.code(?exe_p_p_duration_q_low, minutes), Quantity.system(?exe_p_p_duration_q_low, UCUM_coding_system), Quantity.comparator(?exe_p_p_duration_q_low, comparator_code_lessThanOrEqual), hasValue(?exe_p_p_duration_q_low_v, 30), exercisePlan.component.intensity(?exe_p_p, intensity_level_moderate), hasValue(?total_weekly_value, 90), Quantity.code(?total_weekly, minutes), Quantity.system(?total_weekly, UCUM_coding_system), Quantity.comparator(?total_weekly, comparator_code_greaterThanOrEqual), Quantity.code(?frequency_range_low_q, days_per_week), Quantity.comparator(?frequency_range_low_q, comparator_code_equal), hasValue(?frequency_range_low_q_value, 3), exercisePlan.component.exerciseType(?exe_p_p, ?exe)
8. child(?p), patientProfile(?prof), carePlan(?cp), goal(?bedTime_g), hasCarePlan(?prof, ?cp), CarePlan.goal(?cp, ?bedTime_g), Goal.target(?bedTime_g, ?bedTime_tar), goalTargetComponent(?bedTime_tar), Goal.target.detailRange(?bedTime_tar, ?bedTime_range), range(?bedTime_range), Range.low(?bedTime_range, ?bedTime_quant_low), quantity(?bedTime_quant_low), Quantity.value(?bedTime_quant_low, ?decim_bedTime_low), decimal(?decim_bedTime_low), Range.high(?bedTime_range, ?bedTime_quant_high), quantity(?bedTime_quant_high), Quantity.value(?bedTime_quant_high, ?decim_bedTime_high), decimal(?decim_bedTime_high) -> Quantity.code(?bedTime_quant_low, milligram_per_deciliter), Quantity.comparator(?bedTime_quant_low, comparator_code_greaterThanOrEqual), hasValue(?decim_bedTime_low, 90), Quantity.code(?bedTime_quant_high, milligram_per_deciliter), Quantity.comparator(?bedTime_quant_high, comparator_code_lessThanOrEqual), hasValue(?decim_bedTime_high, 150)
9. oldAdult(?p), patientProfile(?prof), carePlan(?cp), goal(?a1c_g), hasCarePlan(?prof, ?cp), CarePlan.HbA1C.goal(?cp, ?a1c_g), Goal.target(?a1c_g, ?a1c_tar), goalTargetComponent(?a1c_tar), Goal.target.detailQuantity(?a1c_tar, ?a1c_quant), quantity(?a1c_quant), Quantity.value(?a1c_quant, ?decim), decimal(?decim) -> Quantity.code(?a1c_quant, percent), hasValue(?decim, 7.5), Quantity.comparator(?a1c_quant, comparator_code_lessThan)
10. adult(?p), notForbiddenFromExercise(?p), Person.age(?p, ?age), decimal(?age), hasValue(?age, ?age_value), swrlb:greaterThan(?age_value, 17), patientProfile(?prof), hasPatientProfile(?p, ?prof), hasRecommendedExercise(?prof, ?exe), carePlan(?cp), CarePlan.status(?cp, carePlan_active), hasCarePlan(?prof, ?cp), CarePlan.activity(?cp, ?cp_a), carePlanActivityComponent(?cp_a), CarePlan.activity.reference(?cp_a, ?exe_p), exercisePlan(?exe_p), exercisePlan.haspart(?exe_p, ?exe_p_p), exercisePlanComponent(?exe_p_p), exercisePlan.component.activityDuration(?exe_p_p, ?exe_p_p_duration), range(?exe_p_p_duration), Range.low(?exe_p_p_duration, ?exe_p_p_duration_q_low), quantity(?exe_p_p_duration_q_low), Quantity.value(?exe_p_p_duration_q_low, ?exe_p_p_duration_q_low_v), decimal(?exe_p_p_duration_q_low_v), exercisePlan.component.totalWeeklyDuration(?exe_p_p, ?total_weekly), quantity(?total_weekly), Quantity.value(?total_weekly, ?total_weekly_value), decimal(?total_weekly_value), exercisePlan.component.activityFrequency(?exe_p_p, ?frequency), timing(?frequency), Timing.repeat.boundsRange(?frequency, ?frequency_range), range(?frequency_range), Range.low(?frequency_range, ?frequency_range_low_q), quantity(?frequency_range_low_q), Quantity.value(?frequency_range_low_q, ?frequency_range_low_q_value) -> exercisePlan.subject(?exe_p, ?p), exercisePlan.isPartOf(?exe_p, ?cp), Quantity.code(?exe_p_p_duration_q_low, minutes), Quantity.system(?exe_p_p_duration_q_low, UCUM_coding_system), Quantity.comparator(?exe_p_p_duration_q_low, comparator_code_greaterThanOrEqual), hasValue(?exe_p_p_duration_q_low_v, 150), exercisePlan.component.intensity(?exe_p_p, intensity_level_moderate_to_severe), hasValue(?total_weekly_value, 450), Quantity.code(?total_weekly, minutes), Quantity.system(?total_weekly, UCUM_coding_system), Quantity.comparator(?total_weekly, comparator_code_greaterThanOrEqual), Quantity.code(?frequency_range_low_q, days_per_week), Quantity.comparator(?frequency_range_low_q, comparator_code_greaterThanOrEqual), hasValue(?frequency_range_low_q_value, 3), exercisePlan.component.exerciseType(?exe_p_p, ?exe)
11. patient(?p), patientProfile(?prof), hasPatientProfile(?p, ?prof), hasComplication(?prof, ?c), condition(?c), Condition.disease(?c, ?dis), 'diabetic gangrene'(?dis) -> forbiddenFromExercise(?p)
12. patient(?p), patientProfile(?prof), hasPatientProfile(?p, ?prof), hasPreferedInsulinRegimen(?prof, "DP"^^xsd:string), carePlan(?cp), hasCarePlan(?prof, ?cp), FixedRegimen(?ir), hasInsulinRegimen(?cp, ?ir), hasTotalDailyDose(?ir, ?tdd), dosage(?e_s), hasFixedTwoshotsEveningShortActingInsulinDose(?ir, ?e_s), Dosage.doseSimpleQuantity(?e_s, ?e_s_quant), quantity(?e_s_quant), Quantity.value(?e_s_quant, ?e_s_quant_decim), swrlb:divide(?e_factor_s, 1, 6), swrlb:multiply(?e_s_quant_decim_value, ?e_factor_s, ?tdd), swrlb:ceiling(?fin, ?e_s_quant_decim_value) -> hasValue(?e_s_quant_decim, ?fin)
13. patient(?p), hasBreakfastMeal(?p, ?bm), meal(?bm), hasDinnerMeal(?p, ?dm), meal(?dm), hasLunchMeal(?p, ?lm), meal(?lm), patientProfile(?prof), hasPatientProfile(?p, ?prof), carePlan(?cp), hasCarePlan(?prof, ?cp), CarePlan.activity(?cp, ?act), carePlanActivityComponent(?act), CarePlan.activity.reference(?act, ?nu_order), nutritionOrder(?nu_order), NutritionOrder.dailyCalories(?nu_order, ?nu_order_quant), quantity(?nu_order_quant), Quantity.value(?nu_order_quant, ?nu_order_quant_value_1), decimal(?nu_order_quant_value_1), hasValue(?nu_order_quant_value_1, ?nu_order_quant_value), swrlb:multiply(?breakfast_cal, ?nu_order_quant_value, 0.3), swrlb:ceiling(?final_breakfast_cal, ?breakfast_cal), swrlb:multiply(?lunch_cal, ?nu_order_quant_value, 0.35), swrlb:ceiling(?final_lunch_cal, ?lunch_cal), swrlb:multiply(?dinner_cal, ?nu_order_quant_value, 0.35), swrlb:ceiling(?final_dinner_cal, ?dinner_cal), swrlb:divide(?total_carbs, ?nu_order_quant_value, 4), swrlb:multiply(?breakfast_carbs, ?total_carbs, 0.3), swrlb:ceiling(?final_breakfast_carbs, ?breakfast_carbs), swrlb:multiply(?lunch_carbs, ?total_carbs, 0.35), swrlb:ceiling(?final_lunch_carbs, ?lunch_carbs), swrlb:multiply(?dinner_carbs, ?total_carbs, 0.35), swrlb:ceiling(?final_dinner_carbs, ?dinner_carbs) -> Meal.totalCalories(?bm, ?final_breakfast_cal), Meal.totalCalories(?lm, ?final_lunch_cal), Meal.totalCalories(?dm, ?final_dinner_cal), Meal.totalCarbsInGrams(?bm, ?final_breakfast_carbs), Meal.totalCarbsInGrams(?lm, ?final_lunch_carbs), Meal.totalCarbsInGrams(?dm, ?final_dinner_carbs), NutritionOrder.meal(?nu_order, ?bm), NutritionOrder.meal(?nu_order, ?lm), NutritionOrder.meal(?nu_order, ?dm)
14. oldAdult(?p), patientProfile(?prof), carePlan(?cp), goal(?bedTime_g), hasCarePlan(?prof, ?cp), CarePlan.goal(?cp, ?bedTime_g), Goal.target(?bedTime_g, ?bedTime_tar), goalTargetComponent(?bedTime_tar), Goal.target.detailRange(?bedTime_tar, ?bedTime_range), range(?bedTime_range), Range.low(?bedTime_range, ?bedTime_quant_low), quantity(?bedTime_quant_low), Quantity.value(?bedTime_quant_low, ?decim_bedTime_low), decimal(?decim_bedTime_low), Range.high(?bedTime_range, ?bedTime_quant_high), quantity(?bedTime_quant_high), Quantity.value(?bedTime_quant_high, ?decim_bedTime_high), decimal(?decim_bedTime_high) -> Quantity.code(?bedTime_quant_low, milligram_per_deciliter), Quantity.comparator(?bedTime_quant_low, comparator_code_greaterThanOrEqual), hasValue(?decim_bedTime_low, 90), Quantity.code(?bedTime_quant_high, milligram_per_deciliter), Quantity.comparator(?bedTime_quant_high, comparator_code_lessThanOrEqual), hasValue(?decim_bedTime_high, 150)
15. patient(?p), patientProfile(?prof), hasPatientProfile(?p, ?prof), hasLifeStyle(?prof, "extra active"^^xsd:string) -> hasActivityLevel(?prof, "1.9"^^xsd:double)
16. patient(?p), patientProfile(?prof), hasPatientProfile(?p, ?prof), hasObservationValue(?prof, ?breakfastBGObs), glucoseLevelValue(?breakfastBGObs), isCurrent(?breakfastBGObs, true), Observation.valueQuantity(?breakfastBGObs, ?breakfastBGObs_value), quantity(?breakfastBGObs_value), Quantity.value(?breakfastBGObs_value, ?breakfastBGObs_value_dec), Quantity.code(?breakfastBGObs_value, milligram_per_deciliter), hasValue(?breakfastBGObs_value_dec, ?cbg), swrlb:greaterThan(?cbg, 250) -> forbiddenFromExercise(?p)
17. patientContradictWithDetemir(?p), patientProfile(?prof), hasPatientProfile(?p, ?prof), hasPreferedInsulinRegimen(?prof, "IIT"^^xsd:string), carePlan(?cp), hasCarePlan(?prof, ?cp), intensiveInsulinTherapy(?ir), hasInsulinRegimen(?cp, ?ir) -> hasBasalInsulin(?ir, lantus)
18. patient(?p), patientProfile(?prof), hasPatientProfile(?p, ?prof), hasGlucoseBehaviorBeforeBreakfast(?prof, "decreasing"^^xsd:string), carePlan(?cp), isCurrent(?cp, true), CarePlan.status(?cp, carePlan_active), hasCarePlan(?prof, ?cp), hasInsulinRegimen(?cp, ?ir), intensiveInsulinTherapy(?ir), hasBasalInsulinMorningAdjustment(?ir, ?ba), basalInsulinAdjustment(?ba), hasQuantityInUnits(?ba, ?ba_q), quantity(?ba_q), Quantity.value(?ba_q, ?ba_q_v), decimal(?ba_q_v) -> isCurrent(?ba, true), basalInsulinAdjustType(?ba, "+"^^xsd:string), Quantity.code(?ba_q, percent), Quantity.comparator(?ba_q, comparator_code_equal), Quantity.system(?ba_q, UCUM_coding_system), hasValue(?ba_q_v, 10.0)
19. child(?p), notForbiddenFromExercise(?p), patientProfile(?prof), hasPatientProfile(?p, ?prof), hasRecommendedExercise(?prof, ?exe), 'aerobic exercise'(?exe), carePlan(?cp), CarePlan.status(?cp, carePlan_active), hasCarePlan(?prof, ?cp), CarePlan.activity(?cp, ?cp_a), carePlanActivityComponent(?cp_a), CarePlan.activity.reference(?cp_a, ?exe_p), exercisePlan(?exe_p), exercisePlan.haspart(?exe_p, ?exe_p_p), exercisePlanComponent(?exe_p_p), exercisePlan.component.activityDuration(?exe_p_p, ?exe_p_p_duration), range(?exe_p_p_duration), Range.low(?exe_p_p_duration, ?exe_p_p_duration_q_low), quantity(?exe_p_p_duration_q_low), Quantity.value(?exe_p_p_duration_q_low, ?exe_p_p_duration_q_low_v), decimal(?exe_p_p_duration_q_low_v), exercisePlan.component.totalWeeklyDuration(?exe_p_p, ?total_weekly), quantity(?total_weekly), Quantity.value(?total_weekly, ?total_weekly_value), decimal(?total_weekly_value), exercisePlan.component.activityFrequency(?exe_p_p, ?frequency), timing(?frequency), Timing.repeat.boundsRange(?frequency, ?frequency_range), range(?frequency_range), Range.low(?frequency_range, ?frequency_range_low_q), quantity(?frequency_range_low_q), Quantity.value(?frequency_range_low_q, ?frequency_range_low_q_value) -> exercisePlan.subject(?exe_p, ?p), exercisePlan.isPartOf(?exe_p, ?cp), Quantity.code(?exe_p_p_duration_q_low, minutes), Quantity.system(?exe_p_p_duration_q_low, UCUM_coding_system), Quantity.comparator(?exe_p_p_duration_q_low, comparator_code_greaterThanOrEqual), hasValue(?exe_p_p_duration_q_low_v, 60), exercisePlan.component.intensity(?exe_p_p, intensity_level_moderate_to_severe), hasValue(?total_weekly_value, 180), Quantity.code(?total_weekly, minutes), Quantity.system(?total_weekly, UCUM_coding_system), Quantity.comparator(?total_weekly, comparator_code_greaterThanOrEqual), Quantity.code(?frequency_range_low_q, days_per_week), Quantity.comparator(?frequency_range_low_q, comparator_code_greaterThanOrEqual), hasValue(?frequency_range_low_q_value, 3), exercisePlan.component.exerciseType(?exe_p_p, ?exe)
20. hasCarePlan(?prof, ?cp), hasPreferedInsulinRegimen(?prof, "IIT"^^xsd:string), hasInsulinRegimen(?cp, ?ir), swrlb:divide(?pre_final, ?f, 3), hasPatientProfile(?p, ?prof), Coding.display(?breakfast_dosage_route_coding, ?breakfast_dosage_route_coding_display), intensiveInsulinTherapy(?ir), dosage(?breakfast_dosage), CodeableConcept.coding(?breakfast_dosage_route, ?breakfast_dosage_route_coding), CodeableConcept.coding(?breakfast_dosage_timing_codeable, ?breakfast_dosage_timing_codeable_coding), quantity(?breakfast_dosage_quant), codeableConcept(?breakfast_dosage_route), Quantity.value(?breakfast_dosage_quant, ?breakfast_dosage_quant_decimal), hasBolusBreakfastDose(?ir, ?breakfast_dosage), Timing.code(?breakfast_dosage_timing, ?breakfast_dosage_timing_codeable), Dosage.doseSimpleQuantity(?breakfast_dosage, ?breakfast_dosage_quant), hasBolusInsulinUnits(?ir, ?f), Coding.code(?breakfast_dosage_route_coding, ?breakfast_dosage_route_coding_code), Coding.code(?breakfast_dosage_timing_codeable_coding, ?breakfast_dosage_timing_codeable_coding_code), timing(?breakfast_dosage_timing), swrlb:ceiling(?final, ?pre_final), patientProfile(?prof), patient(?p), carePlan(?cp), Dosage.route(?breakfast_dosage, ?breakfast_dosage_route), Dosage.timing(?breakfast_dosage, ?breakfast_dosage_timing), codeableConcept(?breakfast_dosage_timing_codeable), coding(?breakfast_dosage_route_coding), Coding.display(?breakfast_dosage_timing_codeable_coding, ?breakfast_dosage_timing_codeable_coding_display) -> hasValue(?breakfast_dosage_quant_decimal, ?final), Coding.system(?breakfast_dosage_timing_codeable_coding, SNOMED_CT), hasValue(?breakfast_dosage_timing_codeable_coding_code, "1751000175104"^^xsd:string), hasValue(?breakfast_dosage_route_coding_code, "59108006"^^xsd:string), Quantity.code(?breakfast_dosage_quant, units), Coding.system(?breakfast_dosage_route_coding, SNOMED_CT), Quantity.system(?breakfast_dosage_quant, UCUM_coding_system), hasValue(?breakfast_dosage_route_coding_display, "injection"^^xsd:string), hasValue(?breakfast_dosage_timing_codeable_coding_display, "daily with breakfast"^^xsd:string)
21. patient(?p), hasPreferedBGUoM(?p, "mmol"^^xsd:string), patientProfile(?prof), hasPatientProfile(?p, ?prof), carePlan(?cp), hasCarePlan(?prof, ?cp), CarePlan.status(?cp, carePlan_active), hasInsulinRegimen(?cp, ?ir), insulinRegimen(?ir), hasTotalDailyDose(?ir, ?tdd), hasInsulinSensitivityFactor(?prof, ?isf), quantity(?isf), swrlb:divide(?result, 100, ?tdd), Quantity.value(?isf, ?isf_v), decimal(?isf_v) -> Quantity.code(?isf, millimoles_per_litre_per_unit), Quantity.comparator(?isf, comparator_code_equal), Quantity.value(?isf_v, ?result)
22. adolescent(?p), educationalAchievement(?p, "lowEducation"^^xsd:string), hasEducationRecord(?p, ?er), visual(?v), games(?g), reading(?red) -> hasLearningStyle(?er, ?v), hasLearningStyle(?er, ?g), hasLearningStyle(?er, ?red)
23. code(?cd), codeableConcept(?cc), coding(?code), string(?disp), string(?st), uri(?u), patient(?p), condition(?cond), patientProfile(?prof), CodeableConcept.coding(?cc, ?code), CodeableConcept.text(?cc, ?st), Coding.code(?code, ?cd), Coding.display(?code, ?disp), Coding.system(?code, ?uri), hasComplication(?prof, ?cond), hasPatientProfile(?p, ?prof), Condition.code(?cond, ?cc), hasValue(?cd, "402599005"^^xsd:string), hasValue(?disp, "acanthosis nigricans"), hasValue(?st, "acanthosis nigricans"^^xsd:string), hasValue(?u, "http://snomed.info/sct"^^xsd:string), 'acanthosis nigricans'(?aca), Condition.disease(?cond, ?aca) -> isInsulinResistant(?p, true)
24. adolescent(?p), patientProfile(?prof), carePlan(?cp), goal(?preMeal_g), hasCarePlan(?prof, ?cp), CarePlan.dailyPerMealGlucoseLevel.goal(?cp, ?preMeal_g), Goal.target(?preMeal_g, ?preMeal_tar), goalTargetComponent(?preMeal_tar), Goal.target.detailRange(?preMeal_tar, ?preMeal_range), range(?preMeal_range), Range.low(?preMeal_range, ?preMeal_quant_low), quantity(?preMeal_quant_low), Quantity.value(?preMeal_quant_low, ?decim_preMeal_low), decimal(?decim_preMeal_low), Range.high(?preMeal_range, ?preMeal_quant_high), quantity(?preMeal_quant_high), Quantity.value(?preMeal_quant_high, ?decim_preMeal_high), decimal(?decim_preMeal_high) -> Quantity.code(?preMeal_quant_low, milligram_per_deciliter), hasValue(?decim_preMeal_low, 90), Quantity.comparator(?preMeal_quant_low, comparator_code_greaterThanOrEqual), Quantity.code(?preMeal_quant_high, milligram_per_deciliter), hasValue(?decim_preMeal_high, 150), Quantity.comparator(?preMeal_quant_high, comparator_code_lessThanOrEqual)
25. patient(?p), Person.age(?p, ?ag), hasValue(?ag, ?value), swrlb:lessThan(?value, 10) -> child(?p)
26. oldAdult(?p), patientProfile(?prof), carePlan(?cp), goal(?preMeal_g), hasCarePlan(?prof, ?cp), CarePlan.dailyPerMealGlucoseLevel.goal(?cp, ?preMeal_g), Goal.target(?preMeal_g, ?preMeal_tar), goalTargetComponent(?preMeal_tar), Goal.target.detailRange(?preMeal_tar, ?preMeal_range), range(?preMeal_range), Range.low(?preMeal_range, ?preMeal_quant_low), quantity(?preMeal_quant_low), Quantity.value(?preMeal_quant_low, ?decim_preMeal_low), decimal(?decim_preMeal_low), Range.high(?preMeal_range, ?preMeal_quant_high), quantity(?preMeal_quant_high), Quantity.value(?preMeal_quant_high, ?decim_preMeal_high), decimal(?decim_preMeal_high) -> Quantity.code(?preMeal_quant_low, milligram_per_deciliter), hasValue(?decim_preMeal_low, 90), Quantity.comparator(?preMeal_quant_low, comparator_code_greaterThanOrEqual), Quantity.code(?preMeal_quant_high, milligram_per_deciliter), hasValue(?decim_preMeal_high, 130), Quantity.comparator(?preMeal_quant_high, comparator_code_lessThanOrEqual)
27. isCurrent(?w, true), hasCarePlan(?prof, ?cp), nutritionOrderSupplementComponent(?supp), hasPatientProfile(?p, ?prof), Quantity.value(?quant1, ?val1), Quantity.value(?quant3, ?val3), swrlb:multiply(?h_v, ?value3, 4.7), NutritionOrder.supplement.type(?supp, ?supp_type), swrlb:subtract(?w_h_a, ?w_h, ?a_v), hasObservationValue(?prof, ?h), Coding.display(?supp_type_coding, ?supp_type_coding_display), Quantity.code(?quant1, pound), isCurrent(?h, true), quantity(?supp_quant), NutritionOrder.supplement.quantity(?supp, ?supp_quant), Quantity.code(?quant3, inch), hasValue(?val1, ?value1), hasValue(?val3, ?value3), Person.age(?p, ?ag), weightValue(?w), Quantity.value(?supp_quant, ?supp_quant_val), quantity(?quant1), Observation.valueQuantity(?w, ?quant1), quantity(?quant3), decimal(?supp_quant_val), CodeableConcept.coding(?supp_type, ?supp_type_coding), Coding.code(?supp_type_coding, ?supp_type_coding_code), swrlb:ceiling(?final, ?pre_final), swrlb:multiply(?a_v, ?value2, 4.7), CarePlan.activity.reference(?act, ?nu_order), codeableConcept(?supp_type), patientProfile(?prof), patient(?p), carePlanActivityComponent(?act), Observation.valueQuantity(?h, ?quant3), swrlb:multiply(?w_v, ?value1, 4.35), swrlb:add(?pre_final, ?w_h_a, 655.1), NutritionOrder.supplement(?nu_order, ?supp), hasObservationValue(?prof, ?w), CarePlan.activity(?cp, ?act), Person.gender(?p, gender_female), coding(?supp_type_coding), height(?h), swrlb:add(?w_h, ?w_v, ?h_v), nutritionOrder(?nu_order), hasValue(?ag, ?value2), decimal(?val3), decimal(?val1) -> hasValue(?supp_quant_val, ?final), Quantity.system(?supp_quant, UCUM_coding_system), hasValue(?supp_type_coding_display, "basal metabolic rate (observable entity)"^^xsd:string), Coding.system(?supp_type_coding, SNOMED_CT), Quantity.code(?supp_quant, kilocalorie), hasValue(?supp_type_coding_code, "165109007"^^xsd:string)
28. patient(?p), patientProfile(?prof), (hasComplication exactly 0 condition)(?prof), hasPatientProfile(?p, ?prof) -> isInsulinResistant(?p, false)
29. notForbiddenFromExercise(?p), patientProfile(?prof), hasPatientProfile(?p, ?prof), hasComplication(?prof, ?c), condition(?c), Condition.disease(?c, ?dis), diseaseContradictWithExercise(?dis, ?exe), physicalExercise(?exe) -> hasForbiddenExercise(?prof, ?exe)
30. adolescent(?p), patientProfile(?prof), carePlan(?cp), goal(?a1c_g), hasCarePlan(?prof, ?cp), CarePlan.HbA1C.goal(?cp, ?a1c_g), Goal.target(?a1c_g, ?a1c_tar), goalTargetComponent(?a1c_tar), Goal.target.detailQuantity(?a1c_tar, ?a1c_quant), quantity(?a1c_quant), Quantity.value(?a1c_quant, ?decim), decimal(?decim) -> Quantity.code(?a1c_quant, percent), hasValue(?decim, 7.5), Quantity.comparator(?a1c_quant, comparator_code_lessThan)
31. patient(?p), patientProfile(?prof), hasPatientProfile(?p, ?prof), medicationStatement(?ms), hasPatientMedication(?prof, ?ms), medication(?m), MedicationStatement.medicationReference(?ms, ?m), drugContradictWithDrug(?m, ?m2), detemir(?m2) -> patientContradictWithDetemir(?p)
32. notForbiddenFromExercise(?p), patientProfile(?prof), hasPatientProfile(?p, ?prof), physicalExercise(?exe1), physicalExercise(?exe2), hasPreferredExercise(?prof, ?exe1), hasForbiddenExercise(?prof, ?exe2), DifferentFrom (?exe1, ?exe2) -> hasRecommendedExercise(?prof, ?exe1)
33. patient(?p), patientProfile(?prof), hasPatientProfile(?p, ?prof), hasInsulinSensitivityFactor(?prof, ?isf), quantity(?isf), Quantity.value(?isf, ?isf_dec), hasValue(?isf_dec, ?isf_v), hasInsulinToCarbohydrateRatio(?prof, ?icr), quantity(?icr), Quantity.value(?icr, ?icr_dec), hasValue(?icr_dec, ?icr_v), hasObservationValue(?prof, ?breakfastBGObs), breakfastGlucoseObservationValue(?breakfastBGObs), isCurrent(?breakfastBGObs, true), Observation.valueQuantity(?breakfastBGObs, ?breakfastBGObs_value), quantity(?breakfastBGObs_value), Quantity.value(?breakfastBGObs_value, ?breakfastBGObs_value_dec), Quantity.code(?breakfastBGObs_value, milligram_per_deciliter), hasValue(?breakfastBGObs_value_dec, ?cbg), hasObservationValue(?prof, ?weight), weightValue(?weight), Observation.valueQuantity(?weight, ?weight_quant), quantity(?weight_quant), Quantity.code(?weight_quant, kilogram), Quantity.value(?weight_quant, ?weight_quant_value), hasValue(?weight_quant_value, ?w), swrlb:multiply(?pre_bc, ?met, ?w), swrlb:multiply(?bc, ?pre_bc, ?hours), hasObservationValue(?prof, ?neededCarbs), neededToEatCarbs(?neededCarbs), isCurrent(?neededCarbs, true), Observation.valueQuantity(?neededCarbs, ?neededCarbs_value), quantity(?neededCarbs_value), Quantity.value(?neededCarbs_value, ?neededCarbs_value_dec), Quantity.code(?neededCarbs_value, gram), hasValue(?neededCarbs_value_dec, ?mc), hasObservationValue(?prof, ?exercise), exerciseByMET(?exercise), isCurrent(?exercise, true), Observation.component(?exercise, ?exercise_comp), ObservationComponentComponent(?exercise_comp), Observation.component.code(?exercise_comp, ?exercise_comp_codeable), CodeableConcept.coding(?exercise_comp_codeable, ?exercise_comp_codeable_coding), coding(?exercise_comp_codeable_coding), Coding.code(?exercise_comp_codeable_coding, ?exercise_comp_codeable_coding_code), Coding.system(?exercise_comp_codeable_coding, SNOMED_CT), hasValue(?exercise_comp_codeable_coding_code, "698834005"^^xsd:string), Observation.component.valueQuantity(?exercise_comp, ?exercise_comp_quant), quantity(?exercise_comp_quant), Quantity.value(?exercise_comp_quant, ?exercise_comp_quant_value), hasValue(?exercise_comp_quant_value, ?met), Observation.component(?exercise, ?exercise_comp_dur), ObservationComponentComponent(?exercise_comp_dur), Observation.component.code(?exercise_comp_dur, ?exercise_comp_dur_codeable), CodeableConcept.coding(?exercise_comp_dur_codeable, ?eexercise_comp_dur_codeable_coding), coding(?exercise_comp_dur_codeable_coding), Coding.code(?exercise_comp_dur_codeable_coding, ?exercise_comp_dur_codeable_coding_code), Coding.system(?exercise_comp_dur_codeable_coding, SNOMED_CT), hasValue(?exercise_comp_dur_codeable_coding_code, "103335007"^^xsd:string), Observation.component.valueQuantity(?exercise_comp_dur, ?exercise_comp_dur_quant), quantity(?exercise_comp_dur_quant), Quantity.value(?exercise_comp_dur_quant, ?exercise_comp_dur_quant_value), hasValue(?exercise_comp_dur_quant_value, ?hours), hasCarePlan(?prof, ?cp), carePlan(?cp), CarePlan.status(?cp, carePlan_active), CarePlan.dailyPerMealGlucoseLevel.goal(?cp, ?premealGoal), goal(?premealGoal), Goal.target(?premealGoal, ?premealGoalComp), goalTargetComponent(?premealGoalComp), Goal.target.detailRange(?premealGoalComp, ?premealGoalComp_range), range(?premealGoalComp_range), Range.low(?premealGoalComp_range, ?premealGoalComp_range_low), quantity(?premealGoalComp_range_low), Quantity.value(?premealGoalComp_range_low, ?premealGoalComp_range_low_value), Quantity.code(?premealGoalComp_range_low, milligram_per_deciliter), hasValue(?premealGoalComp_range_low_value, ?low), Range.high(?premealGoalComp_range, ?premealGoalComp_range_high), quantity(?premealGoalComp_range_high), Quantity.value(?premealGoalComp_range_high, ?premealGoalComp_range_high_value), Quantity.code(?premealGoalComp_range_high, milligram_per_deciliter), hasValue(?premealGoalComp_range_high_value, ?high), swrlb:add(?pre_pbg, ?low, ?high), swrlb:divide(?pbg, ?pre_pbg, 2), swrlb:subtract(?DBG, ?cbg, ?pbg), swrlb:divide(?n1, ?DBG, ?isf_v), swrlb:divide(?n2, ?mc, ?icr_v), swrlb:add(?md, ?n1, ?n2), swrlb:divide(?pre_final, ?bc, 4), swrlb:divide(?final, ?pre_final, ?icr_v), swrlb:subtract(?md_final, ?md, ?final), CarePlan.activity(?cp, ?cp_activity), carePlanActivityComponent(?cp_activity), CarePlan.activity.reference(?cp_activity, ?nutr), nutritionOrder(?nutr), NutritionOrder.meal(?nutr, ?meal), breakfast(?meal) -> Meal.insulinUnitsForCarbs(?meal, ?n2), Meal.correctionInsulinUnits(?meal, ?n1), meal.totalBolusInsulinDosage(?meal, ?md_final), Meal.insulinUnitsForExercise(?meal, ?final)
34. diseaseContradictWithDrug(?dis, ?m), hasPatientProfile(?p, ?prof), hasComplication(?prof, ?cond), patientProfile(?prof), disease(?dis), medication(?m), condition(?cond), patient(?p), Condition.disease(?cond, ?dis), lispro(?m) -> patientContradictWithLispro(?p)
35. patient(?p), patientProfile(?prof), hasPatientProfile(?p, ?prof), hasInsulinSensitivityFactor(?prof, ?isf), quantity(?isf), Quantity.value(?isf, ?isf_dec), hasValue(?isf_dec, ?isf_v), hasInsulinToCarbohydrateRatio(?prof, ?icr), quantity(?icr), Quantity.value(?icr, ?icr_dec), hasValue(?icr_dec, ?icr_v), hasObservationValue(?prof, ?breakfastBGObs), lunchGlucoseObservationValue(?breakfastBGObs), isCurrent(?breakfastBGObs, true), Observation.valueQuantity(?breakfastBGObs, ?breakfastBGObs_value), quantity(?breakfastBGObs_value), Quantity.value(?breakfastBGObs_value, ?breakfastBGObs_value_dec), Quantity.code(?breakfastBGObs_value, milligram_per_deciliter), hasValue(?breakfastBGObs_value_dec, ?cbg), hasObservationValue(?prof, ?weight), weightValue(?weight), Observation.valueQuantity(?weight, ?weight_quant), quantity(?weight_quant), Quantity.code(?weight_quant, kilogram), Quantity.value(?weight_quant, ?weight_quant_value), hasValue(?weight_quant_value, ?w), swrlb:multiply(?pre_bc, ?met, ?w), swrlb:multiply(?bc, ?pre_bc, ?hours), hasObservationValue(?prof, ?neededCarbs), neededToEatCarbs(?neededCarbs), isCurrent(?neededCarbs, true), Observation.valueQuantity(?neededCarbs, ?neededCarbs_value), quantity(?neededCarbs_value), Quantity.value(?neededCarbs_value, ?neededCarbs_value_dec), Quantity.code(?neededCarbs_value, gram), hasValue(?neededCarbs_value_dec, ?mc), hasObservationValue(?prof, ?exercise), exerciseByMET(?exercise), isCurrent(?exercise, true), Observation.component(?exercise, ?exercise_comp), ObservationComponentComponent(?exercise_comp), Observation.component.code(?exercise_comp, ?exercise_comp_codeable), CodeableConcept.coding(?exercise_comp_codeable, ?exercise_comp_codeable_coding), coding(?exercise_comp_codeable_coding), Coding.code(?exercise_comp_codeable_coding, ?exercise_comp_codeable_coding_code), Coding.system(?exercise_comp_codeable_coding, SNOMED_CT), hasValue(?exercise_comp_codeable_coding_code, "698834005"^^xsd:string), Observation.component.valueQuantity(?exercise_comp, ?exercise_comp_quant), quantity(?exercise_comp_quant), Quantity.value(?exercise_comp_quant, ?exercise_comp_quant_value), hasValue(?exercise_comp_quant_value, ?met), Observation.component(?exercise, ?exercise_comp_dur), ObservationComponentComponent(?exercise_comp_dur), Observation.component.code(?exercise_comp_dur, ?exercise_comp_dur_codeable), CodeableConcept.coding(?exercise_comp_dur_codeable, ?eexercise_comp_dur_codeable_coding), coding(?exercise_comp_dur_codeable_coding), Coding.code(?exercise_comp_dur_codeable_coding, ?exercise_comp_dur_codeable_coding_code), Coding.system(?exercise_comp_dur_codeable_coding, SNOMED_CT), hasValue(?exercise_comp_dur_codeable_coding_code, "103335007"^^xsd:string), Observation.component.valueQuantity(?exercise_comp_dur, ?exercise_comp_dur_quant), quantity(?exercise_comp_dur_quant), Quantity.value(?exercise_comp_dur_quant, ?exercise_comp_dur_quant_value), hasValue(?exercise_comp_dur_quant_value, ?hours), hasCarePlan(?prof, ?cp), carePlan(?cp), CarePlan.status(?cp, carePlan_active), CarePlan.dailyPerMealGlucoseLevel.goal(?cp, ?premealGoal), goal(?premealGoal), Goal.target(?premealGoal, ?premealGoalComp), goalTargetComponent(?premealGoalComp), Goal.target.detailRange(?premealGoalComp, ?premealGoalComp_range), range(?premealGoalComp_range), Range.low(?premealGoalComp_range, ?premealGoalComp_range_low), quantity(?premealGoalComp_range_low), Quantity.value(?premealGoalComp_range_low, ?premealGoalComp_range_low_value), Quantity.code(?premealGoalComp_range_low, milligram_per_deciliter), hasValue(?premealGoalComp_range_low_value, ?low), Range.high(?premealGoalComp_range, ?premealGoalComp_range_high), quantity(?premealGoalComp_range_high), Quantity.value(?premealGoalComp_range_high, ?premealGoalComp_range_high_value), Quantity.code(?premealGoalComp_range_high, milligram_per_deciliter), hasValue(?premealGoalComp_range_high_value, ?high), swrlb:add(?pre_pbg, ?low, ?high), swrlb:divide(?pbg, ?pre_pbg, 2), swrlb:subtract(?DBG, ?cbg, ?pbg), swrlb:divide(?n1, ?DBG, ?isf_v), swrlb:divide(?n2, ?mc, ?icr_v), swrlb:add(?md, ?n1, ?n2), swrlb:divide(?pre_final, ?bc, 4), swrlb:divide(?final, ?pre_final, ?icr_v), swrlb:subtract(?md_final, ?md, ?final), CarePlan.activity(?cp, ?cp_activity), carePlanActivityComponent(?cp_activity), CarePlan.activity.reference(?cp_activity, ?nutr), nutritionOrder(?nutr), NutritionOrder.meal(?nutr, ?meal), lunch(?meal) -> Meal.insulinUnitsForCarbs(?meal, ?n2), Meal.correctionInsulinUnits(?meal, ?n1), meal.totalBolusInsulinDosage(?meal, ?md_final), Meal.insulinUnitsForExercise(?meal, ?final)
36. patient(?p), patientProfile(?prof), hasPatientProfile(?p, ?prof), hasObservationValue(?prof, ?bmi), BMI(?bmi), Observation.valueQuantity(?bmi, ?quant2), quantity(?quant2), Quantity.value(?quant2, ?val2), decimal(?val2), hasValue(?val2, ?bmi_value), swrlb:greaterThanOrEqual(?bmi_value, 18.5), swrlb:lessThan(?bmi_value, 25.0) -> hasComplication(?prof, normalWeight_condition)
37. patient(?p), condition(?cond), patientProfile(?prof), hasComplication(?prof, ?cond), hasPatientProfile(?p, ?prof), obesity(?obesity), Condition.disease(?cond, ?obesity) -> isInsulinResistant(?p, true)
38. patientHasNoLongActingContradict(?p), patientProfile(?prof), hasPatientProfile(?p, ?prof), hasPreferedInsulinRegimen(?prof, "DP"^^xsd:string), carePlan(?cp), hasCarePlan(?prof, ?cp), FixedRegimen(?ir), hasInsulinRegimen(?cp, ?ir) -> hasFixedTwoshotsEveningIntermActingInsulin(?ir, detemir), hasFixedTwoshotsMorningIntermActingInsulin(?ir, detemir)
39. patient(?p), patientProfile(?prof), hasPatientProfile(?p, ?prof), hasObservationValue(?prof, ?bmi), BMI(?bmi), Observation.valueQuantity(?bmi, ?quant2), quantity(?quant2), Quantity.value(?quant2, ?val2), decimal(?val2), hasValue(?val2, ?bmi_value), swrlb:greaterThanOrEqual(?bmi_value, 30.0) -> hasComplication(?prof, obesity_condition)
40. patient(?p), patientProfile(?prof), hasPatientProfile(?p, ?prof), hasInsulinSensitivityFactor(?prof, ?isf), quantity(?isf), Quantity.value(?isf, ?isf_dec), hasValue(?isf_dec, ?isf_v), hasInsulinToCarbohydrateRatio(?prof, ?icr), quantity(?icr), Quantity.value(?icr, ?icr_dec), hasValue(?icr_dec, ?icr_v), hasObservationValue(?prof, ?breakfastBGObs), lunchGlucoseObservationValue(?breakfastBGObs), isCurrent(?breakfastBGObs, true), Observation.valueQuantity(?breakfastBGObs, ?breakfastBGObs_value), quantity(?breakfastBGObs_value), Quantity.value(?breakfastBGObs_value, ?breakfastBGObs_value_dec), Quantity.code(?breakfastBGObs_value, milligram_per_deciliter), hasValue(?breakfastBGObs_value_dec, ?cbg), hasObservationValue(?prof, ?neededCarbs), neededToEatCarbs(?neededCarbs), isCurrent(?neededCarbs, true), Observation.valueQuantity(?neededCarbs, ?neededCarbs_value), quantity(?neededCarbs_value), Quantity.value(?neededCarbs_value, ?neededCarbs_value_dec), Quantity.code(?neededCarbs_value, gram), hasValue(?neededCarbs_value_dec, ?mc), hasCarePlan(?prof, ?cp), carePlan(?cp), CarePlan.status(?cp, carePlan_active), CarePlan.dailyPerMealGlucoseLevel.goal(?cp, ?premealGoal), goal(?premealGoal), Goal.target(?premealGoal, ?premealGoalComp), goalTargetComponent(?premealGoalComp), Goal.target.detailRange(?premealGoalComp, ?premealGoalComp_range), range(?premealGoalComp_range), Range.low(?premealGoalComp_range, ?premealGoalComp_range_low), quantity(?premealGoalComp_range_low), Quantity.value(?premealGoalComp_range_low, ?premealGoalComp_range_low_value), Quantity.code(?premealGoalComp_range_low, milligram_per_deciliter), hasValue(?premealGoalComp_range_low_value, ?low), Range.high(?premealGoalComp_range, ?premealGoalComp_range_high), quantity(?premealGoalComp_range_high), Quantity.value(?premealGoalComp_range_high, ?premealGoalComp_range_high_value), Quantity.code(?premealGoalComp_range_high, milligram_per_deciliter), hasValue(?premealGoalComp_range_high_value, ?high), swrlb:add(?pre_pbg, ?low, ?high), swrlb:divide(?pbg, ?pre_pbg, 2), swrlb:subtract(?DBG, ?cbg, ?pbg), swrlb:divide(?n1, ?DBG, ?isf_v), swrlb:divide(?n2, ?mc, ?icr_v), swrlb:add(?md, ?n1, ?n2), CarePlan.activity(?cp, ?cp_activity), carePlanActivityComponent(?cp_activity), CarePlan.activity.reference(?cp_activity, ?nutr), nutritionOrder(?nutr), NutritionOrder.meal(?nutr, ?meal), lunch(?meal) -> Meal.insulinUnitsForCarbs(?meal, ?n2), Meal.correctionInsulinUnits(?meal, ?n1), meal.totalBolusInsulinDosage(?meal, ?md)
41. code(?cd), codeableConcept(?cc), coding(?code), string(?disp), string(?st), uri(?u), patient(?p), condition(?cond), patientProfile(?prof), CodeableConcept.coding(?cc, ?code), CodeableConcept.text(?cc, ?st), Coding.code(?code, ?cd), Coding.display(?code, ?disp), Coding.system(?code, ?uri), hasComplication(?prof, ?cond), hasPatientProfile(?p, ?prof), hasValue(?cd, "414915002"^^xsd:string), hasValue(?disp, "obese"), hasValue(?st, "obese"^^xsd:string), hasValue(?u, "http://snomed.info/sct"^^xsd:string), Condition.code(?cond, ?cc), obesity(?obesity), Condition.disease(?cond, ?obesity) -> isInsulinResistant(?p, true)
42. patient(?p), (hasHistoryOfHypoglycemia min 1 xsd:integer)(?p), hasEducationRecord(?p, ?er), emergencyLearningTopic(?em), insulinLearningTopic(?i) -> hasLearningTopic(?er, ?em), hasLearningTopic(?er, ?i)
43. patientContradictWithAspart(?p), patientProfile(?prof), hasPatientProfile(?p, ?prof), hasPreferedInsulinRegimen(?prof, "IIT"^^xsd:string), carePlan(?cp), hasCarePlan(?prof, ?cp), intensiveInsulinTherapy(?ir), hasInsulinRegimen(?cp, ?ir) -> hasBolusInsulin(?ir, glulisine)
44. patient(?p), patientProfile(?prof), hasPatientProfile(?p, ?prof), hasComplication(?prof, ?cond), condition(?cond), Condition.disease(?cond, ?d), 'diabetic ketoacidosis'(?d) -> forbiddenFromExercise(?p)
45. swrlb:subtract(?pre_final, ?liw, ?value1), swrlb:add(?final, ?pre_final, ?value1), hasCarePlan(?prof, ?cp), isCurrent(?w, true), Quantity.value(?w_quant, ?w_val), coding(?desc_coding), hasPatientProfile(?p, ?prof), quantity(?wgt_quant), Quantity.code(?w_quant, kilogram), codeableConcept(?desc), Goal.target.detailQuantity(?wgt, ?wgt_quant), quantity(?w_quant), CodeableConcept.coding(?desc, ?desc_coding), swrlb:add(?total_cal, ?factor, ?curr_cal), swrlb:divide(?factor, ?pre_factor, ?day), swrlb:lessThan(?value1, ?liw), Quantity.value(?wgt_quant, ?wgt_quant_value), goalTargetComponent(?wgt), weightValue(?w), decimal(?w_val), Coding.display(?desc_coding, ?desc_coding_display), Goal.target(?wg, ?wgt), hasLowestIdealWeightInKG(?prof, ?liw), Quantity.value(?nu_order_quant, ?nu_order_quant_value), hasCaloriesForCurrentWeight(?prof, ?curr_cal), swrlb:multiply(?pre_factor, ?pre_final, 7700), quantity(?nu_order_quant), hasValue(?w_val, ?value1), CarePlan.activity.reference(?act, ?nu_order), patientProfile(?prof), daysToLoseOrGainWeight(?prof, ?day), patient(?p), carePlan(?cp), Goal.subject(?wg, ?p), NutritionOrder.dailyCalories(?nu_order, ?nu_order_quant), carePlanActivityComponent(?act), Observation.valueQuantity(?w, ?w_quant), Goal.description(?wg, ?desc), hasObservationValue(?prof, ?w), CarePlan.activity(?cp, ?act), CarePlan.weight.goal(?cp, ?wg), nutritionOrder(?nu_order), Coding.code(?desc_coding, ?desc_coding_code), decimal(?nu_order_quant_value) -> hasValue(?desc_coding_display, "weight gain regimen"^^xsd:string), hasValue(?desc_coding_code, "388978005"^^xsd:string), hasValue(?wgt_quant_value, ?final), Quantity.system(?wgt_quant, UCUM_coding_system), Quantity.code(?nu_order_quant, kilocalorie_per_day), Quantity.code(?wgt_quant, kilogram), Quantity.system(?nu_order_quant, UCUM_coding_system), hasValue(?nu_order_quant_value, ?total_cal)
46. patient(?p), patientProfile(?prof), hasPatientProfile(?p, ?prof), hasCarePlan(?prof, ?cp), carePlan(?cp), hasInsulinRegimen(?cp, ?ir), intensiveInsulinTherapy(?ir), hasEducationRecord(?p, ?er), monitoringLearningTopic(?ml), insulinLearningTopic(?i), dietLearningTopic(?dit) -> hasLearningTopic(?er, ?ml), hasLearningTopic(?er, ?i), hasLearningTopic(?er, ?dit)
47. adult(?p), Person.gender(?p, ?gen), genderCode(?gen), hasValue(?gen, "female"^^xsd:string), isPragnant(?p, true), patientProfile(?prof), hasPatientProfile(?p, ?prof), carePlan(?cp), hasCarePlan(?prof, ?cp), intensiveInsulinTherapy(?iit) -> hasInsulinRegimen(?cp, ?iit)
48. hasCarePlan(?prof, ?cp), isCurrent(?w, true), Quantity.value(?w_quant, ?w_val), hasHighestIdealWeightInKG(?prof, ?hiw), coding(?desc_coding), hasPatientProfile(?p, ?prof), decimal(?wgt_quant_value), swrlb:greaterThanOrEqual(?value1, ?liw), quantity(?wgt_quant), Quantity.code(?w_quant, kilogram), codeableConcept(?desc), Goal.target.detailQuantity(?wgt, ?wgt_quant), quantity(?w_quant), CodeableConcept.coding(?desc, ?desc_coding), Quantity.value(?wgt_quant, ?wgt_quant_value), goalTargetComponent(?wgt), weightValue(?w), decimal(?w_val), Coding.display(?desc_coding, ?desc_coding_display), Goal.target(?wg, ?wgt), hasLowestIdealWeightInKG(?prof, ?liw), Quantity.value(?nu_order_quant, ?nu_order_quant_value), hasCaloriesForCurrentWeight(?prof, ?curr_cal), quantity(?nu_order_quant), hasValue(?w_val, ?value1), CarePlan.activity.reference(?act, ?nu_order), patientProfile(?prof), swrlb:lessThanOrEqual(?value1, ?hiw), patient(?p), carePlan(?cp), Goal.subject(?wg, ?p), NutritionOrder.dailyCalories(?nu_order, ?nu_order_quant), carePlanActivityComponent(?act), Observation.valueQuantity(?w, ?w_quant), Goal.description(?wg, ?desc), hasObservationValue(?prof, ?w), CarePlan.activity(?cp, ?act), CarePlan.weight.goal(?cp, ?wg), nutritionOrder(?nu_order), Coding.code(?desc_coding, ?desc_coding_code), decimal(?nu_order_quant_value) -> hasValue(?nu_order_quant_value, ?curr_cal), Quantity.system(?wgt_quant, UCUM_coding_system), Quantity.code(?nu_order_quant, kilocalorie_per_day), hasValue(?wgt_quant_value, ?value1), Quantity.code(?wgt_quant, kilogram), hasValue(?desc_coding_display, "behavior to maintain weight"^^xsd:string), Quantity.system(?nu_order_quant, UCUM_coding_system), hasValue(?desc_coding_code, "439415004"^^xsd:string)
49. code(?cd), codeableConcept(?cc), coding(?code), string(?disp), string(?st), uri(?u), patient(?p), condition(?cond), patientProfile(?prof), CodeableConcept.coding(?cc, ?code), CodeableConcept.text(?cc, ?st), Coding.code(?code, ?cd), Coding.display(?code, ?disp), Coding.system(?code, ?uri), hasComplication(?prof, ?cond), hasPatientProfile(?p, ?prof), hasValue(?cd, "197321007"^^xsd:string), hasValue(?disp, "steatosis of liver"), hasValue(?st, "steatosis of liver"^^xsd:string), hasValue(?u, "http://snomed.info/sct"^^xsd:string), Condition.code(?cond, ?cc), Condition.disease(?cond, ?steatosis_of_liver), 'steatosis of liver'(?steatosis_of_liver) -> isInsulinResistant(?p, true)
50. patient(?p), Person.gender(?p, gender_female), condition(?cond), patientProfile(?prof), hasComplication(?prof, ?cond), hasPatientProfile(?p, ?prof), 'chronic bronchitis'(?cb), Condition.disease(?cond, ?cb) -> forbiddenFromExercise(?p)
51. patient(?p), patientProfile(?prof), hasPatientProfile(?p, ?prof), hasComplication(?prof, ?c), condition(?c), Condition.disease(?c, ?dis), hasEducationRecord(?p, ?er), educationRecord(?er), complicationLearningTopic(?cm), insulinLearningTopic(?i) -> hasLearningTopic(?er, ?cm), hasLearningTopic(?er, ?i)
52. CodeableConcept.coding(?dinner_dosage_timing_codeable, ?dinner_dosage_timing_codeable_coding), hasCarePlan(?prof, ?cp), hasPreferedInsulinRegimen(?prof, "IIT"^^xsd:string), hasInsulinRegimen(?cp, ?ir), swrlb:divide(?pre_final, ?f, 3), hasPatientProfile(?p, ?prof), dosage(?dinner_dosage), Coding.display(?dinner_dosage_timing_codeable_coding, ?dinner_dosage_timing_codeable_coding_display), Dosage.doseSimpleQuantity(?dinner_dosage, ?dinner_dosage_quant), intensiveInsulinTherapy(?ir), Quantity.value(?dinner_dosage_quant, ?dinner_dosage_quant_decimal), codeableConcept(?dinner_dosage_route), Dosage.route(?dinner_dosage, ?dinner_dosage_route), codeableConcept(?dinner_dosage_timing_codeable), Coding.code(?dinner_dosage_timing_codeable_coding, ?dinner_dosage_timing_codeable_coding_code), quantity(?dinner_dosage_quant), coding(?dinner_dosage_route_coding), hasBolusInsulinUnits(?ir, ?f), Timing.code(?dinner_dosage_timing, ?dinner_dosage_timing_codeable), swrlb:ceiling(?final, ?pre_final), patientProfile(?prof), Coding.code(?dinner_dosage_route_coding, ?dinner_dosage_route_coding_code), patient(?p), carePlan(?cp), Coding.display(?dinner_dosage_route_coding, ?dinner_dosage_route_coding_display), timing(?dinner_dosage_timing), CodeableConcept.coding(?dinner_dosage_route, ?dinner_dosage_route_coding), Dosage.timing(?dinner_dosage, ?dinner_dosage_timing), hasBolusDinnerDose(?ir, ?dinner_dosage) -> hasValue(?dinner_dosage_timing_codeable_coding_display, "daily with dinner"^^xsd:string), hasValue(?dinner_dosage_quant_decimal, ?final), Quantity.code(?dinner_dosage_quant, units), Coding.system(?dinner_dosage_route_coding, SNOMED_CT), Coding.system(?dinner_dosage_timing_codeable_coding, SNOMED_CT), Quantity.system(?dinner_dosage_quant, UCUM_coding_system), hasValue(?dinner_dosage_route_coding_display, "injection"^^xsd:string), hasValue(?dinner_dosage_route_coding_code, "59108006"^^xsd:string), hasValue(?dinner_dosage_timing_codeable_coding_code, "1771000175105"^^xsd:string)
53. diseaseContradictWithDrug(?dis, ?m), hasPatientProfile(?p, ?prof), hasComplication(?prof, ?cond), patientProfile(?prof), disease(?dis), medication(?m), condition(?cond), patient(?p), Condition.disease(?cond, ?dis), glulisine(?m) -> patientContradictWithGlulisine(?p)
54. child(?p), patientProfile(?prof), carePlan(?cp), goal(?a1c_g), hasCarePlan(?prof, ?cp), CarePlan.HbA1C.goal(?cp, ?a1c_g), Goal.target(?a1c_g, ?a1c_tar), goalTargetComponent(?a1c_tar), Goal.target.detailQuantity(?a1c_tar, ?a1c_quant), quantity(?a1c_quant), Quantity.value(?a1c_quant, ?decim), decimal(?decim) -> Quantity.code(?a1c_quant, percent), hasValue(?decim, 7.5), Quantity.comparator(?a1c_quant, comparator_code_lessThan)
55. patient(?p), patientProfile(?prof), hasPatientProfile(?p, ?prof), hasActivityLevel(?prof, ?al), carePlan(?cp), isCurrent(?cp, true), hasCarePlan(?prof, ?cp), CarePlan.activity(?cp, ?act), carePlanActivityComponent(?act), CarePlan.activity.reference(?act, ?nu_order), nutritionOrder(?nu_order), NutritionOrder.supplement(?nu_order, ?supp), nutritionOrderSupplementComponent(?supp), NutritionOrder.supplement.quantity(?supp, ?supp_quant), quantity(?supp_quant), Quantity.code(?supp_quant, ?c), hasValue(?c, "165109007"^^xsd:string), Quantity.value(?supp_quant, ?supp_quant_val), decimal(?supp_quant_val), hasValue(?supp_quant_val, ?bmr), swrlb:multiply(?pre_mc_v, ?bmr, ?al), swrlb:ceiling(?mc_v, ?pre_mc_v) -> hasCaloriesForCurrentWeight(?prof, ?mc_v)
56. patient(?p), patientProfile(?prof), hasPatientProfile(?p, ?prof), medicationStatement(?ms), hasPatientMedication(?prof, ?ms), medication(?m), MedicationStatement.medicationReference(?ms, ?m), drugContradictWithDrug(?m, ?m2), aspart(?m2) -> patientContradictWithAspart(?p)
57. patient(?p), Person.gender(?p, gender_female), condition(?cond), patientProfile(?prof), hasComplication(?prof, ?cond), hasPatientProfile(?p, ?prof), fetal_anemia(?fa), Condition.disease(?cond, ?fa) -> forbiddenFromExercise(?p)
58. child(?p), patientProfile(?prof), hasPatientProfile(?p, ?prof), carePlan(?cp), hasCarePlan(?prof, ?cp), CarePlan.status(?cp, carePlan_active), hasInsulinRegimen(?cp, ?ir), insulinRegimen(?ir), hasTotalDailyDose(?ir, ?tdd), swrlb:greaterThanOrEqual(?tdd, 10), hasInsulinToCarbohydrateRatio(?prof, ?icr), quantity(?icr), swrlb:divide(?result, 500, ?tdd), Quantity.value(?icr, ?icr_v), decimal(?icr_v) -> Quantity.code(?icr, carbs_per_unit), Quantity.comparator(?icr, comparator_code_equal), Quantity.value(?icr_v, ?result)
59. patient(?p), patientProfile(?prof), hasPatientProfile(?p, ?prof), hasInsulinSensitivityFactor(?prof, ?isf), quantity(?isf), Quantity.value(?isf, ?isf_dec), hasValue(?isf_dec, ?isf_v), hasInsulinToCarbohydrateRatio(?prof, ?icr), quantity(?icr), Quantity.value(?icr, ?icr_dec), hasValue(?icr_dec, ?icr_v), hasObservationValue(?prof, ?breakfastBGObs), dinnerGlucoseObservationValue(?breakfastBGObs), isCurrent(?breakfastBGObs, true), Observation.valueQuantity(?breakfastBGObs, ?breakfastBGObs_value), quantity(?breakfastBGObs_value), Quantity.value(?breakfastBGObs_value, ?breakfastBGObs_value_dec), Quantity.code(?breakfastBGObs_value, milligram_per_deciliter), hasValue(?breakfastBGObs_value_dec, ?cbg), hasObservationValue(?prof, ?weight), weightValue(?weight), Observation.valueQuantity(?weight, ?weight_quant), quantity(?weight_quant), Quantity.code(?weight_quant, kilogram), Quantity.value(?weight_quant, ?weight_quant_value), hasValue(?weight_quant_value, ?w), swrlb:multiply(?pre_bc, ?met, ?w), swrlb:multiply(?bc, ?pre_bc, ?hours), hasObservationValue(?prof, ?neededCarbs), neededToEatCarbs(?neededCarbs), isCurrent(?neededCarbs, true), Observation.valueQuantity(?neededCarbs, ?neededCarbs_value), quantity(?neededCarbs_value), Quantity.value(?neededCarbs_value, ?neededCarbs_value_dec), Quantity.code(?neededCarbs_value, gram), hasValue(?neededCarbs_value_dec, ?mc), hasObservationValue(?prof, ?exercise), exerciseByMET(?exercise), isCurrent(?exercise, true), Observation.component(?exercise, ?exercise_comp), ObservationComponentComponent(?exercise_comp), Observation.component.code(?exercise_comp, ?exercise_comp_codeable), CodeableConcept.coding(?exercise_comp_codeable, ?exercise_comp_codeable_coding), coding(?exercise_comp_codeable_coding), Coding.code(?exercise_comp_codeable_coding, ?exercise_comp_codeable_coding_code), Coding.system(?exercise_comp_codeable_coding, SNOMED_CT), hasValue(?exercise_comp_codeable_coding_code, "698834005"^^xsd:string), Observation.component.valueQuantity(?exercise_comp, ?exercise_comp_quant), quantity(?exercise_comp_quant), Quantity.value(?exercise_comp_quant, ?exercise_comp_quant_value), hasValue(?exercise_comp_quant_value, ?met), Observation.component(?exercise, ?exercise_comp_dur), ObservationComponentComponent(?exercise_comp_dur), Observation.component.code(?exercise_comp_dur, ?exercise_comp_dur_codeable), CodeableConcept.coding(?exercise_comp_dur_codeable, ?eexercise_comp_dur_codeable_coding), coding(?exercise_comp_dur_codeable_coding), Coding.code(?exercise_comp_dur_codeable_coding, ?exercise_comp_dur_codeable_coding_code), Coding.system(?exercise_comp_dur_codeable_coding, SNOMED_CT), hasValue(?exercise_comp_dur_codeable_coding_code, "103335007"^^xsd:string), Observation.component.valueQuantity(?exercise_comp_dur, ?exercise_comp_dur_quant), quantity(?exercise_comp_dur_quant), Quantity.value(?exercise_comp_dur_quant, ?exercise_comp_dur_quant_value), hasValue(?exercise_comp_dur_quant_value, ?hours), hasCarePlan(?prof, ?cp), carePlan(?cp), CarePlan.status(?cp, carePlan_active), CarePlan.dailyPerMealGlucoseLevel.goal(?cp, ?premealGoal), goal(?premealGoal), Goal.target(?premealGoal, ?premealGoalComp), goalTargetComponent(?premealGoalComp), Goal.target.detailRange(?premealGoalComp, ?premealGoalComp_range), range(?premealGoalComp_range), Range.low(?premealGoalComp_range, ?premealGoalComp_range_low), quantity(?premealGoalComp_range_low), Quantity.value(?premealGoalComp_range_low, ?premealGoalComp_range_low_value), Quantity.code(?premealGoalComp_range_low, milligram_per_deciliter), hasValue(?premealGoalComp_range_low_value, ?low), Range.high(?premealGoalComp_range, ?premealGoalComp_range_high), quantity(?premealGoalComp_range_high), Quantity.value(?premealGoalComp_range_high, ?premealGoalComp_range_high_value), Quantity.code(?premealGoalComp_range_high, milligram_per_deciliter), hasValue(?premealGoalComp_range_high_value, ?high), swrlb:add(?pre_pbg, ?low, ?high), swrlb:divide(?pbg, ?pre_pbg, 2), swrlb:subtract(?DBG, ?cbg, ?pbg), swrlb:divide(?n1, ?DBG, ?isf_v), swrlb:divide(?n2, ?mc, ?icr_v), swrlb:add(?md, ?n1, ?n2), swrlb:divide(?pre_final, ?bc, 4), swrlb:divide(?final, ?pre_final, ?icr_v), swrlb:subtract(?md_final, ?md, ?final), CarePlan.activity(?cp, ?cp_activity), carePlanActivityComponent(?cp_activity), CarePlan.activity.reference(?cp_activity, ?nutr), nutritionOrder(?nutr), NutritionOrder.meal(?nutr, ?meal), dinner(?meal) -> Meal.insulinUnitsForCarbs(?meal, ?n2), Meal.correctionInsulinUnits(?meal, ?n1), meal.totalBolusInsulinDosage(?meal, ?md_final), Meal.insulinUnitsForExercise(?meal, ?final)
60. patient(?p), hasEducationRecord(?p, ?er), educationRecord(?er), hasLearningStyle(?er, ?v), reading(?red), hasLearningTopic(?er, ?top), insulinLearningTopic(?top), hasLearningCourse(?er, ?course), learningCourse(?course), coursePeriod(?course, ?period), learningCourseCode(?course, ?ccode), patientProfile(?prof), hasPatientProfile(?p, ?prof), hasCarePlan(?prof, ?cp), carePlan(?cp), CarePlan.activity(?cp, ?cp_a), carePlanActivityComponent(?cp_a), CarePlan.activity.reference(?cp_a, ?cp_a_p), procedureRequest(?cp_a_p), ProcedureRequest.category(?cp_a_p, ?cat), codeableConcept(?cat), CodeableConcept.coding(?cat, ?cat_coding), coding(?cat_coding), Coding.code(?cat_coding, ?cat_coding_code), Coding.display(?cat_coding, ?cat_coding_display) -> ProcedureRequest.basedOn(?cp_a_p, ?cp), ProcedureRequest.code(?cp_a_p, ?ccode), ProcedureRequest.subject(?cp_a_p, ?p), Coding.system(?cat_coding, SNOMED_CT), hasValue(?cat_coding_code, "311401005"^^xsd:string), hasValue(?cat_coding_display, "Patient education (procedure)"^^xsd:string), ProcedureRequest.occurrencePeriod(?cp_a_p, ?period)
61. adult(?p), educationalAchievement(?p, "noEducation"^^xsd:string), hasEducationRecord(?p, ?er), visual(?v), auditory(?a) -> hasLearningStyle(?er, ?v), hasLearningStyle(?er, ?a)
62. patient(?p), patientProfile(?prof), hasPatientProfile(?p, ?prof), hasPreferedInsulinRegimen(?prof, "DP"^^xsd:string), carePlan(?cp), hasCarePlan(?prof, ?cp), FixedRegimen(?ir), hasInsulinRegimen(?cp, ?ir), hasTotalDailyDose(?ir, ?tdd), dosage(?m_l), hasFixedTwoshotsMorningIntermActingInsulinDose(?ir, ?m_l), Dosage.doseSimpleQuantity(?m_l, ?m_l_quant), quantity(?m_l_quant), Quantity.value(?m_l_quant, ?m_l_quant_decim), swrlb:divide(?m_factor_l, 4, 9), swrlb:multiply(?m_l_quant_decim_value, ?m_factor_l, ?tdd), swrlb:ceiling(?fin, ?m_l_quant_decim_value) -> hasValue(?m_l_quant_decim, ?fin)
63. patient(?p), patientProfile(?prof), hasPatientProfile(?p, ?prof), hasObservationValue(?prof, ?bmi), BMI(?bmi), Observation.valueQuantity(?bmi, ?quant2), quantity(?quant2), Quantity.value(?quant2, ?val2), decimal(?val2), hasValue(?val2, ?bmi_value), swrlb:greaterThanOrEqual(?bmi_value, 25.0), swrlb:lessThan(?bmi_value, 30.0) -> hasComplication(?prof, overWeight_condition)
64. patient(?p), patientProfile(?prof), hasPatientProfile(?p, ?prof), hasComplication(?prof, ?c), condition(?c), Condition.disease(?c, ?dis), disease(?dis), diseaseContradictWithFood(?dis, ?food), nutrient(?food), hasCarePlan(?prof, ?cp), carePlan(?cp), CarePlan.activity(?cp, ?act), carePlanActivityComponent(?act), CarePlan.activity.reference(?act, ?nu_order), nutritionOrder(?nu_order) -> hasForbiddenFood(?prof, ?food), NutritionOrder.excludeFoodModifier(?nu_order, ?food)
65. patient(?p), patientProfile(?prof), hasPatientProfile(?p, ?prof), hasComplication(?prof, ?c), condition(?c), Condition.disease(?c, ?dis), 'ischemic heart disease'(?dis) -> forbiddenFromExercise(?p)
66. patient(?p), hasPreferedBGUoM(?p, "mg/dl"^^xsd:string), patientProfile(?prof), hasPatientProfile(?p, ?prof), carePlan(?cp), hasCarePlan(?prof, ?cp), CarePlan.status(?cp, carePlan_active), hasInsulinRegimen(?cp, ?ir), insulinRegimen(?ir), hasTotalDailyDose(?ir, ?tdd), hasInsulinSensitivityFactor(?prof, ?isf), quantity(?isf), swrlb:divide(?result, 1800, ?tdd), Quantity.value(?isf, ?isf_v), decimal(?isf_v) -> Quantity.code(?isf, milligram_per_deciliter_per_unit), Quantity.comparator(?isf, comparator_code_equal), Quantity.value(?isf_v, ?result)
67. patient(?p), patientProfile(?prof), hasPatientProfile(?p, ?prof), hasLifeStyle(?prof, "lightly active"^^xsd:string) -> hasActivityLevel(?prof, "1.375"^^xsd:double)
68. patient(?p), patientProfile(?prof), hasPatientProfile(?p, ?prof), hasComplication(?prof, ?c), condition(?c), Condition.disease(?c, ?dis), acute_infectious_disease(?dis) -> forbiddenFromExercise(?p)
69. patientContradictWithGlargine(?p), patientProfile(?prof), hasPatientProfile(?p, ?prof), hasPreferedInsulinRegimen(?prof, "DP"^^xsd:string), carePlan(?cp), hasCarePlan(?prof, ?cp), FixedRegimen(?ir), hasInsulinRegimen(?cp, ?ir) -> hasFixedTwoshotsEveningIntermActingInsulin(?ir, detemir), hasFixedTwoshotsMorningIntermActingInsulin(?ir, detemir)
70. patient(?p), patientProfile(?prof), hasPatientProfile(?p, ?prof), hasObservationValue(?prof, ?bmi), isCurrent(?bmi, true), BMI(?bmi), Observation.valueQuantity(?bmi, ?quant2), quantity(?quant2), Quantity.code(?quant2, kilogram_per_square_meter), Quantity.value(?quant2, ?val2), decimal(?val2), hasValue(?val2, ?bmi_value), swrlb:lessThan(?bmi_value, 12.0) -> forbiddenFromExercise(?p)
71. Quantity.code(?quant3, meter), isCurrent(?w, true), swrlb:multiply(?h_v, ?value3, 5, 3), hasCarePlan(?prof, ?cp), swrlb:multiply(?a_v, ?value2, 6.755), nutritionOrderSupplementComponent(?supp), hasPatientProfile(?p, ?prof), Quantity.value(?quant1, ?val1), Quantity.value(?quant3, ?val3), swrlb:add(?pre_final, ?w_h_a, 66.5), NutritionOrder.supplement.type(?supp, ?supp_type), swrlb:subtract(?w_h_a, ?w_h, ?a_v), hasObservationValue(?prof, ?h), Person.gender(?p, gender_male), Coding.display(?supp_type_coding, ?supp_type_coding_display), swrlb:multiply(?w_v, ?value1, 13.75), isCurrent(?h, true), quantity(?supp_quant), NutritionOrder.supplement.quantity(?supp, ?supp_quant), hasValue(?val1, ?value1), hasValue(?val3, ?value3), Person.age(?p, ?ag), weightValue(?w), Quantity.value(?supp_quant, ?supp_quant_val), quantity(?quant1), Observation.valueQuantity(?w, ?quant1), quantity(?quant3), decimal(?supp_quant_val), CodeableConcept.coding(?supp_type, ?supp_type_coding), Coding.code(?supp_type_coding, ?supp_type_coding_code), swrlb:ceiling(?final, ?pre_final), CarePlan.activity.reference(?act, ?nu_order), codeableConcept(?supp_type), patientProfile(?prof), patient(?p), carePlanActivityComponent(?act), Observation.valueQuantity(?h, ?quant3), NutritionOrder.supplement(?nu_order, ?supp), hasObservationValue(?prof, ?w), CarePlan.activity(?cp, ?act), coding(?supp_type_coding), Quantity.code(?quant1, kilogram), height(?h), swrlb:add(?w_h, ?w_v, ?h_v), nutritionOrder(?nu_order), hasValue(?ag, ?value2), decimal(?val3), decimal(?val1) -> hasValue(?supp_quant_val, ?final), Quantity.system(?supp_quant, UCUM_coding_system), hasValue(?supp_type_coding_display, "basal metabolic rate (observable entity)"^^xsd:string), Coding.system(?supp_type_coding, SNOMED_CT), Quantity.code(?supp_quant, kilocalorie), hasValue(?supp_type_coding_code, "165109007"^^xsd:string)
72. patient(?p), Person.age(?p, ?ag), hasValue(?ag, ?value), swrlb:greaterThanOrEqual(?value, 55) -> oldAdult(?p)
73. patient(?p), Person.gender(?p, gender_female), condition(?cond), patientProfile(?prof), hasComplication(?prof, ?cond), hasPatientProfile(?p, ?prof), placenta_previa(?pp), Condition.disease(?cond, ?pp) -> forbiddenFromExercise(?p)
74. child(?p), patientProfile(?prof), hasPatientProfile(?p, ?prof), carePlan(?cp), hasCarePlan(?prof, ?cp), CarePlan.status(?cp, carePlan_active), hasInsulinRegimen(?cp, ?ir), insulinRegimen(?ir), hasTotalDailyDose(?ir, ?tdd), swrlb:lessThan(?tdd, 10), hasInsulinToCarbohydrateRatio(?prof, ?icr), quantity(?icr), swrlb:divide(?result, 300, ?tdd), Quantity.value(?icr, ?icr_v), decimal(?icr_v) -> Quantity.code(?icr, carbs_per_unit), Quantity.comparator(?icr, comparator_code_equal), Quantity.value(?icr_v, ?result)
75. patient(?p), hasEducationRecord(?p, ?er), educationRecord(?er), hasLearningStyle(?er, ?v), video(?red), hasLearningTopic(?er, ?top), insulinLearningTopic(?top) -> hasLearningCourse(?er, learningCourse_insulin_visual_insulinDosage), hasLearningCourse(?er, learningCourse_insulin_visual_insulinSensitivity), hasLearningCourse(?er, learningCourse_insulin_visual_insulinTherapy), hasLearningCourse(?er, learningCourse_insulin_visual_type_1_diabetes)
76. patientContradictWithDetemir(?p), patientProfile(?prof), hasPatientProfile(?p, ?prof), hasPreferedInsulinRegimen(?prof, "DP"^^xsd:string), carePlan(?cp), hasCarePlan(?prof, ?cp), FixedRegimen(?ir), hasInsulinRegimen(?cp, ?ir) -> hasFixedTwoshotsEveningIntermActingInsulin(?ir, lantus), hasFixedTwoshotsMorningIntermActingInsulin(?ir, lantus)
77. patient(?p), patientProfile(?prof), hasPatientProfile(?p, ?prof), hasGlucoseBehaviorBeforeBreakfast(?prof, "increasing"^^xsd:string), carePlan(?cp), isCurrent(?cp, true), CarePlan.status(?cp, carePlan_active), hasCarePlan(?prof, ?cp), hasInsulinRegimen(?cp, ?ir), intensiveInsulinTherapy(?ir), hasBasalInsulinMorningAdjustment(?ir, ?ba), basalInsulinAdjustment(?ba), hasQuantityInUnits(?ba, ?ba_q), quantity(?ba_q), Quantity.value(?ba_q, ?ba_q_v), decimal(?ba_q_v) -> isCurrent(?ba, true), basalInsulinAdjustType(?ba, "-"^^xsd:string), Quantity.code(?ba_q, percent), Quantity.comparator(?ba_q, comparator_code_equal), Quantity.system(?ba_q, UCUM_coding_system), hasValue(?ba_q_v, 10.0)
78. adolescent(?p), educationalAchievement(?p, "noEducation"^^xsd:string), hasEducationRecord(?p, ?er), educationRecord(?er), visual(?v), games(?g) -> hasLearningStyle(?er, ?v), hasLearningStyle(?er, ?g)
79. oldAdult(?p), patientProfile(?prof), hasPatientProfile(?p, ?prof), carePlan(?cp), hasCarePlan(?prof, ?cp), CarePlan.status(?cp, carePlan_active), hasInsulinRegimen(?cp, ?ir), insulinRegimen(?ir), hasTotalDailyDose(?ir, ?tdd), hasInsulinToCarbohydrateRatio(?prof, ?icr), quantity(?icr), swrlb:divide(?result, 500, ?tdd), Quantity.value(?icr, ?icr_v), decimal(?icr_v) -> Quantity.code(?icr, carbs_per_unit), Quantity.comparator(?icr, comparator_code_equal), Quantity.value(?icr_v, ?result)
80. adult(?p), isPragnant(?p, false), patientInHoneymoonPeriod(?p), hasHistoryOfHypoglycemia(?p, 0), patientProfile(?prof), carePlan(?cp), goal(?a1c_g), hasCarePlan(?prof, ?cp), CarePlan.HbA1C.goal(?cp, ?a1c_g), Goal.target(?a1c_g, ?a1c_tar), goalTargetComponent(?a1c_tar), Goal.target.detailQuantity(?a1c_tar, ?a1c_quant), quantity(?a1c_quant), Quantity.value(?a1c_quant, ?decim), decimal(?decim), goal(?preMeal_g), DifferentFrom (?a1c_g, ?preMeal_g), CarePlan.dailyPerMealGlucoseLevel.goal(?cp, ?preMeal_g), goalTargetComponent(?preMeal_tar), Goal.target.detailRange(?preMeal_tar, ?preMeal_range), range(?preMeal_range), Range.low(?preMeal_range, ?preMeal_quant_low), quantity(?preMeal_quant_low), Quantity.value(?preMeal_quant_low, ?decim_preMeal_low), decimal(?decim_preMeal_low), Range.high(?preMeal_range, ?preMeal_quant_high), quantity(?preMeal_quant_high), Quantity.value(?preMeal_quant_high, ?decim_preMeal_high), decimal(?decim_preMeal_high), Goal.target(?preMeal_g, ?preMeal_tar) -> Quantity.code(?a1c_quant, percent), hasValue(?decim, 6.0), Quantity.comparator(?a1c_quant, comparator_code_lessThan), Quantity.code(?preMeal_quant_low, milligram_per_deciliter), hasValue(?decim_preMeal_low, 80), Quantity.comparator(?preMeal_quant_low, comparator_code_greaterThanOrEqual), Quantity.code(?preMeal_quant_high, milligram_per_deciliter), hasValue(?decim_preMeal_high, 120), Quantity.comparator(?preMeal_quant_high, comparator_code_lessThanOrEqual)
81. patient(?p), condition(?cond), patientProfile(?prof), hasComplication(?prof, ?cond), hasPatientProfile(?p, ?prof), hypertension(?h), Condition.disease(?cond, ?h), condition(?cond2), hasComplication(?prof, ?cond2), dyslipidemia(?d), Condition.disease(?cond2, ?d), condition(?cond3), hasComplication(?prof, ?cond3), nephropathy(?n), Condition.disease(?cond3, ?n), condition(?cond4), hasComplication(?prof, ?cond4), 'Preproliferative retinopathy'(?pr), Condition.disease(?cond4, ?pr), Person.age(?p, ?age), decimal(?age), hasValue(?age, ?v), swrlb:greaterThan(?v, 30), hasObservationValue(?prof, ?obs), cigaretteSmoking(?obs), isCurrent(?obs, true), Observation.valueCodeableConcept(?obs, ?cc), codeableConcept(?cc), CodeableConcept.coding(?cc, smokingObservation) -> forbiddenFromExercise(?p)
82. patient(?p), patientProfile(?prof), hasPatientProfile(?p, ?prof), medicationStatement(?ms), hasPatientMedication(?prof, ?ms), medication(?m), MedicationStatement.medicationReference(?ms, ?m), drugContradictWithDrug(?m, ?m2), glulisine(?m2) -> patientContradictWithGlulisine(?p)
83. adult(?p), educationalAchievement(?p, "highEducation"^^xsd:string), hasEducationRecord(?p, ?er), reading(?r) -> hasLearningStyle(?er, ?r)
84. oldAdult(?p), notForbiddenFromExercise(?p), patientProfile(?prof), hasPatientProfile(?p, ?prof), hasRecommendedExercise(?prof, ?exe), 'flexibility activitie'(?exe), carePlan(?cp), CarePlan.status(?cp, carePlan_active), hasCarePlan(?prof, ?cp), CarePlan.activity(?cp, ?cp_a), carePlanActivityComponent(?cp_a), CarePlan.activity.reference(?cp_a, ?exe_p), exercisePlan(?exe_p), exercisePlan.haspart(?exe_p, ?exe_p_p), exercisePlanComponent(?exe_p_p), exercisePlan.component.activityFrequency(?exe_p_p, ?frequency), timing(?frequency), Timing.repeat.boundsRange(?frequency, ?frequency_range), range(?frequency_range), Range.low(?frequency_range, ?frequency_range_low_q), quantity(?frequency_range_low_q), Quantity.value(?frequency_range_low_q, ?frequency_range_low_q_value) -> exercisePlan.subject(?exe_p, ?p), exercisePlan.isPartOf(?exe_p, ?cp), exercisePlan.component.intensity(?exe_p_p, intensity_level_moderate), Quantity.code(?frequency_range_low_q, days_per_week), Quantity.comparator(?frequency_range_low_q, comparator_code_equal), hasValue(?frequency_range_low_q_value, 3), exercisePlan.component.exerciseType(?exe_p_p, ?exe)
85. patientContradictWithGlargine(?p), patientProfile(?prof), hasPatientProfile(?p, ?prof), hasPreferedInsulinRegimen(?prof, "IIT"^^xsd:string), carePlan(?cp), hasCarePlan(?prof, ?cp), intensiveInsulinTherapy(?ir), hasInsulinRegimen(?cp, ?ir) -> hasBasalInsulin(?ir, detemir)
86. adult(?p), patientProfile(?prof), hasPatientProfile(?p, ?prof), carePlan(?cp), hasCarePlan(?prof, ?cp), CarePlan.status(?cp, carePlan_active), hasInsulinRegimen(?cp, ?ir), insulinRegimen(?ir), hasTotalDailyDose(?ir, ?tdd), hasInsulinToCarbohydrateRatio(?prof, ?icr), quantity(?icr), swrlb:divide(?result, 500, ?tdd), Quantity.value(?icr, ?icr_v), decimal(?icr_v) -> Quantity.code(?icr, carbs_per_unit), Quantity.comparator(?icr, comparator_code_equal), Quantity.value(?icr_v, ?result)
87. patient(?p), patientProfile(?prof), hasPatientProfile(?p, ?prof), hasInsulinSensitivityFactor(?prof, ?isf), quantity(?isf), Quantity.value(?isf, ?isf_dec), hasValue(?isf_dec, ?isf_v), hasInsulinToCarbohydrateRatio(?prof, ?icr), quantity(?icr), Quantity.value(?icr, ?icr_dec), hasValue(?icr_dec, ?icr_v), hasObservationValue(?prof, ?breakfastBGObs), dinnerGlucoseObservationValue(?breakfastBGObs), isCurrent(?breakfastBGObs, true), Observation.valueQuantity(?breakfastBGObs, ?breakfastBGObs_value), quantity(?breakfastBGObs_value), Quantity.value(?breakfastBGObs_value, ?breakfastBGObs_value_dec), Quantity.code(?breakfastBGObs_value, milligram_per_deciliter), hasValue(?breakfastBGObs_value_dec, ?cbg), hasObservationValue(?prof, ?neededCarbs), neededToEatCarbs(?neededCarbs), isCurrent(?neededCarbs, true), Observation.valueQuantity(?neededCarbs, ?neededCarbs_value), quantity(?neededCarbs_value), Quantity.value(?neededCarbs_value, ?neededCarbs_value_dec), Quantity.code(?neededCarbs_value, gram), hasValue(?neededCarbs_value_dec, ?mc), hasCarePlan(?prof, ?cp), carePlan(?cp), CarePlan.status(?cp, carePlan_active), CarePlan.dailyPerMealGlucoseLevel.goal(?cp, ?premealGoal), goal(?premealGoal), Goal.target(?premealGoal, ?premealGoalComp), goalTargetComponent(?premealGoalComp), Goal.target.detailRange(?premealGoalComp, ?premealGoalComp_range), range(?premealGoalComp_range), Range.low(?premealGoalComp_range, ?premealGoalComp_range_low), quantity(?premealGoalComp_range_low), Quantity.value(?premealGoalComp_range_low, ?premealGoalComp_range_low_value), Quantity.code(?premealGoalComp_range_low, milligram_per_deciliter), hasValue(?premealGoalComp_range_low_value, ?low), Range.high(?premealGoalComp_range, ?premealGoalComp_range_high), quantity(?premealGoalComp_range_high), Quantity.value(?premealGoalComp_range_high, ?premealGoalComp_range_high_value), Quantity.code(?premealGoalComp_range_high, milligram_per_deciliter), hasValue(?premealGoalComp_range_high_value, ?high), swrlb:add(?pre_pbg, ?low, ?high), swrlb:divide(?pbg, ?pre_pbg, 2), swrlb:subtract(?DBG, ?cbg, ?pbg), swrlb:divide(?n1, ?DBG, ?isf_v), swrlb:divide(?n2, ?mc, ?icr_v), swrlb:add(?md, ?n1, ?n2), CarePlan.activity(?cp, ?cp_activity), carePlanActivityComponent(?cp_activity), CarePlan.activity.reference(?cp_activity, ?nutr), nutritionOrder(?nutr), NutritionOrder.meal(?nutr, ?meal), dinner(?meal) -> Meal.insulinUnitsForCarbs(?meal, ?n2), Meal.correctionInsulinUnits(?meal, ?n1), meal.totalBolusInsulinDosage(?meal, ?md)
88. patient(?p), patientProfile(?prof), hasPatientProfile(?p, ?prof), hasObservationValue(?prof, ?bmi), BMI(?bmi), Observation.valueQuantity(?bmi, ?quant2), quantity(?quant2), Quantity.value(?quant2, ?val2), decimal(?val2), hasValue(?val2, ?bmi_value), swrlb:lessThan(?bmi_value, 18.5) -> hasComplication(?prof, underWeight_condition)
89. patient(?p), patientProfile(?prof), hasPatientProfile(?p, ?prof), hasPreferedInsulinRegimen(?prof, "DP"^^xsd:string), carePlan(?cp), hasCarePlan(?prof, ?cp), FixedRegimen(?fg) -> hasInsulinRegimen(?cp, ?fg)
90. Dosage.route(?basal_dose, ?basal_dose_route), CodeableConcept.coding(?basal_dose_timing_codeable, ?basal_dose_timing_codeable_coding), quantity(?basal_dose_quant), hasCarePlan(?prof, ?cp), hasPreferedInsulinRegimen(?prof, "IIT"^^xsd:string), hasInsulinRegimen(?cp, ?ir), Coding.code(?basal_dose_timing_codeable_coding, ?basal_dose_timing_codeable_coding_code), hasPatientProfile(?p, ?prof), intensiveInsulinTherapy(?ir), timing(?basal_dose_timing), hasBasalMorningDose(?ir, ?basal_dose), Dosage.doseSimpleQuantity(?basal_dose, ?basal_dose_quant), codeableConcept(?basal_dose_route), Quantity.value(?basal_dose_quant, ?basal_dose_quant_decimal), Coding.display(?basal_dose_timing_codeable_coding, ?basal_dose_timing_codeable_coding_display), dosage(?basal_dose), codeableConcept(?basal_dose_timing_codeable), Coding.code(?basal_dose_route_coding, ?basal_dose_route_coding_code), Timing.code(?basal_dose_timing, ?basal_dose_timing_codeable), patientProfile(?prof), patient(?p), carePlan(?cp), Coding.display(?basal_dose_route_coding, ?basal_dose_route_coding_display), CodeableConcept.coding(?basal_dose_route, ?basal_dose_route_coding), coding(?basal_dose_route_coding), Dosage.timing(?basal_dose, ?basal_dose_timing), hasBasalInsulinUnits(?ir, ?final) -> hasValue(?basal_dose_timing_codeable_coding_code, "307473006"^^xsd:string), Quantity.code(?basal_dose_quant, units), hasValue(?basal_dose_route_coding_display, "injection"^^xsd:string), hasValue(?basal_dose_route_coding_code, "59108006"^^xsd:string), Coding.system(?basal_dose_route_coding, SNOMED_CT), Coding.system(?basal_dose_timing_codeable_coding, SNOMED_CT), hasValue(?basal_dose_quant_decimal, ?final), Quantity.system(?basal_dose_quant, UCUM_coding_system), hasValue(?basal_dose_timing_codeable_coding_display, "every evening"^^xsd:string)
91. patient(?p), patientProfile(?prof), hasPatientProfile(?p, ?prof), hasPatientMedication(?prof, ?ms), medicationStatement(?ms), MedicationStatement.medicationReference(?ms, ?med), medication(?med), drugContradictWithFood(?med, ?food), nutrient(?food), hasCarePlan(?prof, ?cp), carePlan(?cp), CarePlan.activity(?cp, ?act), carePlanActivityComponent(?act), CarePlan.activity.reference(?act, ?nu_order), nutritionOrder(?nu_order) -> hasForbiddenFood(?prof, ?food), NutritionOrder.excludeFoodModifier(?nu_order, ?food)
92. patientHasNoRapidActingContradict(?p), patientProfile(?prof), hasPatientProfile(?p, ?prof), hasPreferedInsulinRegimen(?prof, "IIT"^^xsd:string), carePlan(?cp), hasCarePlan(?prof, ?cp), intensiveInsulinTherapy(?ir), hasInsulinRegimen(?cp, ?ir) -> hasBolusInsulin(?ir, aspart)
93. patientContradictWithGlulisine(?p), patientContradictWithAspart(?p), patientProfile(?prof), hasPatientProfile(?p, ?prof), hasPreferedInsulinRegimen(?prof, "IIT"^^xsd:string), carePlan(?cp), hasCarePlan(?prof, ?cp), intensiveInsulinTherapy(?ir), hasInsulinRegimen(?cp, ?ir) -> hasBolusInsulin(?ir, lispro)
94. adult(?p), educationalAchievement(?p, "lowEducation"^^xsd:string), hasEducationRecord(?p, ?er), educationRecord(?er), visual(?v), auditory(?a) -> hasLearningStyle(?er, ?v), hasLearningStyle(?er, ?a)
95. child(?p), hasEducationRecord(?p, ?er), visual(?v), games(?g) -> hasLearningStyle(?er, ?v), hasLearningStyle(?er, ?g)
96. glargine(?m2), medicationStatement(?ms), MedicationStatement.medicationReference(?ms, ?m), hasPatientProfile(?p, ?prof), hasPatientMedication(?prof, ?ms), patientProfile(?prof), medication(?m), drugContradictWithDrug(?m, ?m2), patient(?p) -> patientContradictWithGlargine(?p)
97. child(?p), patientProfile(?prof), carePlan(?cp), goal(?preMeal_g), hasCarePlan(?prof, ?cp), CarePlan.dailyPerMealGlucoseLevel.goal(?cp, ?preMeal_g), Goal.target(?preMeal_g, ?preMeal_tar), goalTargetComponent(?preMeal_tar), Goal.target.detailRange(?preMeal_tar, ?preMeal_range), range(?preMeal_range), Range.low(?preMeal_range, ?preMeal_quant_low), quantity(?preMeal_quant_low), Quantity.value(?preMeal_quant_low, ?decim_preMeal_low), decimal(?decim_preMeal_low), Range.high(?preMeal_range, ?preMeal_quant_high), quantity(?preMeal_quant_high), Quantity.value(?preMeal_quant_high, ?decim_preMeal_high), decimal(?decim_preMeal_high) -> Quantity.code(?preMeal_quant_low, milligram_per_deciliter), hasValue(?decim_preMeal_low, 90), Quantity.comparator(?preMeal_quant_low, comparator_code_greaterThanOrEqual), Quantity.code(?preMeal_quant_high, milligram_per_deciliter), hasValue(?decim_preMeal_high, 130), Quantity.comparator(?preMeal_quant_high, comparator_code_lessThanOrEqual)
98. patient(?p), patientProfile(?prof), hasPatientProfile(?p, ?prof), hasLifeStyle(?prof, "sedentary"^^xsd:string) -> hasActivityLevel(?prof, "1.2"^^xsd:double)
99. patient(?p), patientProfile(?prof), hasPatientProfile(?p, ?prof), hasComplication(?prof, ?c), condition(?c), Condition.disease(?c, ?dis), obesity(?dis), hasEducationRecord(?p, ?er), exerciseLearningTopic(?ml), insulinLearningTopic(?i), dietLearningTopic(?dit) -> hasLearningTopic(?er, ?ml), hasLearningTopic(?er, ?i), hasLearningTopic(?er, ?dit)
100. meal(?m), hasCarbsInsulinUnits(?m, ?c), hasCorrectionInsulinUnits(?m, ?i), swrlb:add(?total, ?c, ?i) -> hasTotalInsulin(?m, ?total)
101. patient(?p), patientProfile(?prof), hasPatientProfile(?p, ?prof), hasComplication(?prof, ?c), condition(?c), Condition.disease(?c, foot_ulcer), jogging(?exe) -> hasForbiddenExercise(?prof, ?exe)
102. isCurrent(?w, true), hasCarePlan(?prof, ?cp), nutritionOrderSupplementComponent(?supp), hasPatientProfile(?p, ?prof), Quantity.value(?quant1, ?val1), Quantity.value(?quant3, ?val3), NutritionOrder.supplement.type(?supp, ?supp_type), swrlb:subtract(?w_h_a, ?w_h, ?a_v), hasObservationValue(?prof, ?h), Person.gender(?p, gender_male), swrlb:multiply(?a_v, ?value2, 6.76), Coding.display(?supp_type_coding, ?supp_type_coding_display), Quantity.code(?quant1, pound), isCurrent(?h, true), quantity(?supp_quant), NutritionOrder.supplement.quantity(?supp, ?supp_quant), Quantity.code(?quant3, inch), swrlb:multiply(?w_v, ?value1, 6.2), hasValue(?val1, ?value1), hasValue(?val3, ?value3), swrlb:add(?pre_final, ?w_h_a, 66), Person.age(?p, ?ag), weightValue(?w), Quantity.value(?supp_quant, ?supp_quant_val), quantity(?quant1), Observation.valueQuantity(?w, ?quant1), quantity(?quant3), decimal(?supp_quant_val), CodeableConcept.coding(?supp_type, ?supp_type_coding), Coding.code(?supp_type_coding, ?supp_type_coding_code), swrlb:ceiling(?final, ?pre_final), CarePlan.activity.reference(?act, ?nu_order), codeableConcept(?supp_type), patientProfile(?prof), patient(?p), swrlb:multiply(?h_v, ?value3, 12.7), carePlanActivityComponent(?act), Observation.valueQuantity(?h, ?quant3), NutritionOrder.supplement(?nu_order, ?supp), hasObservationValue(?prof, ?w), CarePlan.activity(?cp, ?act), coding(?supp_type_coding), height(?h), swrlb:add(?w_h, ?w_v, ?h_v), nutritionOrder(?nu_order), hasValue(?ag, ?value2), decimal(?val3), decimal(?val1) -> hasValue(?supp_quant_val, ?final), Quantity.system(?supp_quant, UCUM_coding_system), hasValue(?supp_type_coding_display, "basal metabolic rate (observable entity)"^^xsd:string), Coding.system(?supp_type_coding, SNOMED_CT), Quantity.code(?supp_quant, kilocalorie), hasValue(?supp_type_coding_code, "165109007"^^xsd:string)
103. adolescent(?p), patientProfile(?prof), hasPatientProfile(?p, ?prof), carePlan(?cp), hasCarePlan(?prof, ?cp), CarePlan.status(?cp, carePlan_active), hasInsulinRegimen(?cp, ?ir), insulinRegimen(?ir), hasTotalDailyDose(?ir, ?tdd), hasInsulinToCarbohydrateRatio(?prof, ?icr), quantity(?icr), swrlb:divide(?result, 500, ?tdd), Quantity.value(?icr, ?icr_v), decimal(?icr_v) -> Quantity.code(?icr, carbs_per_unit), Quantity.comparator(?icr, comparator_code_equal), Quantity.value(?icr_v, ?result)
104. patient(?p), patientProfile(?prof), hasPatientProfile(?p, ?prof), hasLifeStyle(?prof, "moderately active"^^xsd:string) -> hasActivityLevel(?prof, "1.55"^^xsd:double)
105. patient(?p), Person.age(?p, ?ag), hasValue(?ag, ?value), swrlb:greaterThanOrEqual(?value, 10), swrlb:lessThanOrEqual(?value, 19) -> adolescent(?p)
106. patient(?p), Person.gender(?p, gender_female), condition(?cond), patientProfile(?prof), hasComplication(?prof, ?cond), hasPatientProfile(?p, ?prof), hypertension(?hyper), Condition.disease(?cond, ?hyper) -> forbiddenFromExercise(?p)
107. patient(?p), patientProfile(?prof), hasPatientProfile(?p, ?prof), hasPatientMedication(?prof, ?meds), medicationStatement(?meds), MedicationStatement.medicationReference(?meds, ?med), medication(?med), hasEducationRecord(?p, ?er), medicationLearningTopic(?em), insulinLearningTopic(?i) -> hasLearningTopic(?er, ?em), hasLearningTopic(?er, ?i)
108. patient(?p), (hasHistoryOfHyperglycemia min 1 xsd:integer)(?x), hasEducationRecord(?p, ?er), emergencyLearningTopic(?em), insulinLearningTopic(?i) -> hasLearningTopic(?er, ?em), hasLearningTopic(?er, ?i)
109. hasCarePlan(?prof, ?cp), weightValue(?obs), hasInsulinRegimen(?cp, ?ir), hasPatientProfile(?p, ?prof), hasObservationValue(?prof, ?obs), decimal(?val), patientProfile(?prof), Observation.valueQuantity(?obs, ?quant), patient(?p), carePlan(?cp), swrlb:multiply(?prefinal, ?value, 0.6), quantity(?quant), hasValue(?val, ?value), swrlb:ceiling(?final, ?prefinal), insulinRegimen(?ir), Quantity.code(?quant, kilogram), Quantity.value(?quant, ?val) -> hasTotalDailyDose(?ir, ?final)
110. diseaseContradictWithDrug(?dis, ?m), hasPatientProfile(?p, ?prof), hasComplication(?prof, ?cond), patientProfile(?prof), disease(?dis), medication(?m), condition(?cond), patient(?p), Condition.disease(?cond, ?dis), aspart(?m) -> patientContradictWithAspart(?p)
111. patient(?p), hasDiabetesDuration(?p, ?q), quantity(?q), Quantity.code(?q, years), Quantity.value(?q, ?val), decimal(?val), hasValue(?val, ?x), swrlb:lessThanOrEqual(?x, 2) -> patientInHoneymoonPeriod(?p)
112. patientHasNoLongActingContradict(?p), patientProfile(?prof), hasPatientProfile(?p, ?prof), hasPreferedInsulinRegimen(?prof, "IIT"^^xsd:string), carePlan(?cp), hasCarePlan(?prof, ?cp), intensiveInsulinTherapy(?ir), hasInsulinRegimen(?cp, ?ir) -> hasBasalInsulin(?ir, detemir)
113. patient(?p), patientProfile(?prof), hasPatientProfile(?p, ?prof), hasPreferFood(?prof, ?food), nutrient(?food), hasCarePlan(?prof, ?cp), carePlan(?cp), CarePlan.activity(?cp, ?act), carePlanActivityComponent(?act), CarePlan.activity.reference(?act, ?nu_order), nutritionOrder(?nu_order) -> NutritionOrder.foodPreferenceModifier(?nu_order, ?food)
114. patient(?p), patientProfile(?prof), hasPatientProfile(?p, ?prof), hasComplication(?prof, ?c), condition(?c), Condition.disease(?c, ?dis), renal_failure(?dis) -> forbiddenFromExercise(?p)
115. patient(?p), hasDiabetesDuration(?p, ?q), quantity(?q), Quantity.code(?q, years), Quantity.value(?q, ?val), decimal(?val), hasValue(?val, ?x), swrlb:greaterThan(?x, 2) -> patientNOTInHoneymoonPeriod(?p)
116. adolescent(?p), notForbiddenFromExercise(?p), patientProfile(?prof), hasPatientProfile(?p, ?prof), hasRecommendedExercise(?prof, ?exe), 'aerobic exercise'(?exe), carePlan(?cp), CarePlan.status(?cp, carePlan_active), hasCarePlan(?prof, ?cp), CarePlan.activity(?cp, ?cp_a), carePlanActivityComponent(?cp_a), CarePlan.activity.reference(?cp_a, ?exe_p), exercisePlan(?exe_p), exercisePlan.haspart(?exe_p, ?exe_p_p), exercisePlanComponent(?exe_p_p), exercisePlan.component.activityDuration(?exe_p_p, ?exe_p_p_duration), range(?exe_p_p_duration), Range.low(?exe_p_p_duration, ?exe_p_p_duration_q_low), quantity(?exe_p_p_duration_q_low), Quantity.value(?exe_p_p_duration_q_low, ?exe_p_p_duration_q_low_v), decimal(?exe_p_p_duration_q_low_v), exercisePlan.component.totalWeeklyDuration(?exe_p_p, ?total_weekly), quantity(?total_weekly), Quantity.value(?total_weekly, ?total_weekly_value), decimal(?total_weekly_value), exercisePlan.component.activityFrequency(?exe_p_p, ?frequency), timing(?frequency), Timing.repeat.boundsRange(?frequency, ?frequency_range), range(?frequency_range), Range.low(?frequency_range, ?frequency_range_low_q), quantity(?frequency_range_low_q), Quantity.value(?frequency_range_low_q, ?frequency_range_low_q_value) -> exercisePlan.subject(?exe_p, ?p), exercisePlan.isPartOf(?exe_p, ?cp), Quantity.code(?exe_p_p_duration_q_low, minutes), Quantity.system(?exe_p_p_duration_q_low, UCUM_coding_system), Quantity.comparator(?exe_p_p_duration_q_low, comparator_code_greaterThanOrEqual), hasValue(?exe_p_p_duration_q_low_v, 60), exercisePlan.component.intensity(?exe_p_p, intensity_level_moderate_to_severe), hasValue(?total_weekly_value, 180), Quantity.code(?total_weekly, minutes), Quantity.system(?total_weekly, UCUM_coding_system), Quantity.comparator(?total_weekly, comparator_code_greaterThanOrEqual), Quantity.code(?frequency_range_low_q, days_per_week), Quantity.comparator(?frequency_range_low_q, comparator_code_greaterThanOrEqual), hasValue(?frequency_range_low_q_value, 3), exercisePlan.component.exerciseType(?exe_p_p, ?exe)
117. patient(?p), patientProfile(?prof), hasPatientProfile(?p, ?prof), hasInsulinSensitivityFactor(?prof, ?isf), quantity(?isf), Quantity.value(?isf, ?isf_dec), hasValue(?isf_dec, ?isf_v), hasInsulinToCarbohydrateRatio(?prof, ?icr), quantity(?icr), Quantity.value(?icr, ?icr_dec), hasValue(?icr_dec, ?icr_v), hasObservationValue(?prof, ?breakfastBGObs), breakfastGlucoseObservationValue(?breakfastBGObs), isCurrent(?breakfastBGObs, true), Observation.valueQuantity(?breakfastBGObs, ?breakfastBGObs_value), quantity(?breakfastBGObs_value), Quantity.value(?breakfastBGObs_value, ?breakfastBGObs_value_dec), Quantity.code(?breakfastBGObs_value, milligram_per_deciliter), hasValue(?breakfastBGObs_value_dec, ?cbg), hasObservationValue(?prof, ?neededCarbs), neededToEatCarbs(?neededCarbs), isCurrent(?neededCarbs, true), Observation.valueQuantity(?neededCarbs, ?neededCarbs_value), quantity(?neededCarbs_value), Quantity.value(?neededCarbs_value, ?neededCarbs_value_dec), Quantity.code(?neededCarbs_value, gram), hasValue(?neededCarbs_value_dec, ?mc), hasCarePlan(?prof, ?cp), carePlan(?cp), CarePlan.status(?cp, carePlan_active), CarePlan.dailyPerMealGlucoseLevel.goal(?cp, ?premealGoal), goal(?premealGoal), Goal.target(?premealGoal, ?premealGoalComp), goalTargetComponent(?premealGoalComp), Goal.target.detailRange(?premealGoalComp, ?premealGoalComp_range), range(?premealGoalComp_range), Range.low(?premealGoalComp_range, ?premealGoalComp_range_low), quantity(?premealGoalComp_range_low), Quantity.value(?premealGoalComp_range_low, ?premealGoalComp_range_low_value), Quantity.code(?premealGoalComp_range_low, milligram_per_deciliter), hasValue(?premealGoalComp_range_low_value, ?low), Range.high(?premealGoalComp_range, ?premealGoalComp_range_high), quantity(?premealGoalComp_range_high), Quantity.value(?premealGoalComp_range_high, ?premealGoalComp_range_high_value), Quantity.code(?premealGoalComp_range_high, milligram_per_deciliter), hasValue(?premealGoalComp_range_high_value, ?high), swrlb:add(?pre_pbg, ?low, ?high), swrlb:divide(?pbg, ?pre_pbg, 2), swrlb:subtract(?DBG, ?cbg, ?pbg), swrlb:divide(?n1, ?DBG, ?isf_v), swrlb:divide(?n2, ?mc, ?icr_v), swrlb:add(?md, ?n1, ?n2), CarePlan.activity(?cp, ?cp_activity), carePlanActivityComponent(?cp_activity), CarePlan.activity.reference(?cp_activity, ?nutr), nutritionOrder(?nutr), NutritionOrder.meal(?nutr, ?meal), breakfast(?meal) -> Meal.insulinUnitsForCarbs(?meal, ?n2), Meal.correctionInsulinUnits(?meal, ?n1), meal.totalBolusInsulinDosage(?meal, ?md)
118. patient(?p), hasEducationRecord(?p, ?er), educationRecord(?er), hasLearningStyle(?er, ?red), reading(?red), hasLearningTopic(?er, ?top), insulinLearningTopic(?top) -> hasLearningCourse(?er, learningCourse_insulin_reading_insulinDosage), hasLearningCourse(?er, learningCourse_insulin_reading_insulinSideEffect), hasLearningCourse(?er, learningCourse_insulin_reading_insulinStorage), hasLearningCourse(?er, learningCourse_insulin_reading_insulinTypes), hasLearningCourse(?er, learningCourse_insulin_reading_type_1_diabetes), hasLearningCourse(?er, learningCourse_insulin_reading_what_is_insulin)
119. patient(?p), patientProfile(?prof), hasPatientProfile(?p, ?prof), hasComplication(?prof, ?c), condition(?c), Condition.disease(?c, ?dis), 'autonomic neuropathy'(?dis) -> forbiddenFromExercise(?p)
120. patient(?p), patientProfile(?prof), hasPatientProfile(?p, ?prof), hasPreferedInsulinRegimen(?prof, "DP"^^xsd:string), carePlan(?cp), hasCarePlan(?prof, ?cp), FixedRegimen(?ir), hasInsulinRegimen(?cp, ?ir), hasTotalDailyDose(?ir, ?tdd), dosage(?m_s), hasFixedTwoshotsMorningShortActingInsulinDose(?ir, ?m_s), Dosage.doseSimpleQuantity(?m_s, ?m_s_quant), quantity(?m_s_quant), Quantity.value(?m_s_quant, ?m_s_quant_decim), swrlb:divide(?m_factor_s, 2, 9), swrlb:multiply(?m_s_quant_decim_value, ?m_factor_s, ?tdd), swrlb:ceiling(?fin, ?m_s_quant_decim_value) -> hasValue(?m_s_quant_decim, ?fin)
121. adult(?p), isPragnant(?p, false), patientNOTInHoneymoonPeriod(?p), hasHistoryOfHypoglycemia(?p, ?hyp), swrlb:greaterThanOrEqual(?hyp, 3), condition(?cond1), hasComplication(?prof, ?cond1), Condition.disease(?cond1, ?dis), 'cardiovascular disease'(?dis), patientProfile(?prof), carePlan(?cp), goal(?a1c_g), hasCarePlan(?prof, ?cp), CarePlan.HbA1C.goal(?cp, ?a1c_g), Goal.target(?a1c_g, ?a1c_tar), goalTargetComponent(?a1c_tar), Goal.target.detailQuantity(?a1c_tar, ?a1c_quant), quantity(?a1c_quant), Quantity.value(?a1c_quant, ?decim), decimal(?decim), goal(?preMeal_g), DifferentFrom (?a1c_g, ?preMeal_g), DifferentFrom (?a1c_g, ?bedTime_g), CarePlan.dailyPerMealGlucoseLevel.goal(?cp, ?preMeal_g), goalTargetComponent(?preMeal_tar), Goal.target.detailRange(?preMeal_tar, ?preMeal_range), range(?preMeal_range), Range.low(?preMeal_range, ?preMeal_quant_low), quantity(?preMeal_quant_low), Quantity.value(?preMeal_quant_low, ?decim_preMeal_low), decimal(?decim_preMeal_low), Range.high(?preMeal_range, ?preMeal_quant_high), quantity(?preMeal_quant_high), Quantity.value(?preMeal_quant_high, ?decim_preMeal_high), decimal(?decim_preMeal_high), Goal.target(?preMeal_g, ?preMeal_tar) -> Quantity.code(?a1c_quant, percent), hasValue(?decim, 8.0), Quantity.comparator(?a1c_quant, comparator_code_lessThan), Quantity.code(?preMeal_quant_low, milligram_per_deciliter), hasValue(?decim_preMeal_low, 80), Quantity.comparator(?preMeal_quant_low, comparator_code_greaterThanOrEqual), Quantity.code(?preMeal_quant_high, milligram_per_deciliter), hasValue(?decim_preMeal_high, 120), Quantity.comparator(?preMeal_quant_high, comparator_code_lessThanOrEqual)
122. diseaseContradictWithDrug(?dis, ?m), hasPatientProfile(?p, ?prof), hasComplication(?prof, ?cond), detemir(?m), patientProfile(?prof), disease(?dis), medication(?m), condition(?cond), patient(?p), Condition.disease(?cond, ?dis) -> patientContradictWithGlargine(?p)
123. patientContradictWithGlulisine(?p), patientProfile(?prof), hasPatientProfile(?p, ?prof), hasPreferedInsulinRegimen(?prof, "DP"^^xsd:string), carePlan(?cp), hasCarePlan(?prof, ?cp), FixedRegimen(?ir), hasInsulinRegimen(?cp, ?ir) -> hasFixedTwoshotsMorningShortActingInsulin(?ir, aspart), hasFixedTwoshotsEveningShortActingInsulin(?ir, aspart)
124. Quantity.code(?quant3, meter), swrlb:multiply(?h_v, ?value3, 1.85), isCurrent(?w, true), hasCarePlan(?prof, ?cp), nutritionOrderSupplementComponent(?supp), hasPatientProfile(?p, ?prof), Quantity.value(?quant1, ?val1), Quantity.value(?quant3, ?val3), NutritionOrder.supplement.type(?supp, ?supp_type), swrlb:multiply(?a_v, ?value2, 4.676), swrlb:subtract(?w_h_a, ?w_h, ?a_v), hasObservationValue(?prof, ?h), Coding.display(?supp_type_coding, ?supp_type_coding_display), isCurrent(?h, true), quantity(?supp_quant), NutritionOrder.supplement.quantity(?supp, ?supp_quant), hasValue(?val1, ?value1), hasValue(?val3, ?value3), Person.age(?p, ?ag), weightValue(?w), Quantity.value(?supp_quant, ?supp_quant_val), quantity(?quant1), Observation.valueQuantity(?w, ?quant1), quantity(?quant3), decimal(?supp_quant_val), CodeableConcept.coding(?supp_type, ?supp_type_coding), Coding.code(?supp_type_coding, ?supp_type_coding_code), swrlb:ceiling(?final, ?pre_final), CarePlan.activity.reference(?act, ?nu_order), codeableConcept(?supp_type), patientProfile(?prof), patient(?p), carePlanActivityComponent(?act), Observation.valueQuantity(?h, ?quant3), swrlb:add(?pre_final, ?w_h_a, 655.1), NutritionOrder.supplement(?nu_order, ?supp), hasObservationValue(?prof, ?w), swrlb:multiply(?w_v, ?value1, 9.563), CarePlan.activity(?cp, ?act), Person.gender(?p, gender_female), coding(?supp_type_coding), Quantity.code(?quant1, kilogram), height(?h), swrlb:add(?w_h, ?w_v, ?h_v), nutritionOrder(?nu_order), hasValue(?ag, ?value2), decimal(?val3), decimal(?val1) -> hasValue(?supp_quant_val, ?final), Quantity.system(?supp_quant, UCUM_coding_system), hasValue(?supp_type_coding_display, "basal metabolic rate (observable entity)"^^xsd:string), Coding.system(?supp_type_coding, SNOMED_CT), Quantity.code(?supp_quant, kilocalorie), hasValue(?supp_type_coding_code, "165109007"^^xsd:string)
125. adult(?p), isPragnant(?p, true), patientProfile(?prof), carePlan(?cp), goal(?a1c_g), hasCarePlan(?prof, ?cp), CarePlan.HbA1C.goal(?cp, ?a1c_g), Goal.target(?a1c_g, ?a1c_tar), goalTargetComponent(?a1c_tar), Goal.target.detailQuantity(?a1c_tar, ?a1c_quant), quantity(?a1c_quant), Quantity.value(?a1c_quant, ?decim), decimal(?decim), goal(?preMeal_g), DifferentFrom (?a1c_g, ?preMeal_g), CarePlan.dailyPerMealGlucoseLevel.goal(?cp, ?preMeal_g), goalTargetComponent(?preMeal_tar), Goal.target.detailRange(?preMeal_tar, ?preMeal_range), range(?preMeal_range), Range.low(?preMeal_range, ?preMeal_quant_low), quantity(?preMeal_quant_low), Quantity.value(?preMeal_quant_low, ?decim_preMeal_low), decimal(?decim_preMeal_low), Range.high(?preMeal_range, ?preMeal_quant_high), quantity(?preMeal_quant_high), Quantity.value(?preMeal_quant_high, ?decim_preMeal_high), decimal(?decim_preMeal_high), Goal.target(?preMeal_g, ?preMeal_tar) -> Quantity.code(?a1c_quant, percent), hasValue(?decim, 6.5), Quantity.comparator(?a1c_quant, comparator_code_lessThan), Quantity.code(?preMeal_quant_low, milligram_per_deciliter), hasValue(?decim_preMeal_low, 90), Quantity.comparator(?preMeal_quant_low, comparator_code_greaterThanOrEqual), Quantity.code(?preMeal_quant_high, milligram_per_deciliter), hasValue(?decim_preMeal_high, 100), Quantity.comparator(?preMeal_quant_high, comparator_code_lessThanOrEqual)
126. hasCarePlan(?prof, ?cp), isCurrent(?w, true), Quantity.value(?w_quant, ?w_val), hasHighestIdealWeightInKG(?prof, ?hiw), coding(?desc_coding), hasPatientProfile(?p, ?prof), swrlb:greaterThan(?value1, ?hiw), quantity(?wgt_quant), Quantity.code(?w_quant, kilogram), codeableConcept(?desc), Goal.target.detailQuantity(?wgt, ?wgt_quant), quantity(?w_quant), CodeableConcept.coding(?desc, ?desc_coding), swrlb:divide(?factor, ?pre_factor, ?day), Quantity.value(?wgt_quant, ?wgt_quant_value), goalTargetComponent(?wgt), weightValue(?w), decimal(?w_val), Coding.display(?desc_coding, ?desc_coding_display), Goal.target(?wg, ?wgt), swrlb:subtract(?pre_final, ?value1, ?hiw), Quantity.value(?nu_order_quant, ?nu_order_quant_value), swrlb:subtract(?total_cal, ?curr_cal, ?factor), hasCaloriesForCurrentWeight(?prof, ?curr_cal), swrlb:multiply(?pre_factor, ?pre_final, 7700), quantity(?nu_order_quant), hasValue(?w_val, ?value1), CarePlan.activity.reference(?act, ?nu_order), patientProfile(?prof), daysToLoseOrGainWeight(?prof, ?day), patient(?p), carePlan(?cp), Goal.subject(?wg, ?p), NutritionOrder.dailyCalories(?nu_order, ?nu_order_quant), carePlanActivityComponent(?act), Observation.valueQuantity(?w, ?w_quant), Goal.description(?wg, ?desc), hasObservationValue(?prof, ?w), CarePlan.activity(?cp, ?act), CarePlan.weight.goal(?cp, ?wg), nutritionOrder(?nu_order), Coding.code(?desc_coding, ?desc_coding_code), swrlb:subtract(?final, ?value1, ?pre_final), decimal(?nu_order_quant_value) -> hasValue(?desc_coding_display, "weight reduction regimen"^^xsd:string), hasValue(?desc_coding_code, "388976009"^^xsd:string), hasValue(?wgt_quant_value, ?final), Quantity.system(?wgt_quant, UCUM_coding_system), Quantity.code(?nu_order_quant, kilocalorie_per_day), Quantity.code(?wgt_quant, kilogram), Quantity.system(?nu_order_quant, UCUM_coding_system), hasValue(?nu_order_quant_value, ?total_cal)
127. patient(?p), patientProfile(?prof), hasPatientProfile(?p, ?prof), hasPreferedInsulinRegimen(?prof, "IIT"^^xsd:string), carePlan(?cp), hasCarePlan(?prof, ?cp), intensiveInsulinTherapy(?iit) -> hasInsulinRegimen(?cp, ?iit)
128. patient(?p), Person.gender(?p, gender_female), condition(?cond), patientProfile(?prof), hasComplication(?prof, ?cond), hasPatientProfile(?p, ?prof), 'morbid obesity'(?obese), Condition.disease(?cond, ?obese) -> forbiddenFromExercise(?p)
129. patient(?p), patientProfile(?prof), hasPatientProfile(?p, ?prof), hasComplication(?prof, ?c), condition(?c), Condition.disease(?c, ?dis), overweight(?dis), hasEducationRecord(?p, ?er), exerciseLearningTopic(?ml), insulinLearningTopic(?i), dietLearningTopic(?dit) -> hasLearningTopic(?er, ?ml), hasLearningTopic(?er, ?i), hasLearningTopic(?er, ?dit)
130. patient(?p), patientProfile(?prof), hasPatientProfile(?p, ?prof), hasObservationValue(?prof, ?h), height(?h), isCurrent(?h, true), Observation.valueQuantity(?h, ?h_quant), quantity(?h_quant), Quantity.code(?h_quant, meter), Quantity.value(?h_quant, ?h_val), decimal(?h_val), hasValue(?h_val, ?value), swrlb:multiply(?value_2, ?value, ?value), swrlb:multiply(?low_iw, 18.5, ?value_2), swrlb:multiply(?high_iw, 25, ?value_2) -> hasHighestIdealWeightInKG(?prof, ?high_iw), hasLowestIdealWeightInKG(?prof, ?low_iw)
131. patient(?p), patientProfile(?prof), hasPatientProfile(?p, ?prof), hasPreferedInsulinRegimen(?prof, "IIT"^^xsd:string), carePlan(?cp), hasCarePlan(?prof, ?cp), intensiveInsulinTherapy(?ir), hasInsulinRegimen(?cp, ?ir), hasTotalDailyDose(?ir, ?tdd), swrlb:multiply(?dose, ?tdd, "0.5"^^xsd:double), swrlb:ceiling(?final, ?dose) -> hasBasalInsulinPercentage(?ir, "0.5"^^xsd:double), hasBasalInsulinUnits(?ir, ?final), hasBolusInsulinPercentage(?ir, "0.5"^^xsd:double), hasBolusInsulinUnits(?ir, ?final), hasWeightToTDDfactor(?ir, "0.6"^^xsd:double)
132. hasCarePlan(?prof, ?cp), hasPreferedInsulinRegimen(?prof, "IIT"^^xsd:string), hasInsulinRegimen(?cp, ?ir), swrlb:divide(?pre_final, ?f, 3), hasPatientProfile(?p, ?prof), CodeableConcept.coding(?lunch_dosage_route, ?lunch_dosage_route_coding), intensiveInsulinTherapy(?ir), dosage(?lunch_dosage), Coding.code(?lunch_dosage_route_coding, ?lunch_dosage_route_coding_code), coding(?lunch_dosage_route_coding), codeableConcept(?lunch_dosage_route), quantity(?lunch_dosage_quant), codeableConcept(?lunch_dosage_timing_codeable), hasBolusInsulinUnits(?ir, ?f), Dosage.doseSimpleQuantity(?lunch_dosage, ?lunch_dosage_quant), Coding.display(?lunch_dosage_route_coding, ?lunch_dosage_route_coding_display), hasBolusLunchDose(?ir, ?lunch_dosage), Coding.code(?lunch_dosage_timing_codeable_coding, ?lunch_dosage_timing_codeable_coding_code), swrlb:ceiling(?final, ?pre_final), patientProfile(?prof), Dosage.route(?lunch_dosage, ?lunch_dosage_route), Dosage.timing(?lunch_dosage, ?lunch_dosage_timing), CodeableConcept.coding(?lunch_dosage_timing_codeable, ?lunch_dosage_timing_codeable_coding), patient(?p), carePlan(?cp), timing(?lunch_dosage_timing), Timing.code(?lunch_dosage_timing, ?lunch_dosage_timing_codeable), Quantity.value(?lunch_dosage_quant, ?lunch_dosage_quant_decimal), Coding.display(?lunch_dosage_timing_codeable_coding, ?lunch_dosage_timing_codeable_coding_display) -> hasValue(?lunch_dosage_route_coding_display, "injection"^^xsd:string), Coding.system(?lunch_dosage_timing_codeable_coding, SNOMED_CT), Quantity.code(?lunch_dosage_quant, units), hasValue(?lunch_dosage_quant_decimal, ?final), hasValue(?lunch_dosage_route_coding_code, "59108006"^^xsd:string), Quantity.system(?lunch_dosage_quant, UCUM_coding_system), Coding.system(?lunch_dosage_route_coding, SNOMED_CT), hasValue(?lunch_dosage_timing_codeable_coding_display, "daily with lunch"^^xsd:string), hasValue(?lunch_dosage_timing_codeable_coding_code, "1761000175102"^^xsd:string)
133. patientContradictWithGlulisine(?p), patientProfile(?prof), hasPatientProfile(?p, ?prof), hasPreferedInsulinRegimen(?prof, "IIT"^^xsd:string), carePlan(?cp), hasCarePlan(?prof, ?cp), intensiveInsulinTherapy(?ir), hasInsulinRegimen(?cp, ?ir) -> hasBolusInsulin(?ir, aspart)
134. oldAdult(?p), hasEducationRecord(?p, ?er), educationRecord(?er), visual(?v), auditory(?a) -> hasLearningStyle(?er, ?v), hasLearningStyle(?er, ?a)
135. patientContradictWithAspart(?p), patientProfile(?prof), hasPatientProfile(?p, ?prof), hasPreferedInsulinRegimen(?prof, "DP"^^xsd:string), carePlan(?cp), hasCarePlan(?prof, ?cp), FixedRegimen(?ir), hasInsulinRegimen(?cp, ?ir) -> hasFixedTwoshotsMorningShortActingInsulin(?ir, glulisine), hasFixedTwoshotsEveningShortActingInsulin(?ir, glulisine)
136. patient(?p), patientProfile(?prof), hasPatientProfile(?p, ?prof), medicationStatement(?ms), hasPatientMedication(?prof, ?ms), medication(?m), MedicationStatement.medicationReference(?ms, ?m), drugContradictWithDrug(?m, ?m2), lispro(?m2) -> patientContradictWithLispro(?p)
137. patient(?p), patientProfile(?prof), hasPatientProfile(?p, ?prof), encounter(?enc), hasEncounter(?prof, ?enc), observationValue(?ov), quantity(?quant), Observation.valueQuantity(?ov, ?quant), Quantity.code(?quant, kilogram_per_square_meter), Quantity.value(?quant, ?value), decimal(?value), hasValue(?value, ?x), swrlb:greaterThanOrEqual(?x, 30), Observation.context(?ov, ?enc), condition(?cond), obesity(?obesity), Condition.disease(?cond, ?obesity) -> hasComplication(?prof, ?cond)
138. Quantity.code(?quant3, meter), isCurrent(?w, true), quantity(?quant2), hasPatientProfile(?p, ?prof), Quantity.value(?quant1, ?val1), Quantity.value(?quant3, ?val3), hasObservationValue(?prof, ?h), isCurrent(?h, true), decimal(?val2), swrlb:divide(?bmi_value, ?value1, ?h_quare), hasValue(?val1, ?value1), hasValue(?val3, ?value3), swrlb:multiply(?h_quare, ?value3, ?value3), weightValue(?w), quantity(?quant1), Observation.valueQuantity(?w, ?quant1), quantity(?quant3), Quantity.value(?quant2, ?val2), patientProfile(?prof), BMI(?bmi), Observation.valueQuantity(?bmi, ?quant2), patient(?p), hasObservationValue(?prof, ?bmi), Observation.valueQuantity(?h, ?quant3), hasObservationValue(?prof, ?w), Quantity.code(?quant1, kilogram), height(?h), decimal(?val3), decimal(?val1) -> Quantity.code(?quant2, kilogram_per_square_meter), Quantity.system(?quant2, UCUM_coding_system), isCurrent(?bmi, true), hasValue(?val2, ?bmi_value)
139. patient(?p), patientProfile(?prof), hasPatientProfile(?p, ?prof), hasObservationValue(?prof, ?breakfastBGObs), glucoseLevelValue(?breakfastBGObs), isCurrent(?breakfastBGObs, true), Observation.valueQuantity(?breakfastBGObs, ?breakfastBGObs_value), quantity(?breakfastBGObs_value), Quantity.value(?breakfastBGObs_value, ?breakfastBGObs_value_dec), Quantity.code(?breakfastBGObs_value, milligram_per_deciliter), hasValue(?breakfastBGObs_value_dec, ?cbg), swrlb:lessThan(?cbg, 80) -> forbiddenFromExercise(?p)
140. patient(?p), patientProfile(?prof), hasPatientProfile(?p, ?prof), hasPreferedInsulinRegimen(?prof, "DP"^^xsd:string), carePlan(?cp), hasCarePlan(?prof, ?cp), hasFixedTwoshotsEveningIntermActingInsulinDose(?ir, ?e_l), FixedRegimen(?ir), hasInsulinRegimen(?cp, ?ir), hasTotalDailyDose(?ir, ?tdd), dosage(?e_l), Dosage.doseSimpleQuantity(?e_l, ?e_l_quant), quantity(?e_l_quant), Quantity.value(?e_l_quant, ?e_l_quant_decim), swrlb:divide(?e_factor_l, 1, 6), swrlb:multiply(?e_l_quant_decim_value, ?e_factor_l, ?tdd), swrlb:ceiling(?fin, ?e_l_quant_decim_value) -> hasValue(?e_l_quant_decim, ?fin)
